# 34th Annual Meeting of the Society for Light Treatment and Biological Rhythms (SLTBR), 30 May–1 June, Lausanne, Switzerland

**DOI:** 10.3390/clockssleep5030031

**Published:** 2023-08-24

**Authors:** Christian Cajochen

**Affiliations:** Centre for Chronobiology, Research Platform Molecular and Cognitive Neurosciences, Psychiatric Hospital of the University of Basel, Wilhelm-Kleinstr. 27, CH-4002 Basel, Switzerland; christian.cajochen@upk.ch; Tel.: +41-61-325-53-18; Fax: +41-61-325-55-56

## 1. Introduction

The Society for Light Treatment and Biological Rhythms (SLTBR) held this year’s annual meeting at the Ecole Polytechnique Fédérale (EPFL) in Lausanne, Switzerland from 30 May to 1 June in conjunction with the Day Light Academy (DLA).

I am delighted to report that our meeting has seen a surge in attendance, particularly from early career researchers. This overwhelming response speaks volumes about the importance of our shared mission and the growing interest in exploring the profound impact of light on human health and well-being. I would like to thank the 38 speakers who gave high-quality presentations of their latest original research on light and biological rhythms in areas as diverse as immunology, cancer chronotherapy, hepatology, mood disorders, psychotic disorders, premenstrual and perinatal depression, neurodegenerative diseases, neonatal intensive care, and an excellent keynote by Prof. Debra Skene on metabolic profiling of shift work. In addition to the oral presentations, we had many posters and data blitzes that attracted great interest from attendees, not forgetting the excellent Year in Review session. For the first time, we included a symposium on technology and innovation in chronobiology and sleep and a panel discussion on resilience in science in the Young Investigator Meeting.

A highlight this year was the joint symposium with the DLA in the famous Rolex building at EPFL, where we engaged in a translational dialogue on the ubiquitous importance of daylight in architecture, plant and urban sciences, chronobiology, medicine, etc. This collaborative approach to unravelling the mysteries of daylight is unique.

In addition to the stimulating sessions and engaging discussions, we had the opportunity to visit the enlightening “Lighten Up” exhibition at EPFL. This exhibition showcased innovative and artistic applications of light and demonstrated its potential to transform various aspects of our lives. I think there is no better way to present our DLA and SLTBR mission goals in a very attractive and thought-provoking way.

It was great to see so many motivated people networking in the bright sunshine of Lausanne, and we look forward to the next annual meeting in Prague in 2024. Please enjoy reading the very intriguing contributions to this year’s SLTBR meeting from poster, oral, symposium and invited speaker presentations–a mix of original data, study protocols and reviews.

Christian Cajochen, PhD

President SLTBR

## 2. Conference Abstracts

### 2.1. Light Affects Behavioral Despair Involving the Clock Gene Period 1

AlbrechtUrsUniversity of Fribourg, Fribourg, Switzerland

**Abstract:** Light at night has strong effects on the physiology and behavior of mammals. It affects mood in humans, which is exploited as light therapy, and has been shown to reset the circadian clock in the suprachiasmatic nuclei (SCN). This resetting is paramount to align physiological and biochemical timing to the environmental light–dark cycle. Here, we provide evidence that light at zeitgeber time (ZT) 22 affects mood-related behaviors also in mice by activating the clock gene *Period1* (*Per1*) in the lateral habenula (LHb), a brain region known to modulate mood-related behaviors. We show that complete deletion of *Per1* in mice led to depressive-like behavior and loss of the beneficial effects of light on this behavior. In contrast, specific deletion of *Per1* in the region of the LHb did not affect mood-related behavior, but suppressed the beneficial effects of light. RNA sequence analysis in the mesolimbic dopaminergic system revealed profound changes in gene expression after a light pulse at ZT22. In the nucleus accumbens (NAc), sensory perception of smell and G-protein-coupled receptor signaling were affected the most. Interestingly, most of these genes were not affected in *Per1* knockout animals, indicating that induction of *Per1* by light serves as a filter for light-mediated gene expression in the brain. Taken together, the data show that light affects mood-related behavior in mice, at least in part via induction of *Per1* in the LHb, with consequences on mood-related behavior and signaling mechanisms in the mesolimbic dopaminergic system.

### 2.2. In Search of the Optimal Treatment Components for Insomnia Using the Multiphase Optimization Strategy: Development of a Smartphone-Delivered CBT-I

AmidiAli^1^KnutzenSofie Møgelberg^1^ChristensenDinne Skjærlunde^1^DamholdtMalene Flensborg^2^ZachariaeHugh James Robert^3^1Sleep and Circadian Psychology Research Group, Department of Psychology and Behavioural Sciences, Aarhus University, Aarhus, Denmark2Unit for Psychooncology and Health Psychology (EPoS), Department of Psychology and Behavioural Sciences, Aarhus University, Aarhus, Denmark3Unit for Psychooncology and Health Psychology (EPoS), Sleep and Circadian Psychology Research Group, Department of Psychology and Behavioural Sciences, Aarhus University, Aarhus, Denmark

**Abstract: Background**: Insomnia is a highly prevalent disorder, with 10% of the general population experiencing sleep difficulties and 6–10% meeting diagnostic criteria for chronic insomnia. Although prescription of hypnotics is the most common treatment approach, due to severe side effects, dependence, and tolerance, cognitive behavioral therapy for insomnia (CBT-I) is recommended as first-choice treatment for the treatment of insomnia. Traditional CBT-I consists of multiple treatment components targeting both homeostatic and circadian factors, and includes sleep hygiene education, sleep restriction therapy, stimulus control therapy, relaxation training, and cognitive therapy. Unfortunately, due to the limited availability of trained therapists, many individuals suffering from insomnia do not receive the necessary treatment. Digitally delivered CBT-I (eCBT-I) has shown to provide a solution to this problem. While it is firmly established that CBT-I as a combined treatment, including digital adaptations, is an efficacious treatment of insomnia, less is known about whether an optimal combination of components exists, or if specific combinations may be more or less effective. To answer these questions, we have developed a research-based digital CBT-I app named hvil^®^. **Methods**: We are currently undertaking a large, preregistered trial (NCT05561829) to test the overall efficacy of hvil^®^ and to evaluate the individual treatment components employing a multiphase optimization strategy (MOST). In a 2^4^ fractional factorial design, 860 participants will be randomized to 16 conditions with different combinations of the treatment components. The primary outcome is insomnia severity measured with the Insomnia Severity Index. Secondary outcomes include sleep quality, sleep diary outcomes, fatigue, depression, and anxiety. All outcomes will be measured at pre- and post-intervention and at a 6-month follow-up. **Development of hvil^®^**: Hvil^®^, a research-based and fully automated smartphone app for delivering eCBT-I, was developed in close collaboration with a Danish health-tech company. Great care was taken to create stand-alone treatment components that were able to be flexibly combined while ensuring an optimal user experience. All enrolled participants will receive one or more components via the hvil^®^ app and the intervention will last between six to nine weeks. **Results**: We anticipate that the results will demonstrate the relative efficacy of the five treatment components in improving sleep and sleep-related outcomes. The results will enable us to test whether specific treatment components contribute significantly to treatment outcomes, allowing us to determine an optimal treatment combination when treating insomnia digitally. **Conclusions and implications**: As the first of its kind in the context of digital CBT-I, this MOST trial will demonstrate the relative efficacy of traditional CBT-I treatment components when delivered via a smartphone application. Depending on the efficacy of the individual or combined components, the most efficacious combination will need further development and testing in a randomized trial. The project is expected to have major implications for the future of insomnia treatment. If we find support for the efficacy of hvil^®^ employing some or all of its treatment components, the application will facilitate access to behavioral treatment for insomnia with the potential to reduce the individual and societal burden of the disorder.

### 2.3. From Dawn to Dusk—Mimicking Natural Daylight Exposure Improves Circadian Rhythm Entrainment in Patients with Severe Brain Injury

AngererMonika^1^PichlerGerald^2^AngererBirgit^3^ScarpatettiMonika^2^SchabusManuel^1^BlumeChristine^4^1Laboratory for Sleep, Cognition and Consciousness Research, Centre for Cognitive Neuroscience Salzburg (CCNS), Department of Psychology, University of Salzburg, Salzburg, Austria2Apallic Care Unit, Albert Schweitzer Hospital, Geriatric Health Care Centres of the City of Graz, Graz, Austria3Private Practice for General Medicine and Neurology, Leibnitz, Austria4Centre for Chronobiology, Psychiatric Hospital of the University of Basel, Transfaculty Research Platform Molecular and Cognitive Neurosciences, University of Basel, Basel, Switzerland

**Abstract: Objectives**: In post-comatose patients with disorders of consciousness (DOCs), circadian (~24 h) rhythms are often altered, with the degree of disturbance being related to the patients’ clinical state. Light therapy has proven effective in ameliorating certain medical conditions (e.g., circadian rhythm sleep–wake disorders). However, its potential has not yet been evaluated systematically in patients with DOCs, whose ‘light diet’ is usually characterised by fairly low illuminance during the day and relatively high illuminance during the night. Consequently, daytime melanopic light exposure is mostly insufficient for an optimal modulation of the circadian system. Thus, the aim of the present study was to investigate if indoor room lighting, which mimics natural daylight regarding its spectral composition and changes in illuminance, can re-entrain circadian rhythms in patients with DOC and consequently improve their clinical state. **Methods**: We recorded skin temperature over 7–8 consecutive days in patients with unresponsive wakefulness syndrome (*n* = 15) or minimally conscious state (*n* = 2) in each of two light conditions. In the habitual light (HL) condition, patients were in a room with standard lighting. In the dynamic daylight condition (DDL), patients were in a room with ‘biodynamic’ lighting that was characterised by overall higher illuminance and dynamic variations in spectral composition as they occur in natural daylight. To detect rhythmicity in the patients’ temperature data, we computed Lomb–Scargle periodograms (normalised power and period length), as well as interday stability and intraday variability, which provide information about rhythm entrainment and fragmentation. For statistical analyses, we used advanced non-parametric statistical tests as implemented in the ‘nparLD’ package for R. **Results**: In the DDL compared to the HL condition, patients’ temperature rhythms deviated less from 24 h (median deviation from 24 h: DDL = 0.52 h, HL = 3.62 h), were more pronounced, more stable and less fragmented. Behaviourally, patients showed a higher reactivity to external stimuli, as indicated by a higher sum score during assessments with the Coma Recovery Scale—Revised in the DDL condition. **Conclusions**: Our findings indicate that adequate room lighting in intensive care units and long-term care facilities may be a promising and easy way to realize a therapeutic approach that helps to improve rhythm entrainment in severely brain-injured patients.

**Funding**: Austrian Science Fund (FWF Y-777, W1233-G17).

**Disclosure:** Nothing to disclose.

### 2.4. Timing Matters: Non-Visual Effects of Light on Brain Activity during Morning and Evening Sessions Using 7T MRI and an Auditory Oddball Task

BaldaFermin^1^CampbellIslay^1^BeckersElise^1^SherifSiya^1^PaparellaIlenia^1^BergerAlexandre^2^KoshmanovaEkaterina^1^MortazaviNasrin^1^PhilipsChristophe^1^LamalleLaurent^1^VandewalleGilles^1^1Sleep and Chronobiology Laboratory, GIGA-CRC-IVI, University of Liège, Liège, Belgium2Université Catholique de Louvain, Louvain, Belgium

**Abstract: Introduction**: With the discovery of new, non-rod, non-cone, intrinsically photosensitive retinal ganglion cells, new research shows that natural and artificial light regimes have the potential to weaken and strengthen cognition, alertness and attention. These effects are mediated in part by melanopsin-expressing intrinsically photosensitive retinal ganglion cells (ipRGCs) that, in contrast to the classical photopic system that is maximally sensitive to green light (550 nm), are very sensitive to blue light (470–480 nm). These photoreceptors not only stimulate alertness, attention, vitality, and cognitive performance but also influence our biological clock and sleep. By understanding how different light regimes affect the brain, we may be able to develop interventions that promote cognitive performance and improve sleep quality in various environments. Therefore, using high-resolution ultrahigh-field (7T) functional magnetic resonance imaging (fMRI), we aim at characterizing the neural correlates of the alerting effect of light and whether this impact changes with time of day. **Methods**: The study is ongoing, and at the time of submission, six young subjects (25.5 ± 3.2 years; 4 women) had completed the protocol consisting of the administration of an auditory oddball task, mimicking novelty detection and recruiting attentional processes, in the morning (2 h after habitual wake time) and in the evening (1 h before habitual sleep time). They were requested to detect rare (20%) deviant tones (100 Hz) among more frequent (80%) standard (500 Hz) ones by pressing a button with their right index finger. During the task, participants were exposed to 30-second blocks of blue enriched light (6500 K; 190 melanopic EDI lux) and orange monochromatic light (589 nm; 0.16 melanopic EDI lux) interleaved by ~10-second dark periods. **Results**: Following data preprocessing, we conducted a paired t-test using SPM12 to compare morning and evening sessions. When comparing target tones under blue versus orange light conditions, our results suggest that evening sessions were associated with higher activation in the pulvinar (t = 4.03, *p* < 0.005, uncorrected for multiple comparisons) compared to evening sessions. Conversely, we observed higher activation during morning sessions compared to morning sessions in an area of the anterior hypothalamus potentially compatible with the ventromedial hypothalamus (t = 4.03, *p* < 0.005, uncorrected for multiple comparisons). **Conclusions**: These preliminary findings suggest that the hypothalamus and pulvinar, two regions key to circadian rhythmicity, attention and/or alertness regulation, may be involved in modulating the impact of light on attention, cognition and alertness across different times of day. Future analyses on a larger sample will assess the validity of this tentative conclusion and further detail the mechanisms at stake.

**Keywords:** oddball; ultrahigh-field MRI (UHF MRI); blue light.

**Funding**: FNRS, ULiège, GIGA Doctoral School for Health Sciences, Fondation Léon Frédéric, LIGHTCAP EU-ETN-MSCA.

### 2.5. Frequency Analysis of Sustained Pupil Size under Various Melanopic Irradiance in Young Healthy Individuals

BeckersElise^1^CampbellIslay^1^SharifpourRoya^1^PaparellaIlenia^1^BergerAlexandre^2^BaldaFermin^1^KoshmanovaEkaterina^1^MortazaviNasrin^1^TalwarPuneet^1^SherifSiya^1^JacobsHeidi^3^VandewalleGilles^1^1Sleep and Chronobiology Laboratory, GIGA-CRC-IVI, University of Liège, Liège, Belgium2Université Catholique de Louvain, Louvain, Belgium3Faculty of Heath, Medicine and Life Sciences, School for Mental Health and Neuroscience, Alzheimer Centre Limburg, Maastricht University, Maastricht, The Netherlands

**Abstract: Background**: Light exerts many non-image-forming (NIF) functions through a photoreception system maximally sensitive to blue wavelength light. NIF functions include the stimulation of cognition and alertness and the regulation of pupil size through the so-called pupil light reflex (PLR). Previous work suggested that the NIF responses to light are mediated in part through the locus coeruleus (LC). Given that the LC is considered to contribute to variability in pupil size, the PLR might be an indirect means to assess LC contribution to NIF responses. Here, we investigated how light irradiance level, as indexed through melanopic (mel) EDI lux, affected fast short-time scale variability in sustained PLR. **Methods**: Pupil size was continuously recorded in 22 young healthy subjects (age 23.3 ± 4.3 years; 15 females) with an infrared eye-tracker device (Eye Link, SR Research, Ottawa, Canada; sampling rate: 1000 Hz) under alternating short light exposures (30–50 s) during three distinct cognitive processes (executive, emotional and attentional) in a magnetic resonance imaging (MRI) scanner. Light conditions pseudo-randomly alternated between monochromatic orange light (0.16 mel EDI lux; 589 nm) and polychromatic, blue-enriched light of three different irradiances (37, 92, 190 mel EDI lux; 6500 K). Statistics consisted of generalized linear mixed models seeking effects of light irradiance levels on power spectrum density (PSD) of pupil size variations within a light exposure and in the 0.5–4 Hz frequency range, with subjects as a random intercept and controlling for age, sex, BMI and time of day. **Results**: Higher melanopic irradiance was significantly associated with a smaller PSD of pupil size across all three cognitive tasks (main effect of melanopic irradiance level: Executive: F_(3,13)_ = 23.7, *p* < 0.0001, R^2^* = 0.85; Emotional: F_(3,16)_ = 130.75, *p* < 0.0001, R^2^* = 0.96; Attentional: F_(1,5)_ = 40.2, *p* = 0.0011, R^2^* = 0.89). Post hoc analyses yielded significant differences between all irradiance levels in the three tasks (*p*_corrected_ < 0.041), except for the lowest and the highest melanopic irradiance level during the executive task (*p_corrected_* > 0.093). **Conclusions**: These results indicate that fast short-time scale variability in sustained PLR is lowered with increasing melanopic irradiance, irrespective of the cognitive context. While this could suggest a decrease in the underlying tonic activity of the LC, it could also result in a change in the firing pattern of LC neurons.

**Keywords:** pupil light reflex; non-image-forming effects; light; locus coeruleus; melanopic irradiance

**Funding**: ULiège, UMaastricht, LIGHTCAP ETN MSCA, FNRS, FLF.

### 2.6. Clinical Associations with the Blood’s Molecular Oscillator, Revealed by ClinCirc and Other Methods

BlaikleyJohnUniversity of Manchester, Manchester, UK

**Abstract:** Circadian biology has the potential to impact patient care in a profound way, with the outcomes of patients varying by two- to fourfold depending on circadian factors. Epidemiological studies can often be challenging due to the potential for confounding variables requiring large cohorts of patients combined with multivariate analysis to produce accurate results. Linking these observations to alterations in circadian mechanisms e.g., molecular oscillator, has also proved to be difficult. This is because it is difficult to sequentially sample patients and there is a lack of mathematical models that can analyse sparsely sampled circadian data. To partially overcome these challenges, we developed a mathematical method (ClinCirc) and combined this with epidemiological observations. This revealed how the molecular oscillator is altered on critical care and the impact it has on patient outcome. We initially combined existing circadian analysis techniques (Lomb–Scargle periodogram and cosinor analysis) into a new analysis pathway called ClinCirc. In this pathway, the Lomb–Scargle periodogram identifies whether a circadian oscillation is present. The characteristics (phase and amplitude) of detected circadian oscillations are then described using cosinor analysis. Using this technique on sequentially collected blood samples from patients in critical care, we were able to ascertain that inflammation is associated with reduced detection of circadian oscillations in the molecular oscillator. This association was observed for 17 out of 37 measured inflammatory mediators, as well as for the routinely measured acute-phase C-reactive protein. Other circadian analysis methods did not reveal a similar association. Circadian oscillations of the blood’s molecular oscillator were also investigated in kidney transplantation, where patients are exposed to many of the same clinical stimuli but inflammation is suppressed due to the anti-rejection medication given during the procedure. In this population, detection of circadian rhythms was actually increased compared to healthy volunteers. Three circadian analysis methods (ClinCirc, Cosinor, Gaussian Regression) revealed that circadian rhythms were synchronised according to the time of day the kidney was reperfused during the transplant operation. This shift in circadian rhythms could potentially explain the circadian oscillation in early kidney transplant outcomes (delayed graft function). In conclusion, ClinCirc reveals changes in the molecular oscillator that were previously obscured using existing methods. ClinCirc can be obtained from https://figshare.com/s/700cedb00253847212ae.

### 2.7. The Effect of Melatonin in Treatment of Nocturnal Hypertension According to Circadian Rhythm of Patients

BolmammedovYklymHojageldiyevTaganmyratState Medical University of Turkmenistan Named after M. Garryyev, Ashgabat, Turkmenistan

**Abstract: Background**: Previous studies confirm that alterations in circadian rhythm of blood pressure (BP) such as non-dipping pattern have been associated with adverse cardiovascular outcomes. Melatonin, the primary circadian hormone, is a potential treatment for nocturnal hypertension and non-dipping, particularly for patients who have poor sleep quality, because of its BP-reducing effect by various mechanisms. Instead of its clinically approved hypotensive results, it is unclear whether melatonin shows the same BP reduction in different chronotypes of people. This study aimed to investigate the effect of oral melatonin for treating hypertension according to chronobiological aspects of patients. **Methods**: Patients with uncomplicated essential hypertension who were taking the same antihypertensive therapy according to a unique treatment protocol had their chronotypes assessed by the Horne–Ostberg questionnaire. This study included thirty-two morning-type and twenty-four evening-type patients. All patients received 5 mg melatonin at bedtime for a month. Patients were evaluated with 24 h ambulatory blood-pressure monitoring (ABPM) before and after the treatment with melatonin. On the basis of night/day ratio of nocturnal BP fall, patients were divided into groups such as dippers and non-dippers whose systolic or diastolic nocturnal blood pressure dropped less than 10%. Statistical analyses were performed using GraphPad Prism. **Results**: According to the results of ABPM, before the treatment, the number of non-dippers was 14 (43.8%) in morning types, and 17 (70.8%) patients were revealed as non-dippers in evening types. Melatonin intake restored to normal diurnal BP rhythm in 35.7% of non-dipper morning types, but in evening types, 76.5% of non-dippers achieved features of dippers. After treatment of non-dippers of both groups, decreases in diastolic, systolic and mean night blood pressure was significant (*p* < 0.05). **Conclusions**: The results of our study demonstrate that the effect of melatonin differs according to the chronotype of patients and is more effective for evening types by a twofold decrease in number of non-dippers compared to morning-types. Our results were also in agreement with previous studies reporting that melatonin decreases nocturnal hypertension significantly.

### 2.8. Regional Difference in Response to Light across the Human Hypothalamus

CampbellIslay^1^SharifpourRoya^1^BaldaFermin^1^BeckersElise^1^PaparellaIlenia^1^BergerAlexandre^2^KoshmanovaEkaterina^1^MortazaviNasrin^1^SherifSiya^1^LamalleLaurent^1^PhilipsChristophe^1^VandewalleGilles^1^1Sleep and Chronobiology Laboratory, GIGA-CRC-IVI, University of Liège, Liège, Belgium2Université Catholique de Louvain, Louvain, Belgium

**Abstract: Background**: The non-image-forming (NIF) light-sensitive photoreceptor pathway detects environmental irradiance and can trigger many NIF biological effects, including the stimulation of alertness and cognition. The NIF system is mainly mediated through intrinsically photosensitive retinal ganglion cells (ipRGCs) due to the expression of the photopigment melanopsin, which is maximally sensitive to blue wavelength light around 480 nm. IpRGCs innervate several subcortical brain regions, including the hypothalamus. Previous animal and human imaging studies have suggested that the hypothalamus reacts to changes in the light environment. Here, we investigated how different subparts of the hypothalamus react to short (<1 min) light exposure of different melanopic irradiances over the course of two different auditory cognitive tasks. We predicted that the hypothalamus subparts will react differently to melanopic light exposure. **Methods**: Twenty-nine healthy young participants (11 men, 24.2 ± 3 years) were included in the study. Participants first completed a structural 7 Tesla magnetic resonance imaging (7T MRI) scan in which high-resolution T1-weighted structural images of their brains were collected. They maintained a loose sleep–wake schedule (±1 h; verified with actigraphy) for 7 days before the functional MRI experiment. Participants came to the lab 2 h after their habitual wake time to undergo three consecutive 7T fMRI recordings, during which they completed auditory cognitive tasks spanning the executive, attentional and emotional cognitive domains whilst exposed to pseudo-random light blocks (<1 min) of polychromatic white, blue-enriched light (6500 K; 37, 92, 190 melanopic EDI lux) and monochromatic orange light (589 nm; 0.16 melanopic EDI lux). Following pre-processing, statistical analyses of the fMRI data consisted of two-step mixed-effect analyses seeking an impact of melanopic irradiance on the brain responses to the auditory tasks. A published machine learning approach was used to subdivide the hypothalamus into five subregions over which we extracted the impact of melanopic irradiance on activity estimates. Further statistical analysis consisted of generalised linear mixed models (GLMMs) seeking regional effects of melanopic irradiance level activity on the hypothalamus regions, with subjects as a random intercept and controlling for age, sex and BMI, and task condition. **Results**: Statistical analyses revealed for both tasks a main effect of the hypothalamus subregions (emotion: F_(4,243)_ = 11.43; *p* < 0.001; executive: F_(4,190)_ = 2.97; *p* = 0.02). Post hoc analyses showed that the posterior part of the hypothalamus, which includes part of the lateral hypothalamus (LH) and tuberomammillary bodies (TMN), reacted more strongly to increasing irradiance than the other four subparts (*p* < 0.05), in which we detected no indication of a modulatory effect of irradiance. Further analyses showed that the highest irradiance level triggered the strongest responses over the posterior part of the hypothalamus. **Conclusions**: Our results support that the different subparts of the hypothalamus react differently to irradiance changes induced by short light exposure (<1 min) during two different cognitive tasks. The posterior hypothalamus was activated more by higher melanopic light levels compared to the other subregions. Further analyses will attempt to specify whether the LH, TMN, or both nuclei are likely to drive the effect we observed.

**Funding**: FRS-FNRS, ULiège, EU MSCA, FEDER, Fondation Léon Fredericq.

### 2.9. Acute Effect of a Novel Lighting Source on Mood of Psychotic Disorder Outpatients

ČervenáKateřina^1^EvansováKatarína^1^,^2^JankůKarolína^1^MaierovaLenka^3^BendováZdeňka^1^KopřivováJana^1^,^2^1Sleep and Chronobiology Research Centre, National Institute of Mental Health, Klecany, Czech Republic23rd Faculty of Medicine, Charles University, Prague, Czech Republic3University Centre for Energy Efficient Buildings, Czech Technical University in Prague, Trinecka 1024, 27343 Bustehrad, Czech Republic

**Abstract: Background**: Psychotic disorders are characterised by disturbances in thinking, perception, emotions and behaviour. For positive symptoms of psychosis, which include delusions and hallucinations, antipsychotic drugs are traditionally and relatively reliably chosen in therapy. Therapy of negative symptoms of psychosis, such as affective flattening, anhedonia and avolition, is difficult despite the availability of pharmacotherapy and often requires additional therapeutic approaches. Bright-light therapy is one of the biological treatments that has proven successful in the treatment of psychiatric disorders, especially depression, but has been used only sporadically in the treatment of psychotic disorders. Therefore, in this pilot experiment, we sought to determine whether a 30 min morning exposure to electric lighting with unique daylight-mimicking properties provided by a prototype experimental bright-light therapy booth called the “Sun spa” would have a significant effect on mood, particularly negative emotions, in outpatients with psychotic disorders. **Methods**: The Sun spa is a cubic interior pavilion with a side of 2.5 m. The ceiling and upper half of one sidewall serve as the source of the light. It provides a corneal illuminance of approximately 3100 lx, which represents melanopic equivalent daylight illuminance > 3060 lx, with a CCT of 4500 K and a color-rendering index above 80. The light has a diffuse character and balanced spatial distribution with maximal peak luminance of 3500 cd/m^2^, which is similar to a slightly overcast sky. In this preliminary study, 28 outpatients with psychotic disorder were exposed to 30 min of experimental lighting inside the Sun spa from around 10:00 to 10:30 a.m. as part of their program at the National Institute of Mental Health Day Care Center. The center is aimed at people with experience of psychotic illness in recovery from usually first but also subsequent psychotic episodes. Patients completed the Stanford Sleepiness Scale (SSS) and Positive and Negative Affect Schedule (PANAS) immediately before entering and immediately after leaving the Sun spa. While some patients attended the Sun spa only once, many attended multiple times, but the maximum was once a week. The data collection started in March 2022 and is ongoing. Paired t-tests were used to compare the data obtained before and after the intervention. **Results**: A 30 min exposure to experimental lighting inside the Sun spa resulted in a significant reduction in negative affectivity, as assessed by the PANAS self-report questionnaire. However, neither positive affectivity nor subjective sleepiness measures were affected by the intervention. **Conclusions**: The reduction in negative affectivity after exposure to light is consistent with our own findings in healthy adolescents, as well as the findings of other studies showing reductions of negative emotions after both long-term and acute light exposure. Our results suggest that even 3000 lx is enough to produce a positive effect.

**Keywords:** phototherapy; full-spectrum light source; daylight-mimicking light source; psychotic disorder; circadian rhythmicity; mood

**Funding**: Technology Agency of the Czech Republic grant FW02020025 (Stable and mobile devices to support circadian synchronization, treatment and prevention of mental disorders through full-spectrum light phototherapy).

### 2.10. What Does the Public Think When Asked about Daylight Saving Time? Reconciling Chronobiological and Popular Perspectives

CooganAndrewMaynooth University, Maynooth, Ireland

**Abstract:** Scientific, public, and political discourse about the perennial changing of the clocks during the transitions into and out of daylight saving time (DST) is a touchstone issue for the translation of fundamental chronobiology into societal impacts. Whilst there appears to be general consensus amongst chronobiologists and sleep scientists that abolition of the clock changes and adoption or permanent standard time would be the most adaptive response from a sleep and broader health perspective, public perceptions and preferences may differ. In this talk, I will explore the empirical data on public attitudes to clock changes, and examine the conflicts between these and the position statement of various professional sleep research bodies. In particular, I will explore uncertainties about the switching process to DST versus the steady state of being in DST, how the public discriminates between DST and summer, the priorities of the public in terms of the clock changes, and the role of geography- and jurisdiction-specific political considerations in shaping clock-change preferences. I will then identify some challenges for the chronobiology community in better influencing public and political discourse in order to achieve optimal outcomes and translate our science into policy.

### 2.11. “Rise and Shine”: Effects of Morning Light Exposure on EEG Alpha Wave Activity in Patients with Insomnia

KählerHannah^1^,^2^ZeeuwJan de^1^,^2^BesFrederik^1^,^2^KunzDieter^1^,^2^1Sleep Research and Clinical Chronobiology, Institute of Physiology, Charité–Universitätsmedizin Berlin, Berlin, Germany2Clinic for Sleep and Chronomedicine, St. Hedwig-Hospital, Berlin, Germany

**Abstract: Background**: Light influences human behavior and physiology. Besides being the most important zeitgeber for the human biological clock, light is known to influence e.g., vigilance and alertness independently. The effects of light have predominantly been studied under nighttime or morning light exposure, which can induce a circadian phase shift. Patients with insomnia suffer from hyperarousal, sometimes being the cause and sometimes being a symptom. Altered EEG alpha activity is an indicator of hyperarousal in patients with insomnia. The aim of the study was to determine immediate and indirect effects of morning light on EEG alpha activity in patients with insomnia. **Methods**: Included were 17 patients (age: 53 ± 11 years; mean ± SD; 14 female) with a current polysomnographic diagnosis of chronic insomnia. Excluded were 15 patients due to medication intake and unconfirmed insomnia diagnosis after polysomnography (PSG). Patients underwent a standard 3-night PSG. After night 2, a 3 h light exposure of warm white lighting took place (160 lx, peak wavelength 470 nm, 163 melanopic lx) 2 h after the individual wake time. Five min waking EEGs with eyes open and 5 min waking EEG with eyes closed in the midmorning and in the evening were measured at six time points over three days (T1: midmorning day 1; T2: evening day 1; T3/4: midmorning day 2 before/during light exposure; T5: evening day 2; T6: midmorning day 3). Determined parameters of the EEG alpha activity were: (1) alpha spectral power, an indicator of alertness and arousal; (2) the alpha attenuation test (AAT), an indicator of vigilance and sleepiness; and (3) the alpha peak frequency (APF), an indicator of higher cognitive function and cognitive alertness. **Results**: EEG alpha power with eyes open showed a significant main effect for the daytime measures (χ^2^(3) = 9.918, *p* = 0.019, *n* = 17), with the lowest mean EEG alpha power during the light exposure (mean = 3.87 mV², SD = 3.12) indicating higher arousal. There was a significant difference in the APF between the evenings (*t* = 1.828, *p* = 0.043, *p*-two tailed = 0.086, *n* = 19), with a higher APF on the first evening (mean = 9.63 Hz, SD = 1.08) indicating higher arousal than on the second evening (mean = 9.42 Hz, SD = 0.98) indicating lower alertness. The EEG alpha power with eyes closed and the AAT showed no significant change over the six time points. **Conclusions**: Morning light exposure is a promising intervention to improve vigilance of insomnia patients with an acute sleep deficit during the day. It is an interesting finding that EEG alpha parameters changed to the effect of lower arousal in the evening after morning light exposure in insomnia patients. Data suggest that morning light exposure can improve cognitive hyperarousal of insomnia patients prior to sleep, which can possibly improve the symptoms and reduce psychological strain. Data suggest that the alpha peak frequency may prove to be an indicator of minor intraindividual long-lasting changes of cognitive arousal of the central nervous system in patients with insomnia.

### 2.12. Retino-Recipient Brain Regions in a Diurnal Rodent, Rhabdomys pumilio

AcharigeAsshen DedigamaOrlowska-FeuerPatrycjaDaveyMarthaRichardsonRoseLucasRobertMilosavljevicNinaBano-OtaloraBeatrizUniversity of Manchester, Manchester, UK

**Abstract:** Photic cues are well known for their role in vision, but their role in non-visual responses is as significant. Such photic cues orchestrate daily rhythms in physiology and behaviour by entraining the circadian clock as well as via direct innervation of relevant brain regions. Most of our knowledge relating light to its effects comes from research conducted in nocturnal rodents and there is a major gap in our understanding of light’s effect in diurnal models. Moreover, since humans occupy a diurnal temporal niche, utilising a diurnal model helps to bridge the translational gap between rodent and human research. To explore light-recipient brain regions in a diurnal model system, we chose the African four-striped mouse (*Rhabdomys pumilio*), a rodent exhibiting high levels of activity during the day, both in the field and within laboratory settings. We first set out to trace direct retinal projections in the brain of *R. pumilio* by using bilateral intravitreal injections of anterograde cholera toxin B subunit conjugated with fluorescent dyes. We found that a majority of retino-recipient brain areas are similar to those in the nocturnal mouse. We then investigated the neuronal activity of identified retino-recipient brain areas. We applied two types of 30 min light pulses, steady light and flickering light, to *R. pumilio* at night and measured c-fos expression across the brain. In comparison to control groups kept under constant darkness, animals exposed to light pulses indicated a measurable increase in c-fos expression, with a majority of these occurring within the SCN, IGL and LGN regions. We conclude that light induces a general increase in neural activity in *R. pumilio*. Further work will be required to determine the relationship between neural activity and behavioural responses following exposure to light across the brain.

### 2.13. The Daytime Effect of Light Directionality on Mood, Alertness, and Cognitive Performance

DerengowskiNikodemKnoopMartineVölkerStephanTechnische Universtität Berlin, Berlin, Germany

**Abstract:** The directionality of light has been proven to play a role in moderating the magnitude of NIF effects during nighttime. Essentially, different directionality of light translates to different parts of the retina being illuminated, thus targeting various classes of photoreceptors or their diverse densities throughout the retina. The literature indicates that nighttime light illumination of the inferior part of the retina provides stronger NIF stimulus (measured as melatonin secretion) compared to the superior part, and moreover, light illuminating the nasal part of the retina has a stronger effect compared to the light targeting the temporal part of the retina. But does it stand valid for other—non-melatonin-related—NIF response markers? And more importantly—does it stand valid in daytime conditions? Here, we hypothesize that similar effects can be achieved in the daytime hours, and especially during the natural drop in alertness in the early afternoon. In the poster, I am presenting the design and the results of a scientific experiment that investigated the effect of light directionality on daytime cognitive performance, alertness, and mood change. The experiment was performed in autumn and winter in 2022–2023 in Berlin, with a total of 40 participants. Five lighting conditions were designed to represent different light directionalities, while the light dose of 510 lx photopic (580 lx mEDI at 6400 K CCT) was kept constant between them, measured as vertical illuminance and mEDI at the eye. Each of the participants went through each of the conditions on separate occasions with a 7-day (or longer, in few instances) wash-off period between the sessions. Cognitive performance was assessed using PVT, GoNoGo and 2-Back auditory tests. Subjective alertness was assessed using the Karolinska Sleepiness Scale. Mood and light acceptance were assessed using subjective scales. Total time of one session was 120 min, out of which 46 min was exposure under the experimental light condition. Moreover, the experiment was focused on looking into the potential effects of light during the post-lunch dip phase, and therefore all sessions were scheduled in the early afternoon—either at 12:00 or at 14:00. The initial results did not show a significant effect of the different light directionality on the cognitive tasks’ performance, opening a discussion whether directionality is an important factor in the daytime conditions. However, detailed analysis of results of other dependent variables is ongoing and will be presented in a poster at the SLTBR conference.

### 2.14. Effect of Daytime Napping on Skin-Temperature Regulation and Sleep in Healthy Older Adults

DourteMarine^1^DeantoniMichele^1^ReytMathilde^1^BailletMarion^2^HaanStella de^2^MutoVincenzo^1^BerthomierChristian^3^PeigneuxPhilippe^4^HammadGregory^2^SchmidtChristina^2^1Sleep and Chronobiology Laboratory, GIGA-CRC-IVI and PsyNCog Research Unit, Faculty of Psychology, University of Liège, Liège, Belgium2Sleep and Chronobiology Laboratory, GIGA-CRC-IVI, University of Liège, Liège, Belgium3PHYSIP, Paris, France4UR2NF, Neuropsychology and Functional Neuroimaging Research Unit at CRCN—Center for Research in Cognition and Neurosciences and UNI—ULB Neurosciences Institute, Université Libre de Bruxelles (ULB), Bruxelles, Belgium

**Abstract: Introduction**: Sleep–wake cycle fragmentation in aging is reflected by an increasing incidence of daytime napping, putatively underlined by circadian rhythm alterations. Chronic napping in older adults has been associated with increased health risk factors and cognitive decline, such as a higher risk of developing Alzheimer’s disease. Here, we assessed circadian modulation of temperature in healthy older nappers and non-nappers, sleepiness, and sleep parameters (sleep efficiency, SE; and sleep onset latency, SOL). **Methods**: In sum, 58 healthy participants (69 + 5.5 years) were prospectively recruited according to their napping habits (no nap, *n* = 28; nap, *n* = 30). They took part in a 40 h multiple-nap constant routine protocol, encompassing 10 alternating cycles of 160 min wakefulness and 80 min sleep opportunity. Distal–proximal temperature gradient (DPG) was measured using iButtons. SE and SOL were derived from polysomnographic recordings during scheduled nap opportunities. The Karolina Sleepiness Scale was used to assess sleepiness three times per wake session (KSS, 30 ratings by subject). General linear mixed models and repeated-measures ANOVAs explored whether napping habits affect DPG, SE, SOL, and sleepiness over the course of the protocol. **Results**: A circadian modulation of SE was reported over the protocol (*p* < 0.001) Additionally, SE differed between nap groups: nappers displayed lower SE during nighttime sleep opportunities and higher during daytime sleep opportunities (ps < 0.05). DPG and sleepiness also showed a circadian modulation (ps < 0.005). When compared to non-nappers, nappers displayed a lesser increase in DPG in the transition from biological day to biological night (*p* < 0.001) and lower overall DPG during the second biological day (ps < 0.003). Furthermore, nappers had a lower DPG amplitude than non-nappers (*p* < 0.001). While there was no group difference in SOL (*p* = 0.41), there was a significant interaction between the nap group and the rise in DPG prior to sleep (*p* = 0.001), indicating that nappers have a steeper increase in DPG once allowed to sleep. Interestingly, sleepiness did not differ between groups (*p* = 0.79) during the protocol. **Conclusions**: Our results suggest that napping in the elderly is associated with an altered 24 h distribution of sleep efficiency and a concomitant change in DPG modulation. These findings could point to a bidirectional relationship between thermoregulation and sleep initiation and/or maintenance, which is modulated by chronic napping. Future analysis will focus on DPG modulation prior to sleep opportunities, as well as during the transition from sleep to wakefulness to assess sleep-dependent thermoregulatory changes according to the napping habits. More research is required to better understand the altered distribution of skin temperature in nappers and its possible consequences.

**Acknowledgments**: This study was supported by the European Research Council (ERC, ERCStG-COGNAP) under the European Union’s Horizon 2020 research and innovation program (grant agreement 757763). This study was also supported by the Fonds de la Recherche Scientifique (FNRS) under grant T.0220.20. C. Schmidt is a research associate, M. Deantoni is a FRIA grantee of the FNRS and M. Dourte is an Aspirant-FNRS grantee of the FNRS.

### 2.15. Bringing Light into the Night: Pilot Study on the Addition of Biodynamic Lighting in the Psychiatric Ward to Improve Vitality of Nurses during Shift Work

DruivenStellaTuinenDouwe vanKapMarkHaarmanBennoKamphuisJeanineDepartment of Psychiatry, University Medical Center Groningen, Groningen, The Netherlands

**Abstract: Background**: Many researchers have studied the effects of shift work on physical and psychological well-being. Results from these studies make clear that shift-work can cause sleep problems, increase the risk of metabolic syndrome and mental health problems, and even increase the chances of certain types of cancer. However, shift-work is an essential part of our current 24-7 society and unavoidable in hospitals. The problems caused by shift-work are, in part, caused by a mismatch between the external and internal circadian rhythm. Therefore, strategies for minimizing this mismatch may be extremely helpful. The environmental addition of artificial daylight during the night could be one of those strategies. Biodynamic lighting replicates the dynamic variations in daylight through artificial light and can be installed in any room. In our study, the effects on sleep (quality), sleepiness, motivation, concentration and overall well-being of biodynamic light in the workroom for nurses on a psychiatric ward will be investigated. Our aim is to explore whether biodynamic light could be a useful implementation in the hospital to increase the vitality of nurses working in shifts. **Methods**: Nurses of three different wards of the Psychiatry Department at the University Medical Center Groningen (The Netherlands) will be included in this study. Besides the installation of biodynamic lighting in the workroom of the nurses, they will receive e-learning at the beginning of the intervention with information about circadian rhythms and the optimal timing of sleep, eating and activities during shift-work. Measurements of sleep (quality), sleepiness, motivation and concentration during every shift (day, evening and night) will be obtained for 3 months in total: 1 month prior to the intervention (baseline), 1 month from the start of the intervention and the 6th month after the intervention. Each ward will receive a different intervention: ward 1: biodynamic lighting + e-learning + questionnaires, ward 2: e-learning + questionnaires, and ward 3: questionnaires. After the study period of 7 months, the measurements at baseline and follow-up will be compared between and within the different wards. **Results**: We aim to start collecting baseline data fall 2023. **Discussion**: Results of this pilot study will contribute to the possibility of further implementation of biodynamic lighting in the hospital to improve well-being of hospital workers.

### 2.16. Measuring Activity, Rhythm and Sleep with Actigraphy during Chronotherapy in the Clinical Treatment of Depression

DruivenStellaHaarmanBennoKamphuisJeanineSchoeversRobertRieseHarriëtteDepartment of Psychiatry, University Medical Center Groningen, Groningen, The Netherlands

**Abstract: Background**: Alterations in circadian rhythms, such as sleep problems, are often seen in mood disorders. Therapy that specifically targets the circadian rhythm is called chronotherapy. Chronotherapy has a rapid effect on depression, but use in the clinic is still limited. **Methods**: For this exploratory study, data was collected before, during and after chronotherapy. Chronotherapy consisted of repeated sleep deprivation (3 nights) with a recovery night in between and 2 weeks of morning light therapy on work days (10,000 lux, 30 min). Depressive symptoms (Inventory of Depressive Symptomatology—Self Report (IDS)) were assessed and actigraphy was used to collect activity (daily mean activity), rhythm (intra-daily variability (IV), inter-daily stability (IS)), sleep (midpoint of sleep (MSF), time in bed, sleep efficiency (SE), and fragmentation index (FI)) parameters. Variables were compared before and after chronotherapy for each participant and as a group. Response/nonresponse was calculated with a minimal clinically important difference (MCID) of 30% change in IDS score in the final week of chronotherapy compared to baseline. Differences in variables between responders and nonresponders were calculated at baseline. **Results**: Nine patients (four females) were included who were diagnosed with a major depressive disorder (five), bipolar disorder type 1 (three) and bipolar disorder type 2 (one). Median age was 58 years with a range of 38–69. Three patients were considered responders. After chronotherapy, a decrease was seen in IDS, IV and FI. Responders appeared to have a higher IDS, time in bed and FI and lower SE at baseline. **Conclusions**: In this exploratory study on a small sample, the results might support the understanding that chronotherapy stabilizes mood, circadian rhythm and sleep. Data further suggested that responders spend more time in bed, had a higher FI and lower SE. This indicates that patients with a more disrupted sleep pattern respond better to chronotherapy. It can be concluded that chronotherapy can be used in the clinic to collect data on activity, rhythm and sleep. Moreover, our results can help indicate variables of interest in the search for predictors of response to chronotherapy.

### 2.17. Beginning to See the Light: Optimizing Light Conditions in the Neonatal Intensive Care Unit

DudinkJeroenUMC Utrecht, Utrecht, The Netherlands

**Abstract:** The circadian timing system plays a vital role in optimizing health by temporally coordinating behavior and physiology. During mammalian gestation, the daily fluctuations in maternal body temperature, hormones, and nutrients synchronize fetal circadian rhythms. Circadian disruption during pregnancy negatively impacts developmental outcomes in offspring, highlighting the importance of robust 24 h rhythms over gestation. Preterm birth, before 37 weeks of gestation, interrupts the neonate’s synchronization to maternal cues, potentially leading to adverse effects. Recent evidence suggests that introducing robust light–dark cycles in the Neonatal Intensive Care Unit (NICU) has a positive effect on clinical outcomes, including weight gain and hospitalization time, compared to infants exposed to constant light or near-darkness. However, the biological basis for these effects and their relationship with the development of the circadian system is not fully understood. This lecture provides an overview of the effects of light–dark cycles on clinical outcomes in preterm neonates in the NICU and their alignment with the development of the circadian system.

### 2.18. Sleep and Light Interventions for Premenstrual, Peripartum and Perimenopausal Depression

ParryBarbaraMeliskaCharlesSorensonDianeMartinezFernandoLopezAnaDawesSharronElliottJeffreyHaugerRichardUniversity of California, San Diego, CA, USA

**Abstract: Background**: The aim of these studies was to test the hypothesis that sleep and light treatment improves mood and sleep in premenstrual, peripartum and menopausal depression by differentially altering melatonin-sleep timing phase-angle differences (PADs). In previous work, we found that plasma melatonin circadian rhythms were phase-delayed with respect to sleep in women with premenstrual dysphoric disorder (PMDD) during the symptomatic luteal vs. asymptomatic follicular menstrual cycle phase, postpartum and perimenopausal depression, but phase-advanced in women with depression during pregnancy, compared with normal control (NC) participants. We also observed improvement in mood and sleep with independent studies of wake therapy (formerly therapeutic sleep deprivation) and light treatment. In the presented studies, we sought to realign melatonin and sleep timing disturbances with critically timed combined sleep and light interventions: to phase-advance the abnormally delayed melatonin rhythms with respect to sleep in PMDD, postpartum and perimenopausal depression, and to phase-delay the abnormally advanced melatonin rhythms in pregnancy depression, as combined sleep and light interventions have more potent effects on circadian organization, mood and sleep: wake therapy potentiates light treatment effects, and light treatment maintains wake therapy effects. **Methods**: After IRB approval, at home 144 premenstrual, peripartum and perimenopausal depressed (DSM-5) participants—DPs (N = 73) or normal controls—NCs (N = 71) were randomized to a parallel trial of (1) a phase-delay intervention (PDI): 1-night early-night wake therapy—EWT (sleep 3–7 a.m.) + 1 (PMDD), 6 (peripartum) or 8 (perimenopause) weeks of evening (PM) bright white light—BWL (Litebook Advantage) for 60 min daily starting 90 min before habitual sleep time, vs. (2) a phase-advance intervention (PAI): 1-night late-night wake therapy—LWT (sleep 9 p.m.–1 a.m.) + 1–8 weeks (as above) of morning (AM) BWL for 60 min daily starting within 30 min of wake time. Primary outcome measures included mood (Structured Interview Guide Hamilton Rating Scale for Depression—Atypical Depression Supplement—SIGH-ADS), melatonin (2 overnight–6 pm to noon urine samples for 6-sulphatoxy melatonin (6-SMT) and objective (actigraphy) and subjective (Pittsburgh Sleep Quality Index-PSQI) sleep assessments. We calculated (cosinor analyses) 6-SMT Onset/Offset, Acrophase; sleep Onset/Offset times (SOT/SET); PADs analyzed by MANCOVA/ANOVA (covariate Morningness- Eveningness questionnaire-MEQ). **Results**: In PMDD, baseline atypical depressed mood correlated with phase-delayed 6-SMT Offset (*p* = 0.038), PAI vs. PDI improved mood (*p* = 0.002), correlated with phase-advanced 6-SMT Offset (*p* = 0.004) and reduced 6-SMT Offset_SOT PAD (*p* = 0.003). Pregnant DP mood improved after PDI vs. PAI (*p* = 0.016 MEQ covariate); postpartum DP mood improved after PAI vs. PDI (*p* = 0.019), correlated with phase-advanced 6-SMT Offset (*p* = 0.003) and Acrophase (*p* < 0.05). in Perimenopausal DP vs. NC; baseline 6-SMT Acrophase was phase-delayed (*p* = 0.015), PAI improved mood correlated with phase-advanced Acrophase (*p* = 0.013), and phase-advanced Offset (*p* = 0.042); PDI vs. PAI improved subjective sleep quality (PSQI global score, *p* < 0.05). Mood improved equally after 1, 2 and 8 treatment weeks (+70%, *p* = 0.007). **Conclusions**: Mood and sleep improved differentially in response to critically timed wake and light interventions in PMDD, peripartum and perimenopausal DP depending on distinguishing melatonin and sleep circadian phase disturbances characterizing each reproductive epoch. Combined wake/light therapy has antidepressant effects within 1–2 weeks.

### 2.19. Light Therapy and Associated Improvements in Mood, Sleep, and Circadian Rhythms in Individuals with Depression—A Randomized Control Trial

NixonAshley^1^StrikeMelanie^2^FotiMichael-Christopher^3^KoninckJoseph De^1^DouglassAlan^4^HigginsonCaitlin^4^LeveilleChloe^1^RobillardRebecca^1^1University of Ottawa, Ottawa, ON, Canada2The Royal Ottawa Mental Health Centre, Ottawa, ON, Canada3École des Technologies Supérieures, Montreal, QC, Canada4The Royal’s Institute of Mental Health Research, Ottawa, ON, Canada

**Abstract: Background**: The various depression subtypes and the numerous ways in which light can affect mood have clouded our understanding of the value of light therapy for non-seasonal depression. This study investigates whether the effects of light therapy are superior to that of a placebo. It also aims to uncover potential underlying mechanisms of action relating to sleep and circadian rhythms, as well as identify predictors of treatment response. **Methods**: A group of 27 young people experiencing a major depressive episode (mean ± SD: 24 ± 5.6; 11% male) underwent two weeks of active short-wavelength light therapy and two weeks of a placebo condition. This was a crossover design with a washout period of two weeks between conditions. Prior to the light therapy intervention, participants completed a semi-constant routine protocol. Participants were instructed to use light therapy glasses for a period of 30 min to an hour after their typical wake-up time. Self-reported and clinician-rated questionnaires on sleep and mood were conducted both before and after each intervention. Throughout the study, actigraphy and skin temperature were continuously monitored. **Results**: Upon completion of the study, over 50% of participants where uncertain or believed that they were assigned to an active infrared condition instead of a placebo infrared condition. There was a trend for a time—condition interaction (F_(1,22)_ = 4.2, *p* = 0.053, = 0.16) in which improvements in depression symptoms were slightly greater in the active compared to the placebo condition. There was a significant main effect of time on depression symptoms (reduction in depression symptoms from pre- to postintervention: F_(1,22)_ = 12.6, *p* = 0.002, = 0.36)), but no significant effect of condition (active vs. placebo). The degree of improvements in intrusive pre-sleep thoughts (*β* = 0.52, *p* = 0.035) and skin temperature rhythmicity (*β* = −1.07, *p* = 0.032) during the intervention was significantly correlated with improvements in mood. Short REM latency (*β* = −0.45, *p* = 0.047) and worst global subjective sleep (*β* = 0.53, *p* = 0.045) prior to the light therapy intervention were associated with improved mood. **Conclusions**: This study demonstrated that light therapy has a slight antidepressant effect on individuals with depression, but the degree of this effect may vary from one person to the next. The results indicated that the reduction in pre-sleep thoughts and the restoration of skin temperature rhythmicity may be involved in the mechanisms responsible for the antidepressant effects of light therapy. It is also possible that individuals with short REM latency and poor sleep may be more likely to respond positively to light therapy. Further research is needed to disentangle the heterogeneous response to light therapy in people with non-seasonal depression.

**Keywords**: light therapy; depression; circadian rhythms; sleep

**Funding**: The Centre for Sleep and Chronobiology, University Medical Research Funds, University of Ottawa.

### 2.20. Cortical Responses to Daytime Light Exposure: Investigating Objective EEG Markers in Metameric Light Conditions

GecerElifnaz^1^SmoldersKarin^1^KortYvonne de^1^SchlangenLuc^1^SchoffelenJan Mathijs^2^1Eindhoven University of Technology, Eindhoven, The Netherlands2Donders Institute for Brain, Cognition and Behaviour, Radboud University, Nijmegen, The Netherlands

**Abstract: Background**: Our understanding of the brain’s relationship with light has expanded remarkably after the discovery of the new type of photosensitive cells (ipRGCs) in the retina [1]. These effects of light, sometimes also referred as the non-image-forming (NIF) responses, encompass areas such as performance, vitality, alertness and mood [2,3,4,5]. The relationship of short-wavelength light, which is the type of light that ipRGCs are more sensitive to, and the regulation of circadian rhythms is better established for nocturnal studies. Research shows that light can induce moderations in momentary alertness and arousal, but the findings regarding the acute alerting effects during daytime are still inconclusive. Hence, there is a need for more robust objective markers, such as EEG measures, to quantify the light-induced moderations in alertness. Existing studies fall short on reaching a consensus on the behavior and the meaning of different EEG band-width powers in the context of diurnal light exposure [6]. EEG frequencies are used as objective markers of people’s responses to various conditions. For example, alpha waves are traditionally associated with a more relaxed state, whereas theta power more for cognitive and memory-related processes. Similarly, beta power is often used as an indicator for higher-level processes such as attention and cognition. **Methods**: In this study, we aimed to systematically investigate the effect of light on various EEG-derived features, and used metameric lighting conditions to account for potential confounds due to visual processes. Metameric light in our context means a large contrast for ipRGCs while keeping the activation at the other retinal cells constant with similar illuminance and CCT levels [7]. The study used a within-subjects design in which participants (N = 22) were exposed to three different light conditions: dim light (<10 lx, 2.5 M-EDI), low-melanopic photoreception (212 lx, 55 M-EDI) and high-melanopic photoreception (211 lx, 175 M-EDI) for 30 min each with 30 min breaks of dim light in between, and with an hour of preparation and adaptation in dim light before the experimental sessions. **Results**: The data collection and preprocessing of the EEG data are finished, and data analysis is ongoing. We hypothesize that the high-melanopic condition will show significantly different spectral power in the alpha, delta and theta bandwidths in frontal, parietal and temporal electrodes than the dim light and low-melanopic photoreception condition. Multilevel analyses will be performed to test if—and which—spectral measures can serve as a sensitive measure to capture the NIF effects of light on the brain during daytime. Moreover, possible correlations between the EEG power densities and subjective assessments will be investigated and shared at SLTBR 2023. This research was performed within the European Training Network LIGHTCAP (project 860613) under the Marie Skłodowska-Curie actions framework H2020-MSCA-ITN-2019.

**Keywords:** metameric light; EEG; ipRGC; silent substitution; EEG frequencies; KSS

**References**

1. Provencio et al., 1998.
2. Kaida et al., 2007.
3. Phipps-Nelson et al., 2003.
4. Rüger et al., 2005.
5. Smolders and De Kort, 2014.
6. Lok et al., 2018.
7. Spitschan and Woelders, 2018.

### 2.21. Light, Activity and Sleep in My Daily Life: Usability and Feasibility of an Online Intervention in a Field Study

GerhardssonKiran MainiHassanMariamTornbergÅsa B.SchmidtSteven M.Lund University, Lund, Sweden

**Abstract: Background**: Research indicates that indoor lighting, exposure to daylight, physical activity and sleep interact to influence functioning, mood and daily rhythm. Hence, strategies are needed to support behaviour change among older adults who often spend more time at home after retirement. The study’s objective was to assess the usability and feasibility of a web-based intervention to encourage behaviour change related to light, outdoor activity, sleep, and self-managed modifications in the home. The nine-week intervention was delivered on a digital learning platform, including two physical group meetings. A test kit was included in the intervention material. The intervention comprised the following components: education, skills training (e.g., using the test kit and an inventory form), behavioural changes, homework, personal encouragement from the interventionist, and initial face-to-face contact with the interventionist. Grounded in the information–motivation–behavioural skills model, the intervention aimed to promote well-being (e.g., better mood and sleep) and improve lighting and darkness conditions at home. The technology acceptance model was used to evaluate the web-based intervention’s usability. **Methods**: Volunteers were recruited at senior citizen meeting points and throughout Lund Municipality. Eight healthy women aged 71–84 (*Mdn* = 75.5) living in one-person households participated. Data were collected before and after the intervention through observations of environmental features at home, accelerometry, interviews and questionnaires (an adapted version of Swedish Core Affect Scales consisting of two dimensions (pleasantness and activation (New General Self-Efficacy Scale, Short Computer Anxiety Scale, PROMIS 4-item Sleep Disturbance instrument, perceived indoor lighting quality consisting of two dimensions (strength and hedonic tone), and System Usability Scale). In the analysis of the accelerometry data, two participants’ data were excluded due to missing data and because medical surgery affected the behavioural patterns of one participant. **Results**: All participants completed the intervention. Time logged in varied between 25 min and 3 h (*M* = 1 h 50 min) per week. Seven participants’ system usability scores were between 90 and 100 (excellent) out of 100. Seven participants made three to nine changes to their lighting or darkness conditions: replaced bulbs with either three-step dimming or higher colour temperature LEDs (samples were included in the test kit), adjusted existing spotlights, installed luminaires, rearranged furniture or changed window treatments to allow more daylight to enter. When interviewed, participants reported overall high satisfaction with what they had learnt. Several participants were particularly satisfied with the modules’ targeting light. One suggestion to improve the online delivery was to enable participants to add text comments to the weekly evaluation form. Regarding secondary outcomes, preliminary analyses of accelerometry data showed that four of six participants had increased their physical activity, and five had increased their total sleep time. Four participants reported higher sleep disturbance total scores after the intervention. **Conclusions**: Only minor changes to the intervention are needed based on participants’ feedback. The web-based intervention can benefit older adults living in ordinary homes because of the relevant, easy-to-use content.

**Keywords**: intervention; behaviour; well-being; older adults

**Funding**: Lund University.

### 2.22. 40 Hz Masked Flickering Light as a Potential Treatment of Depression

SakalauskaiteLaura^1^DuboisJulie^2^LarsenMalina Ploug^2^PetersenPaul Michael^1^NguyenMai^3^MiskowiakKamilla^4^MartinyKlaus^2^1Technical University of Denmark, Department of Electrical and Photonics Engineering, Kongens Lyngby, Denmark2Mental Health Center Copenhagen, New Interventions in Depression Group (NID), Department of Clinical Medicine, Copenhagen, Denmark3OptoCeutics Aps, Copenhagen, Denmark4The Neurocognition and Emotion in Affective Disorders Center (NEAD), University of Copenhagen, Copenhagen, Denmark

**Abstract: Background**: Major depressive disorder (MDD) is a debilitating condition that affects more than 300 million people worldwide. Light therapy has been utilized and shown beneficial results in the treatment of both seasonal and non-seasonal depression. One-third of depression cases do not respond to traditional antidepressant medication and require more invasive approaches, such as ECT or TMS. Inspired by previous pre-clinical and clinical studies in Alzheimer’s disease, we are testing a novel brain stimulation system based on gamma brain wave activation with masked flickering light. Endogenous gamma brain waves (30–120 Hz) can be measured with EEG and are associated with higher cognitive functioning and memory, in addition to neuroprotective effects, such as recruitment of microglia, higher expression of BDNF, and reduced hippocampal and cortical atrophy as a result of gamma frequency stimulation. **Aim**: We want to test the antidepressant effects of 6-week everyday use of 40 Hz light therapy in patients with major depressive disorder. **Methods**: A prospective, double-blinded, placebo-controlled trial will be conducted to test the effects of this light stimulation paradigm. Patients will be allocated into two groups—active treatment with 40 Hz ISF (invisible spectral flicker) and placebo arm—using continuous non-flickering light matched in color temperature and brightness. The flickering light used in the study is masked (ISF) by alternating between the two different spectral combinations of white light. This makes the user experience more pleasant and implementable into a daily routine. To estimate the antidepressant effects of light therapy, the Hamilton Depression Rating Scale (HAM-D6) will be administered at all visits. The mean change in HAM-D6 between the two treatment groups is the primary endpoint. A neurocognitive battery, including both emotional and non-emotional cognition, will be included to test changes in cognition as well as test if an early shift in cognition predicts the overall treatment response. **Outlook**: Current treatments for depression lack effectiveness and are based on trial-and-error approaches. Reliable biomarkers and long-term treatment solutions are needed to manage the increasing prevalence of this debilitating disorder. The brain response to 40 Hz visual stimulation induces interesting phenomena that have been associated with neuroprotective changes in Alzheimer’s disease. We want to test this noninvasive 40 Hz light therapy as an adjunctive treatment for depression.

**Funding**: The devices used in the study are manufactured and supplied to the investigators by OptoCeutics, Aps.

### 2.23. Perils of the Nighttime: Late Timing of Behavior Increases the Likelihood of Physical and Mental Health Disorders across Chronotypes in 73,888 Community-Dwelling Adults

LokRenske^1^WeedLara^2^WinerJoseph^3^ZeitzerJamie^1^1Department of Psychiatry and Behavioral Sciences, Stanford University, Stanford, CA, USA2Department of Bioengineering, Stanford University, Stanford, CA, USA3Department of Neurology, Stanford University, Stanford, CA, USA

**Abstract: Introduction**: The central human circadian clock drives various 24 h behaviors, including sleep timing. Even though the biochemical underpinnings are not well understood, humans often express a preference for going to sleep later (‘owl”) or earlier (“lark”). While humans may prefer a specific time at which to sleep, life may interfere with such plans, causing people to go to sleep later or earlier than they might otherwise prefer. Both morningness–eveningness and going to sleep later and earlier have been associated with susceptibility to a variety of mental and physical disorders. This study examined the impact of morningness–eveningness, actual sleep timing, and the alignment between the two on various mental and physical health outcomes. **Methods**: We analyzed data from participants in the UK Biobank (*n* = 73,888), a community-dwelling sample of adults. Preference for morningness or eveningness was determined by answering the question “Do you consider yourself to be?”, which was parsed into three categories, morning, intermediate, or evening preference. The timing of behavior was derived from nonparametric analysis of accelerometry data (start time of least activity, L5) and was also parsed in three categories (early, intermediate, and late). Health status was derived from the International Classification of Diseases, Tenth Revision, Clinical Modification codes, and χ² tests, odds ratio test (cross-sectional), and survival analyses (Cox hazard ratio, longitudinal) were used to determine statistical significance. All statistical tests were corrected for common demographics, including self-reported sleep duration and variation in day-to-day sleep stability. **Results**: **Cross-sectional analysis**. Results indicated that having an evening preference and exhibiting late behavior increased the prevalence and likelihood of mental and physical health disorders, including generalized anxiety disorder, depression, metabolic disorder, diabetes, obesity, hypertension, circulatory disorder, digestive disorder, respiratory disorder, and all-cause cancer. Alignment between behavioral preference and timing was beneficial for morning types. Alignment was, however, detrimental for evening types, as going to sleep later was associated with worse mental and physical health than going to sleep early despite being an evening type. **Longitudinal analysis.** A morning preference with a late timing of behavior was only associated with de novo MBN, depression, hypertension, and circulatory disorder. In contrast, an evening preference with early timing of behavior was only associated with de novo MBN, with statistics not surviving multiple corrections for depression, GAD, metabolic disorder, diabetes, circulatory and respiratory disorders. Every included physical or mental health disorder followed a similar trend, in which later timing of behavior, irrespective of chronotype, was associated with an increased likelihood of developing these disorders. In contrast, an early timing of behavior was protective. Not all of these trends reached statistical significance due to the limited number of de novo cases. **Conclusions**: Both mental and physical health outcomes are worse in individuals who rise later. To age healthily, individuals should go to bed early, despite their chronobiological preferences.

### 2.24. Synchronisation between Menstrual Cycles and the Luminance and Gravimetric Cycles of the Moon

Helfrich-FörsterCharlotteJulius-Maximilians University of Würzburg, Würzburg, Germany

**Abstract:** Many species synchronise their reproductive behaviour with a particular phase of the lunar cycle to increase reproductive success. The human menstrual cycle also has a period close to that of the lunar cycle, but an influence of the moon on reproductive behaviour remains controversial. In a previous study, we showed that the menstrual cycles of younger women (age > 35 years) coincided at times with the lunar luminosity and/or gravimetric cycles. The data used in that study were mostly recorded before the year 2000. We repeated our study with the menstrual cycles of young women from the year 2000 onwards. We found that significant synchronisation with the lunar cycles had been lost throughout the years. However, significant synchronisation with the lunar cycles remained during the winter months when the Earth is closest to the Sun and the gravimetric forces of the Moon and Sun on the Earth add up. Combining the old and new data, we found that the temporary synchronisation with the luminosity cycles of the Moon was lost from 2010 onwards, coinciding with the time when high-energy blue LEDs replaced other light sources and light pollution on Earth increased significantly. We hypothesise that human reproductive behaviour used to be synchronised with the Moon, but that our modern lifestyle and increasing artificial night-time light have changed reproductive physiology. The biological relevance of the ancient synchronisation will be discussed.

### 2.25. The Circadian Immune System in Cancer

ScheiermannChristophUniversity of Geneva, Geneva, Switzerland

**Abstract:** The process of cancer immunosurveillance is a mechanism of tumour suppression that can protect the host from cancer development throughout its lifetime. It is now beginning to emerge that the effectiveness of cancer immunosurveillance fluctuates across the day. I will discuss data showing that the initial time of day of tumour engraftment dictates the ensuing tumour size. As a consequence, cancer immunotherapy is more effective when synchronized with immune cell function. These data indicate that the circadian rhythms of anti-tumour immune components can also be of therapeutic relevance.

### 2.26. Circadian Misalignment and Its Impact on Liver Metabolic Function and Inflammation

RayDavidBaxterMatthewCicconeNickUniversity of Oxford, Oxford, UK

**Abstract: Background and Aims**: Non-alcoholic fatty liver disease (NAFLD) is globally prevalent and confers a high risk of morbidity via progression to non-alcoholic steatohepatitis (NASH). The aberrant accumulation of lipid in hepatocytes is associated with insulin resistance and accelerated cardiovascular risk. Circadian disruption in mouse models contributes to the development of hepatic steatosis and inflammation, but evidence in humans is lacking and the mechanisms responsible are undefined. **Results**: Compared to day workers, irregular-shift workers were more likely to have NAFLD/NASH defined by high DSI (odds ratio (OR) 1.29 (95% CI 1.18–1.4)) after adjusting for all covariates excluding BMI, with some attenuation after additional adjustment for BMI (OR 1.12 (1.03–1.22)). Likelihood of DSI-defined NAFLD/NASH was also higher in permanent night-shift workers (OR 1.08 (0.9–1.29)) in the fully adjusted model. Compared to participants with intermediate chronotype, those with extreme late chronotype had a higher likelihood of DSI-defined NAFLD/NASH (OR 1.45 (1.34–1.56)) and a higher likelihood of NAFLD/NASH by ICD10 code (OR 1.23 (1.09–1.39)). A direct measure of liver fat content (liver PDFF) was elevated in irregular-shift workers, but not permanent night-shift workers. To examine the likely mechanisms in more detail, we performed a circadian misalignment protocol on mice fed a choline-deficient, high-fat diet. The misaligned group had no change in body weight, but had large livers, with increased triglyceride content, increased glucose content, and increased fibrosis. Analysis of RNA expression revealed upregulation of inflammatory and immune cell profiles. **Conclusions**: Irregular-shift work and chronotype are associated with NAFLD/NASH, suggesting circadian misalignment as an underlying mechanism. In a tightly controlled experimental setup, misalignment is sufficient to drive a deterioration in metabolic liver disease, with a marked change in immune cell signatures. These findings have implications for health interventions to mitigate the detrimental effect of shift work.

### 2.27. Melanopsin-Driven Pupil Responses and Vulnerability to Mania

SoehnerAdrianeKellerLaurenFray-WitzerMayaChanSimeyCaswellAllisonEliaNathanWescottDelaineyRoeckleinKathrynUniversity of Pittsburgh, Pittsburgh, PA, USA

**Abstract: Background**: Bipolar disorders (BDs) are impairing and costly psychiatric conditions that typically emerge between the ages of 18 and 22. Mania and hypomania (mania/hypomania) are defining features of BD, and subthreshold mania/hypomania symptoms are the most reliable clinical precursor to future syndromal mania/hypomania onset. However, the emergence of syndromal mania/hypomania remains incredibly challenging to predict, underscoring the need for objective biobehavioral risk markers for the condition. Circadian dysregulation is a chronic feature of BD, and could be driven by altered sensitivity of the circadian photoentrainment system. The post-illumination pupil response (PIPR) measures the responsivity of melanopsin-containing retinal ganglion cells and captures the sensitivity of the photoentrainment pathway projecting from the retina to the circadian clock. Here, we examined the extent to which altered responsivity of the circadian photoentrainment pathway, via the PIPR, was associated with lifetime mania/hypomania (vs. depression) vulnerability. **Methods**: This preliminary analysis included 21 participants aged 18–24 years (*M* = 22.50, *SD* = 1.72, 15 female) without a BD diagnosis but recruited across a range of low-to-high lifetime subthreshold mania symptoms (Mood Spectrum Measure—Lifetime Version; MOODS). The MOODS assesses the lifetime incidence of manic/hypomanic and depressive symptoms. During a 24 h lab visit, participants completed PIPR assessments in the morning (1–2 h after waking) and afternoon (6–7 h after waking). PIPR outcomes were based on the interval following a series of 1-second red or blue light pulses. PIPR values were calculated as the net difference between red and blue at three post-stimulus intervals: 6 seconds (PIPR 6), 10 to 30 seconds (PIPR 20), and 10to 40 seconds (PIPR 30). All analyses were adjusted for age, sex, and mood symptoms. Repeated-measures mixed-effects models assessed diurnal variation in PIPR outcomes. Robust regression models tested associations between PIPR and MOODS lifetime mania/hypomania and depression symptom outcomes (mania models adjusted for depression and vice versa). **Results**: Diurnal variation was not observed for net PIPR 6 (F_1,17_ = 1.52; *p* = 0.231) and net PIPR 30 F_1,17_ = 2.64, *p* = 0.123), but there was a nonsignificant trend toward lower net PIPR 20 values in the afternoon vs. morning (F_1,17_ = 3.92; *p* = 0.0641). Subsequent analyses used net PIPR values averaged across morning and afternoon assessments. Greater lifetime subthreshold mania symptoms were associated with higher net PIPR 20 (*b*_20_ = 0.482, *p* = 0.005) and net PIPR 30 (*b*_30_ = 0.499, *p* = 0.004), but not net PIPR 6 (*b*_6_ = 0.163, *p =* 0.474). Lifetime depression subthreshold—syndromal symptoms were not associated with any net PIPR values (*b_6_* = −0.262, *p* = 0.228; *b*_20_ = −0.351, *p* = 0.208; *b*_30_ = −0.348, *p* = 0.239). **Conclusions**: Elevated responsivity to entraining pulses of light were associated with greater lifetime subthreshold mania/hypomania symptoms in young adults independently of lifetime depressive symptoms. Thus, PIPR could represent a promising biobehavioral risk marker of future syndromal mania/hypomania. Data collection is ongoing in this longitudinal study.

**Keywords:** bipolar disorder; mRGCs; pupillometry

**Funding**: National Institute of Mental Health Grant R01MH124828.

### 2.28. Bright Beginnings—Artificial Dawn Light Intervention for the Long-Term Augmentation of Psychological Well-Being and Cognitive Functioning in Young Healthy Adults: A Proof-of-Concept Study

KormanMariaMaaravi-HessegRinatiaAriel University, Ari’el, Israel

**Abstract:** The current proof-of-concept, within-subject study tested the effects of a daily use of the artificial dawn bed-site lamp during the last 30 min of sleep (Lumie Zest, gradually achieving 500 lux at awakening) on markers of cognitive performance, affective control, and participation in everyday living activities parameters alongside circadian and sleep parameters, in 21 young healthy university students (14 women, age: 24.5 + 4.6). All participants completed a four-week in-home experimental protocol that included three phases. (1) Baseline—during the first week of the experiment, participants continued with their day-to-day routines. (2) Sleep hygiene—at the start of the second week, participants were individually instructed by an occupational therapist on how to keep a sleep-hygienic environment and were requested to lessen the intrusion of any outdoor or indoor artificial light into the bedroom to a minimum. (3) Starting from the third week, the artificial dawn (AD) intervention period included exposure to an individually timed AD bed-side lamp for 14 days. Participants could set up the AD lamp to the wake-up time of their choice. During the four weeks of the experimental period, participants were asked to wear an actigraph and to complete a sleep diary every morning within a 30 min interval after awakening. The sleep diary included complementary information about the sleep–wake timings and visual analogue scales for daily assessment of the levels of subjective pleasantness, alertness, and activation. During the test meetings (at the end of the each of the three phases) at the participants’ home, participants completed a computerized battery of cognitive tests using the Cambridge Neuropsychological Test Automated Battery (CANTAB) on an iPad and filled several questionnaires: the Oxford participation and activities questionnaire (Ox-PAQ) for self-reported assessment of participation and activity level in everyday living; the Dysexecutive questionnaire (DEX) for self-reported cognitive, emotional and behavioral symptoms of day-to-day activities; the ADHD Self Report Scale Symptom checklist questionnaire (ASRS-6) and the Depression, Anxiety and Stress—21-Item Scale (DASS-21). The rmANOVA analyses with the three time points as a within-subject variable confirmed that the AD lamp exposure led to significantly better scores in participation and cognitive and emotional well-being assessments using validated clinical questionnaires. Moreover, improvements in cognitive performance between the baseline and the postintervention time points were found, including significant improvements in both speed and accuracy of performance in emotion recognition, rapid visual information processing, stop signal task and paired associates learning CANTAB tasks. These results suggest that a multitude of cognitive abilities, including emotional, executive, and motor functions, improved over the AD intervention period. We also found statistically significant correlations between some cognitive–emotional measures and improvements in circadian rhythm stability. Data from the current study confirm that unobtrusive, in-home, and personalized regimens of dawn simulating light intervention and individually tailored adjustments to improve sleep hygiene benefit cognitive–emotional well-being of young adults.

### 2.29. Environmental Therapeutics: A Coaching Model for the Public

LokRenske^1^RaoJessica^2^TermanMichael^3^1Stanford University, Stanford, CA, USA2Center for Environmental Therapeutics, New York, NY, USA3Columbia University, New York, NY, USA

**Abstract:** The emergence of chronotherapeutics over the last decade—the fruition of successful randomized, controlled clinical trials—poses both a problem and opportunity for chronobiology laboratory investigators. The data are leading to a demand for expert services; however, most clinicians on the front lines have yet to master the diagnostic and clinical skills for successful implementation, and manufacturers and distributors of chronotherapeutic products publish content without clinical or academic credentials, which can mislead those attempting therapy on their own. Postgraduate trainees in laboratory programs have not encountered the variety of cases seeking individualized treatment. The situation is exacerbated by the need to coordinate chronotherapeutics with other modalities, such as cognitive behavioral therapy (CBT), and the wide range of pharmaceuticals patients are prescribed for sleep and mood disorders. The nonprofit Center for Environmental Therapeutics (CET) aims to close the gap in clinical coverage with therapeutic coaching by chronobiologists, guided by professionals who can offer practical knowledge at the interface of controlled trials and individualized problem-solving. **Referrals and screening**: Applicants are referred by clinicians who lack experience with chronotherapeutics or by self-referral from press coverage or introductory material on CET’s website. Current foci of the coaching are problems with sleep duration, continuity, or quality and light-responsive mood disorders. Various therapeutic modalities, such as CBT, may be combined with fundamental knowledge about the circadian clock and sleep. Screening includes medical history, past attempts with unsuccessful treatment, and an agreement to coordinate coaching with current psychotherapy or medical supervision. **Introductory session**: Virtual contact begins with a one-on-one coaching session after the candidate briefly summarizes the problem and can continue if the recommendations are acceptable. The coach provides a fundamental explanation of the biological factors underlying the behavioral issues and proposes a treatment plan based on timed daylight exposure, light therapy, schedule of feeding and fasting, exercise timing, and melatonin administration. **Monitoring and continuation**: If they agree to move forward, a second 30 min session is scheduled to assess initial indications of improvement, level of compliance, and regimen modification if indicated, after which limited follow-up through email is allowed. Progress is assessed primarily through self-report via email. Validated tools, including sleep diaries and sleep quality and mood questionnaires, are used as secondary outcomes. The coach’s time effort is carefully monitored to avoid extended interactions, with a maximum of 4 h per case. Further follow-ups can be requested. There is a nominal charge per contact session. **Case example**: Males aged 37 reported winter depression and a delayed sleep–wake cycle, untreated and general difficulty waking in time for work, with seasonal exacerbation. **Treatment plan**: morning bright-light therapy; reduced evening light exposure (fewer room light fixtures, blue-blocking glasses); morning exercise instead of sleeping; a consistent sleep hygiene routine; and low-dose slow-release melatonin 90 min before bed. **Results**: Improved mood and sleep quality with earlier sleep onset and waking.

### 2.30. The Effect of Blue-Light-Blocking Glasses on Depressive Symptoms in Women in the Early Postpartum Period—A Pilot Study

SochůrkováAnna^1^,^2^EvansováKatarína^1^,^3^ŠebelaAntonín^3^,^4^1Sleep and Chronobiology Research Centre, National Institute of Mental Health, Klecany, Czech Republic2Department of Psychology, Faculty of Arts, Charles University, Prague, Czech Republic3Department of Psychiatry and Medical Psychology, 3rd Faculty of Medicine, Charles University, Prague, Czech Republic4Center of Perinatal Mental Health, National Institute of Mental Health, Klecany, Czech Republic

**Abstract: Aims**: The aim was to investigate the effect of blue-light-blocking (BLB) glasses on sleep quality and depressive symptoms in six-week post-partum women. Blue light exposure affects circadian rhythms and nocturnal light exposure, which is common in postpartum mothers, worsens their condition and may lead to insomnia. BLB glasses are therefore a potential option for non-pharmacological prevention of postpartum depression. **Methods**: In a randomized, placebo-controlled trial (RCT), eligible respondents were between 6 weeks and 5 months postpartum. Participants were assigned BLB glasses or placebo (clear glasses) for 4 weeks, which they wore every 1.5–2 h before falling asleep and then every time they woke up during the night. The efficacy of the glasses was assessed by comparing the results from questionnaires before and at the end of the intervention. The Edinburgh Postnatal Depression Scale (EPDS) was used to assess symptoms of postnatal depression and the Pittsburgh Sleep Quality Index (PSQI) was used to measure sleep quality. **Results**: Ten women participated in the study and nine women were in the final group after data processing. Statistical analysis was performed by two-sample t-test and then we graphically evaluated the differences of the scores in both questionnaires for the experimental and placebo groups. For the EPDS variable, the t-test did not reach statistical significance t(−2.25) = 3, *p* = 0.055. For the PSQI variable, the t-test also failed to reach statistical significance t(−1.90) = 3, *p* = 0.077. Due to the low of N, we also performed a graphical representation of the differences for both questionnaires between the experimental and placebo groups (EPDS difference 5.75; PSQI difference 5.2), which showed us that the experimental group achieved larger differences than the placebo group in both questionnaires. **Conclusions**: Although the descriptive statistical analysis did not confirm the significance of our intervention, there was a trend present, and we expect statistical significance in the main study, where the target sample is 40 women.

**Keywords**: postpartum depression; sleep; nonpharmacological intervention; blue-light-blocking glasses

**Funding**: Technology Agency of the Czech Republic grant FW02020025 (Stable and mobile devices to support circadian synchronization, treatment and prevention of mental disorders through full-spectrum light phototherapy).

### 2.31. Impact of Daytime Napping on Circadian Markers, Cognition and Brain Integrity in the Aged

ReytMathilde^1^DeantoniMichele^1^BailletMarion^2^DourteMarine^1^DeHaanStella^1^HammadGregory^2^MutoVincenzo^1^SchmidtChristina^1^1Sleep and Chronobiology Laboratory, GIGA-CRC-IVI and PsyNCog Research Unit, Faculty of Psychology, University of Liège, Liège, Belgium2Sleep and Chronobiology Laboratory, GIGA-CRC-IVI, University of Liège, Liège, Belgium

**Abstract: Objectives**: Sleep and circadian rhythms can act as powerful modulators of human brain function. During ageing, cognitive decline goes along with altered sleep regulation. One visible manifestation of such alteration might be the increasing occurrence of chronic daytime napping while getting older. Here, we assessed the impact of napping on sleep propensity, cognition and its underlying structural and functional brain changes in healthy older adults. **Methods**: Fifty-six healthy older adults were prospectively recruited with respect to their napping habits (20 women/69 + −5.5 years, half nappers). All individuals underwent actimetry screening to objectify daytime rest frequency, timing and duration. They further underwent a 40 h multiple nap constant routine. During the protocol, salivary melatonin, subjective sleepiness, psychomotor vigilance and electrophysiologically derived sleep parameters over nap opportunities were assessed. Participants finally underwent functional and structural magnetic resonance imaging (MRI). During functional MRI, they performed a working memory task, allowing for the assessment of functional compensation with increasing working memory load. **Results**: Compared to non-nappers, nappers presented a reduced amplitude of circadian sleep propensity, characterized by higher sleep efficiencies during daytime sleep opportunities and lower sleep efficiency during nighttime sleep (interaction session*group: *p* < 0.05). Independent of nap group, actimetry-derived late daytime rest timing was associated circadian misalignment, as expressed by an increased phase angle of entrainment between dim-light melatonin onset and activity onset time (*p* < 0.05). Finally, compared to non-nappers, nappers presented increased working memory performance at high-load levels (interaction group*load: *p* < 0.05), the performance of which correlated with brain activation in the dorsolateral prefrontal cortex (pcorr < 0.05). **Conclusions**: Our results suggest altered circadian sleep regulation and associated reduced cognitive performance in healthy older nappers. Brain imaging data point toward altered functional compensation in nappers compared to non-nappers. These are in line with recent reports suggesting chronic and long daytime napping as a health risk factor in the aged, including for cognitive fitness.

**Acknowledgments**: This study was supported by the European Research Council (ERC, ERCStG-COGNAP) under the European Union’s Horizon 2020 research and innovation programme (grant agreement 757763). This study was also supported by the Fonds de la Recherche Scientifique—FNRS under grant T.0220.20. C.S., F.C. and G.V. are research associates and M.D. is a FRIA grantee of the Fonds de la Recherche Scientifique—FNRS, Belgium.

### 2.32. Step into the LightCafe: Predictors and Effects of Light Therapy for Mood Disorders in the LightCafe in Eindhoven

RopsLisetteDepartment Bipolar and Light Café, GGzE, Eindhoven, The Netherlands

**Abstract: Background**: Light therapy has been used since the 1980s to treat winter depression, although the dose, duration, and timing of treatment have differed. The remission rate of winter depression light therapy is high: 80–90% within 1–3 weeks. Light therapy has few side effects. In general, the symptoms diminish quickly and disappear completely after stopping the light therapy. Nevertheless, light therapy is still not a widely used therapy. In Eindhoven, we started a LightCafe, an attractive, friendly offer of light therapy in combination with lifestyle tools. It is a unique concept started in Eindhoven. I will talk about the unique concept and present two light therapy studies. Not long ago, light therapy bipolar disorders were contraindicated. The first study is an open trial of light therapy for depressive episodes in bipolar patients in autumn/winter using a Dutch protocol specific for patients with a bipolar disorder. In 2020, we wrote the Dutch light therapy protocol for patients with a bipolar disorder and it was added to the Dutch guideline. The second study is an RCT in which we compared the effectiveness of light therapy in spring/summer with autumn/winter. **Methods**: Data for the first study were collected for September–April 2017–2018 and September–April 2018–2019. The data for the second study (RCT) were collected from January 2020 until September 2022. The first study included 58 patients and the second study included 83 in summer and 121 in winter. Patients in both studies received light therapy for a minimum of 5 days and a maximum of 3 × 5 days; there was a follow-up measurement after two weeks. Outcomes were Quick Inventory of Depressive Symptomatology (QIDS) scores and side effects. The second study also had a follow-up at 6 and 12 weeks. **Results**: First study: QIDS scores were significantly lower at the last day of therapy (B = −6.00, *p* < 0.001) and 2 weeks after the end of treatment (B = −6.55, *p* < 0.001) compared with preintervention. Remission (QIDS ≤ 5) was reached in 55% of the treatments and response (50% symptom reduction) in 57% of the treatments. Side effects were mild; two hypomanic periods occurred. Second study: QIDS scores were significantly lower at the last day of therapy compared with pre-intervention both in winter as in summer, with an average drop of about 5 points on the scale. Although there was a greater effect at the last day of therapy and after 6 weeks, after 12 weeks, the difference almost disappeared. **Conclusions**: The Dutch light therapy protocol for patients with a bipolar disorder is a widely used protocol in treating seasonal bipolar depression. The effective is impressive and side effects are mild. Light therapy is effective both in winter and in summer in treating depression. The average decrease in QIDS score after light therapy is 5 points. Light therapy deserves a prominent place in the treatment because effects may be large and quick.

### 2.33. Metabolic Profiling of Shift-Work

SkeneDebraChronobiology, University of Surrey, Guildford, UK

**Abstract: Background**: Epidemiological studies have shown that shift-work is associated with increased risk of obesity, type 2 diabetes and metabolic syndrome. Shift-work involves sleep deprivation and misalignment of the circadian timing system. The mechanisms linking sleep restriction, circadian desynchrony and metabolic disease, however, are not fully understood. Using metabolic profiling (metabolomics) to track the effect of shift-work and consequent mistimed sleep, activity and feeding on metabolite rhythms and metabolic processing may help us to better understand the underlying mechanisms. **Methods**: We used two approaches to perform metabolic profiling in shift-work: (1) simulated shift-work in healthy volunteers in controlled laboratory conditions [8] and (2) real-life shift-workers on rotating shifts [9]. Simulated shift-work studies allow light–dark, feeding–fasting and sleep–wake timings to be precisely controlled and multiple time-series samples to be collected under constant routine or entrained conditions. **Results**: Our previous simulated shift-work study¹ analysing sequential plasma samples using targeted UPLC-MS/MS metabolomics showed that after 3 nights of working shifts, endogenous circadian rhythms of many plasma metabolites were misaligned from the central SCN circadian clock by ~12 h (internal desynchrony) and instead aligned with the food and sleep timing of the prior night’s shift schedule, likely reflecting the peripheral clocks’ response to mistimed behavioural cues. Since there is a limit to the number of sequential blood samples that can be collected in field studies of rotating shift-workers, we have recently tested an ambulatory device, URHYTHM, capable of sampling human interstitial fluid every 20 min for up to 27 h. Targeted UPLC-MS/MS metabolomics analysis of the interstitial fluid revealed daily metabolite rhythms that respond to meals and fasting. **Conclusions:** Misalignment between circulating plasma metabolite rhythms and central SCN circadian clock-driven rhythms (melatonin and cortisol) may underlie the adverse metabolic consequences of working shifts. Profiling metabolite rhythms in humans using metabolomics technology is a useful tool for tracking misalignment between the central and peripheral clocks in shift-work settings.

**Keywords**: shift-work; circadian rhythms; peripheral clocks; targeted metabolomics; circadian misalignment; humans

**References**

8. Skene, D.J.; Skornyakov, E.; Chowdhury, N.R.; Gajula, R.P.; Middleton, B.; Satterfield, B.C.; Porter, K.; Van Dongen, H.P.A.; Gaddameedhi, S. Separation of circadian- and behavior-driven metabolite rhythms in humans provides a window on peripheral oscillators and metabolism. *Proc. Natl. Acad. Sci. USA* **2018**, *115*, 7825–7830. https://doi.org/10.1073/pnas.1801183115
9. Harding, B.N.; Skene, D.J.; Espinosa, A.; Middleton, B.; Castaño-Vinyals, G.; Papantoniou, K.; Navarrete, J.M.; Such, P.; Torrejón, A.; Kogevinas, M.; et al. Metabolic profiling of night shift work—The HORMONIT study. *Chronobiol. Int.* **2022**, *39*, 1508–1516. https://doi.org/10.1080/07420528.2022.2131562.

### 2.34. Spectrawear, an Open-Source, Reasonably Priced, Wearable Dosimeter Suitable for Longitudinal Monitoring of Light Exposure in Alpha-Opic Units in Everyday Life

LucasRobertDidikogluAltugMohammadianNavidJohnsonSheenaTongerenMartie vanCassonAlexBrownTimothyUniversity of Manchester, Manchester, UK

**Abstract:** Experimental and interventional studies show that light can modulate numerous aspects of physiology and behaviour. Wearable light loggers capable of capturing personal light exposure information represent a critical underpinning technology in attempts to relate this basic biology to variations in human health and productivity in everyday life. Faced with the lack of a device capable of returning light exposure in alpha-opic irradiance (the recommended units for circadian and related non-image-forming light responses), we set out several years ago to develop our own. We set as our objective a device that would be sufficiently low in cost as to facilitate large-cohort studies and whose design we could release according to open-access principles, allowing others to manufacture their own devices and to ensure that users had full access to data produced. To this end, we developed a wrist-borne device, Spectrawear, based upon an available multichannel sensor. Once calibrated, we found that Spectrawear devices can return alpha-opic irradiance measures for a variety of indoor and outdoor light environments with within- and between-device error rates of <10%. We have continued to apply Spectrawear in combination with a smartphone-based online data collection method to relate longitudinal light exposure to sleep and sleepiness in a convenience sample of 59 UK adults. We found that Spectrawear was well tolerated by participants and was able to record light continuously for the 7-day study duration when charged overnight. The data provided by Spectrawear enabled us to describe several associations between light exposure and aspects of sleep and sleepiness in the real world. We are currently implementing improvements to the Spectrawear design based upon our experience with this first study and hope to have an updated device design, suitable for widespread adoption by researchers and light therapy practitioners, available later in the summer.

### 2.35. ENLIGHT Consensus Checklist and Guidelines for Reporting Laboratory Studies on the Non-Visual Effects of Light in Humans

SpitschanManuel^1^KervezeeLaura^2^LokRenske^3^McGlashanElise^4^NajjarRaymond P.^5^1Technical University of Munich, Munich, Germany2Leiden University Medical Center, Leiden, The Netherlands3Stanford University, Stanford, CA, USA4Monash University, Clayton VIC, Australia5National University of Singapore, Singapore

**Abstract: Background**: Beyond vision, light has wide-reaching effects on human health and well-being. However, there is no consensus on reporting light characteristics in studies investigating non-visual responses to light. This project aimed at developing a reporting checklist for laboratory-based investigations on the impact of light on non-visual physiology. **Methods**: To this end, a four-step modified Delphi process (three questionnaire-based feedback rounds and one face-to-face group discussion) involving international experts was conducted. **Results**: Across these four rounds, an initial list of 61 items related to reporting light-based interventions was condensed to a final checklist containing 25 items based upon consensus among experts (final *n* = 60). Nine of these items were determined to be necessary to report, regardless of the specific research question or context. A description of each item was provided in the accompanying guidelines. Most participants (92%) reported being satisfied or very satisfied with the consensus process, checklist, and guidelines. The ENLIGHT Checklist and Guidelines are the first consensus-based guidelines for documenting and reporting ocular light-based interventions for human studies. The implementation of the checklist and guidelines will enhance the impact of light-based research by ensuring comprehensive documentation and reproducibility and enabling data aggregation across studies.

### 2.36. Prediction of Retinal Irradiance across the Visual Field through Physically Realistic Spectral Rendering Incorporating a Parametric Head-Shape Model

NakadeUdaySpitschanManuelTechnical University of Munich, Munich, Germany

**Abstract: Background**: Light impacts human physiology and behaviour through the retinohypothalamic pathway, connecting the melanopsin-containing ipRGCs with the hypothalamus. In optical radiation metrology, the so-called non-visual effects of light arising from this pathway are typically quantified using corneal irradiance measurements, yielding measurements of the melanopic equivalent daylight illuminance (lux mEDI) at eye level. These and similar metrics assume a cosine-weighted integration of light from all directions. However, the human eye is shaded by facial features, including the nose, requiring the development of more complex spatial metrology for evaluating the non-visual effects of light. **Methods**: Here, we probed the impact of head shape on retinal irradiance using a physically realistic 3D spectral stimulation pipeline built on Mitsuba3. We stimulated the light reaching the retina from various directions across different head shapes using a low-dimensional head-shape model. **Results**: Our simulations produced a series of novel insights into how retinal irradiance varies with head-shape attributes, with the extent of the effective retinal stimulation field qualitatively matching typical psychophysically derived visual field boundaries. We found a dependence of simulated corneal irradiance on various parameters of the head-shape model. The results of these simulations will be presented. **Conclusions**: We find that we can simulate the spatial extent of retinal stimulation using spectral rendering tools. Our results can guide the optimization of light exposure given the observed patterns of stimulation and offer a method to evaluate the impact of the environmental α-opic radiance field on non-visual processes. In the next step, we will examine whether individual, idiosyncratic visual field boundaries match the predictions from individually measured 3D head models of real participants.

### 2.37. Selecting, Implementing and Evaluating Control and Placebo Conditions for Light Therapy and Light-Based Interventions: An α-Opic Framework

SpitschanManuelTechnical University of Munich, Munich, Germany

**Abstract: Background**: Light has a profound influence on human physiology, behaviour and cognition. Through a pathway connecting the fine layer of photosensitive cells in the eye, the retina, to various retinofugal targets, light affects multiple functions, including the circadian system and melatonin suppression. The use of light therapy, sometimes also called bright-light therapy, has been established as an effective method to mitigate and treat seasonal depression and other affective disorders. Research on the efficacy of light-based interventions typically compares some active light condition, e.g., bright light at a corneal illuminance of 10,000 lx, with a control or placebo condition designed or purported to be ineffective in stimulating the biological pathway underlying the positive treatment effects. To date, there is no guidance on selecting, implementing and evaluating appropriate control/placebo conditions. **Methods**: Here, we present a systematic framework for selecting, implementing and evaluating appropriate control/placebo conditions in studies using light or light exposure as the primary intervention grounded in α-opic optical radiation metrology. **Results**: We discuss various strategies from the literature for devising appropriate control/placebo conditions for light-based interventions, including variations in intensity (“dim light”), wavelength (“red light”), spectral tuning (“amber light”) or a combination of these (“dim red light”). We also discuss metameric light pairs, which (nominally) appear to an observer as identical light but stimulate the melanopsin-containing ipRGCs selectively, as a viable strategy for control/placebo conditions. The special cases of dynamic lighting interventions, simulated dusk/dawn interventions and studies with retinal patients lacking specific photoreceptors classes are discussed. **Conclusions**: We provide a practical guide for selecting, implementing and evaluating control and placebo conditions for light therapy and light-based interventions.

### 2.38. Daylight Quality: High-Transmittance Glass versus Low-Transmittance Glass—Effects on Daylight Quality, Health, Comfort and Energy Consumption

VolfCarlo^1^PetersenPaul Michael^2^ThorsethAnders^2^MartinyKlaus^1^,^3^VestergaardStefan^4^1Mental Health Centre Copenhagen, University Hospitals Copenhagen, Department of Clinical Medicine, University of Copenhagen, Copenhagen, Denmark2DTU Electro, Technical University of Denmark, Roskilde, Denmark3Mental Health Center Copenhagen, New Interventions in Depression Group (NID), Department of Clinical Medicine, Copenhagen, Denmark4Alfabo Public Housing, Kolding Aapark 8, Kolding, Denmark

**Abstract: Background**: This study investigated the health effects of two different architectural glass types used in architectural planning today: a high-transmittance two-layered low-iron glass and a three-layered low-energy glass with lower light transmittance. The study investigated how the different glass types affected daylight conditions in apartments and how they affected resident’s health and sleep, as well as the residents’ general satisfaction with the apartments. The study is the first large-scale study of its kind analyzing the effects of different architectural glass types on healthy individuals in the built environment. **Methods**: Out of a total of 72 apartments, we installed two-layered high-transmittance low-iron glass (LT: 0.82) in 36 apartments and three-layered low-energy glass with lower light transmittance (LT: 0.74) in 36 identical apartments. The study analyzed the indoor environmental quality (IEQ) in eight representative apartments before renovation and after renovation, respectively. Glass types differed significantly in measured light transmittance. **Results**: Results showed that two-layered low-iron glass transmitted 15% more visual light (380–750 nm) and 20% more light in the spectral range (460–480 nm), stimulating the ipRGCs and the circadian rhythm. Also, significant differences were observed in the active UVB spectrum (280–315 nm), stimulating the formation of vitamin D and also acting as a germicidal agent when it comes to viruses, such as COVID-19. While two-layered low-iron glass did transmit active UVB, three-layered low energy glass did not. Self-reported questionnaires on health and satisfaction were collected before and after the renovation. A total of 34 residents responded, with 17 residents from apartments with two-layered low-iron glass and 17 residents from apartments with low energy glass. During a 12-month study period, residents in apartments with three-layered low energy glass reported more difficulties sleeping (*p* = 0.05) and higher satisfaction with daylight (*p* = 0.03) and ventilation (*p* = 0.04). Residents in apartments with three-layered low-energy glass experienced fewer days with “too cold” indoor temperatures (*p* = 0.02) when compared to residents with two-layered low-iron glass. Results on energy consumption for heating showed that two-layered low-iron glass reduced the energy consumption by 11.0%, while three-layered low energy glass reduced energy consumption by 9.4% when compared to one year prior to the renovation. **Conclusions**: This study may contribute to a discussion about the relation between daylight quality, overall well-being and health, energy consumption and total economy/life cycle assessment of the built environment. The results of this study show several significant findings and suggest further research performed in randomized large-scale studies.

**Keywords**: daylight; glass quality; architecture; chronobiology; sleep; mental health; energy consumption

**Funding/Disclosures**: Elforsk 348-018, Green Power Denmark.

### 2.39. A Higher Illuminance Reduces Momentary Exhaustion in Exhausted Employees: Results from a Field Study

FrickSophiaSmoldersKarinMeijLeander van derDemeroutiEvangeliaKortYvonne deEindhoven University of Technology, Eindhoven, The Netherlands

**Abstract: Background**: Burnout numbers are increasing worldwide, suggesting that mild symptoms are experienced by countless others still at work. Although organizational psychology often investigates burnout in relation to the psychosocial work context (e.g., work demands and resources), lighting research indicates that aspects of the physical work environment, such as office lighting and access to daylight, may also have implications for burnout. In field and laboratory studies, brighter light has been demonstrated to enhance alertness and vitality, reduce fatigue, and improve positive mood. These effects seem to be closely related to the energetic (vitality, alertness, and fatigue) and motivational dimensions (affect) that define the burnout phenomenon. Luminous exposure—or the lack of it—may therefore impact momentary burnout-related experiences via the same mechanisms in non-clinical participants. Moreover, previous research suggests that alertness-enhancing effects of light might be particularly salient in persons that have already experienced resource loss (e.g., at night when participants are sleep-deprived) (Lok et al., 2018; Smolders and Kort, 2014) suggesting that those experiencing high levels of trait burnout (i.e., a state of resource loss) may demonstrate more pronounced alertness-enhancing effects. Nevertheless, little is currently known about the direct effect of illuminance on momentary experiences related to burnout in healthy participants and to what extent trait burnout determines these relationships. We aimed to investigate whether a higher illuminance (averaged over the last 60 min) decreases subsequent momentary experiences related to burnout and whether these relationships depend on trait levels of burnout. **Methods**: Fifty-nine healthy participants participated in a seven-day ecological momentary assessment study consisting of three parts: (1) a general questionnaire assessing the two trait burnout components (exhaustion and disengagement), (2) seven momentary assessments per day assessing three burnout-related experiences (exhaustion, boredom, and (a lack of) feeling positively challenged), and (3) person-worn sensors to quantify luminous exposure close to eye level throughout the waking day. Participants received notifications to complete momentary questionnaires at semi-random intervals throughout the waking day via their mobile device. Three-level linear mixed models were run to test associations between illuminance and subsequent burnout-related experiences. **Results**: The findings suggest that higher average illuminance over the last 60 min reduced subsequent experiences of momentary exhaustion. In contrast, illuminance was not significantly related to boredom or feeling positively challenged. Trait exhaustion moderated the relationship between illuminance and momentary exhaustion: only in persons that experienced average or above average levels of trait exhaustion, illuminance was related to subsequent levels of momentary exhaustion. **Conclusions**: The results of our study suggest that it is not just the psychosocial work context but also the physical environment that impacts burnout-related experiences. While adequate lighting is important for all employees, higher illuminance might be needed for employees suffering from burnout-related symptoms to alleviate their feelings of exhaustion. Increasing the intensity of office lighting may be an effective strategy to reduce experiences related to exhaustion in the moment, particularly among employees that experience symptoms regularly.

### 2.40. Does One Night of Short Sleep Matter? Meta-Analytic Evidence for Decreases in Alertness

WüstLarissa^1^,^2^CapdevilaNoëmi^1^,^2^LaneLina^1^,^2^ReichertCarolin F.^1^,^2^LasauskaiteRuta^1^,^2^1Centre for Chronobiology, Psychiatric Hospital of the University of Basel, Basel, Switzerland2Research Platform Molecular and Cognitive Neurosciences, University of Basel, Basel, Switzerland

**Abstract: Introduction**: Considerable research has addressed effects of acute and chronic sleep restriction on sleepiness and cognition. Research results are mixed when looking at the effects of only one single night of restricted sleep. The present meta-analysis addresses whether one night of restricted sleep impacts subjective sleepiness and cognitive functioning during the following day. **Methods**: A systematic literature search was conducted via Scopus, Web of Science, and PubMed in February 2023. Studies were included where (a) participants were healthy adults, (b) study protocols did not include shift work, (c) studies included control and sleep restriction conditions of at least 2 h difference, (d) subjective sleepiness and/or cognitive performance was assessed in the control condition and after one night of sleep restriction, and (e) sleep was undisrupted during study nights. The present abstract focuses on subjective sleepiness and simple reaction time tasks. Subjective sleepiness was assessed via the Karolinska Sleepiness Scale and the Stanford Sleepiness Scale, which are single-item scales with 9 or 7 points, respectively. Simple reaction time tasks, such as psychomotor vigilance or similar tasks, are used to assess sustained attention, which is seen as an objective indicator of alertness. In total, 33 studies were included for meta-analyses, representing data of 780 participants. Data were extracted from paper text, tables, graphs, or supplementary materials. For retrieval of data presented graphically, WebPlotDigitizer was used. Three separate meta-analyses were run for subjective sleepiness, reaction times, and lapses, i.e., non-responses within 500 ms, in the reaction time tasks using R and the meta package. **Results**: Sleep duration in the sleep restriction condition in included studies was between 3 and 6 h. We opted for common effects models. The meta-analysis of subjective sleepiness revealed that participants felt significantly sleepier after sleep restriction compared to controls (*k* = 19 studies, standardized mean difference (*SMD*) = 0.824, 95% CI = [0.685, 0.964], *z* = 11.61, *p* < 0.001). Regarding objective alertness, participants responded significantly more slowly after restricted sleep compared to controls (*k* = 25, *SMD* = 0.371, 95% CI = [0.236, 0.506], *z* = 5.38, *p* < 0.001). Additionally, the number of lapses in reaction time tasks was significantly higher after sleep restriction compared to controls (*k* = 14, *SMD* = 0.462, 95% CI = [0.318, 0.605], *z* = 6.29, *p* < 0.001). **Discussion**: Our meta-analyses show significant small to medium effects on subjective and objective alertness after only one night of sleep restriction. As reduced sleep for a single night seems to occur rather often in modern societies, this finding raises awareness of the consequential slower reaction times that may translate into increased risks in everyday situations (e.g., driving) and highlights the importance of disseminating this information through public health and safety campaigns.

### 2.41. The Moderating Effect of Antidepressive Agents on the Change in Sleep Quality and Sleep–Wake Pattern after Receiving Light Therapy for Depression

VeldeThomas van der^1^,^2^VisserEmma^3^OttenheijmEmmy^1^MaesJochem^1^SimonsClaudia^1^SchlangenLuc^3^SmoldersKarin^3^KortYvonne de^3^RopsLisette^1^MarcelisMachteld^1^,^2^1GGzE, Mental Healthcare Institution Eindhoven, Eindhoven, The Netherlands2Department of Psychiatry and Neuropsychology, School for Mental Health and Neuroscience (MHeNs), Maastricht University, Maastricht, The Netherlands3Human Technology Interaction Group, Department of Industrial Engineering and Innovation Sciences, Eindhoven University of Technology, Eindhoven, The Netherlands

**Abstract: Background**: Bright-light therapy (BLT) has been found effective in treating depression, as well as sleep problems and circadian rhythm disturbances in healthy individuals and those with depression. Antidepressive agents (AAs) are the first-line of treatment for depression, but can influence circadian rhythms, sleep quality and sleep-related disturbances unintendedly, leading to both positive and negative outcomes. Meta-analyses have demonstrated that the combination of BLT and AAs is more effective in reducing depressive symptoms than either therapy alone. However, the extent to which AA treatment interacts with BLT in relation to sleep outcomes remains poorly understood. **Methods**: Over a two-year period, we included 204 (120 female, 84 male) depressed outpatients who received BLT in a café-like setting. Patients received BLT at an intensity of 10,000 lux for a duration of 30 min per day over 5 consecutive days, for a minimum of one week and a maximum of three weeks. At intake, the use of antidepressants was recorded and coded as a binary variable (yes or no) independently of the dosage or type of medication. Out of the total participants, 95 were identified as AA users, while 109 were classified as non-AA users. Subjective sleep quality was assessed using the Pittsburg Sleep Quality Index (PSQI), administered at baseline and at 6- and 11-weeks post-treatment. Sleep diaries were used to collect data on the patients’ sleep–wake patterns. The diaries were administered retrospectively on the first and last day of each treatment week, inquiring about the sleep onset, sleep offset, sleep latency and duration of awakenings in the preceding weekend or workweek. Multilevel mixed-effects regression models were utilized to examine alterations in sleep quality from baseline to 6- and 11-week follow-ups, as well as variations in self-reported sleep patterns during the treatment phase. The moderating effect of AA use was studied by including the use of AAs as a fixed factor in the model. Age, sex, sleep medication use, morningness–eveningness, and baseline depression score were entered as covariates. **Results**: Significant improvements in sleep quality, as indicated by a reduction in PSQI-score, were revealed after BLT at the 6-week and 11-week follow-up assessments. Furthermore, preliminary analyses revealed a potential increase in sleep duration and a decrease in sleep latency during the first week of BLT, while midsleep timing appeared to be unaffected. Notably, there was no significant moderating effect of AA use on these changes in sleep quality, duration and timing. Ongoing analyses are being conducted to further investigate and confirm these findings. **Conclusions**: This study could highlight the potential of BLT to improve sleep quality, latency and duration for patients with depression both as a standalone treatment and in combination with AA. Nonetheless, it is important to acknowledge that the study lacked a control condition and the analyses remain unfinished. Based on current findings, the use of AA does not appear to have a significant impact on the effectiveness of BLT in improving sleep quality.

**Keywords**: antidepressive agents; sleep quality; sleep–wake pattern; treatment interaction; light therapy; depression

### 2.42. Photic Sneezing in Response to Naturalistic and Parametric Light Stimuli: A Protocol and Pilot Study (n = 1)

BickerstaffLucien^1^SpitschanManuel^2^1Max Planck Institute for Biological Cybernetics, Tübingen, Germany2Technical University of Munich, Munich, Germany

**Abstract: Background**: Photic sneezing is a widespread phenomenon characterised by sneezing in response to bright-light exposure (typically direct sunlight), affecting around 20% of the population. This photic sneeze reflex (PSR) has been documented for a long time, but is poorly understood. Our goals were to collect real-world data to understand the naturalistic light conditions eliciting photic sneezing, and to develop an indoor protocol to reliably induce the PSR in affected subjects using parametric stimuli, a key stepping stone for further research on this topic. **Methods**: This pilot study was carried out on one male adult affected by photic sneezing (*n* = 1). For characterisation of naturalistic light conditions eliciting photic sneezing, the subject wore an Actiwatch at torso level, measuring light levels during daytime, while light-induced sneezes were logged into a journal. To study photic sneezing in response to artificial stimuli, a bespoke setup including a multi-primary LED source and an integrating sphere was used to present a 30-second light stimulus to the subject while collecting pupillometry data with an eye-tracker. A 1 min darkness adaptation period was observed before showing the stimulus. The variables of interest were illuminance (0, 440, 1100, 4400 and 17,600 photopic lux, white light), and distance from the light source (2, 4 and 6 m). The cumulative effect of light stimuli was also assessed (over 30 min). **Results**: Real-world light exposure data recorded conjointly with logged photic sneeze events showed a significant increase in illuminance values around 2 minutes before the sneeze event, before going back down to pre-sneeze levels within 10 min after. At a sneeze event, illuminance is on average ten times bigger than the illuminance values five minutes before the sneeze event. Despite exposure to more than 150 stimuli, no sneeze could be induced in the subject using the experimental setup. A strong tickling sensation was consistently reported, which shows that sneezing was close, especially for high-illuminance settings and short sitting distances from the light source. **Conclusions**: Real-world light data confirmed that a sudden increase in environmental lighting conditions can induce photic sneezing. However, deeper analysis is clearly necessary, for example, on instances of illuminance increments not eliciting a photic sneeze. The experimental setup only elicited tickling sensations, but with further optimisation, it should in theory be capable of reliably inducing photic sneezes, thereby opening further mechanistic study of photic sneezing.

### 2.43. Quantifying Light Regularity: Novel Metrics and Their Relationship with Sleep Regularity in Adolescents

HandAnthony^1^StoneJulia^1^ShenLin^1^VetterCéline^2^CainSean^1^BeiBei^1^PhillipsAndrew^1^1Turner Institute for Brain and Mental Health, School of Psychological Sciences, Monash University, Clayton, VIC, Australia2Department of Integrative Physiology, University of Colorado Boulder, Boulder, CO, USA

**Abstract: Study Objectives**: Humans evolved under regular light–dark cycles, providing consistent daily time cues for the circadian system. The role of light exposure in regulating sleep has been extensively studied, but the impact of the regularity of light exposure on sleep and circadian rhythms has received less attention, mainly given the lack of available metrics that can quantify light exposure across days. We propose three novel metrics to quantify light regularity that will help to further understand the relationship between sleep regularity and circadian disruption and investigate their potential in adolescents. **Methods**: Daily sleep–wake and light patterns were measured using wrist actigraphy in 75 adolescents (54% male, 17.17 ± 0.83 years) over 2 weeks of school term and a subsequent 2-week vacation. The Sleep Regularity Index (SRI) and social jetlag were computed for each 2-week block. Light regularity was assessed using (1) variation in mean daily light timing (MLiT), which calculates the average clock time in which an individual’s light exposure is above a specified threshold; (2) variation in daily photoperiod, which examines the interval of time between the first and last epoch in which light levels rose above a specified threshold; and (3) the Light Regularity Index (LRI), which determines the probability of whether an individual is in the same state above or below a light threshold at any two time points 24 h apart. Associations between SRI and each light regularity metric were examined, and within-individual changes in metrics were examined between school and vacation. **Results**: Higher SRI was significantly associated with more regular LRI scores during both school and vacation, indicating that more regular sleep patterns were associated with more regular light patterns for this dimension of light regularity. The relationship between SRI and LRI was consistent across the transition from school (restricted sleep) to vacation (unrestricted sleep), indicating that the relationship is not simply driven by restrictions on sleep timing. This important finding suggests that the correlation is likely to generalize to other populations who experience variable degrees of restrictions on sleep timing, such as shift workers. There were no significant associations of SRI with variation in MLiT or daily photoperiod. The three metrics were not correlated with one another, indicating that each metric is likely measuring different aspects of the light stimulus. Compared to school term, all three light regularity metrics were less variable during the vacation. **Conclusions**: Light regularity is a multidimensional construct, which until now has not been formally defined. The circadian system expects regularity, and light is its primary input. Here, we propose three promising ideas for the measurement of regularity in light exposure that are germane to the circadian system. These metrics have potential applications to other populations, particularly those that experience irregular sleep patterns such as shift workers.

### 2.44. The Efficacy of a Transdiagnostic Sleep Intervention for Outpatients with Sleep Problems and Concurrent Bipolar Disorder, Major Depression, or Attention Deficit Disorder: A Randomized Controlled Trial

KraghMette^1^DyrbergHenny^1^PedersenPernille^2^KristiansenSanne Toft^3^MartinyKlaus^4^1Department of Affective Disorders, Aarhus University Hospital Psychiatry, Aarhus, Denmark2DEFACTUM, Central Denmark Region, Aarhus, Denmark3Research Unit for Nursing and Healthcare, Department of Public Health, Health, Aarhus University, Aarhus, Denmark4Mental Health Center Copenhagen, New Interventions in Depression Group (NID), Department of Clinical Medicine, Copenhagen, Denmark

**Abstract: Background**: Patients with mental disorders are more likely to suffer from sleep problems than the general population. Sleep problems can include insomnia, circadian rhythm disorders or hypersomnia. A transdiagnostic approach combining cognitive behavioral therapy for insomnia (CBT-I) with chronotherapeutic components such as light therapy and social rhythm therapy addressing a broad range of sleep problems has proven efficient in a few American studies. The aim of our study is to investigate the efficacy of this transdiagnostic sleep intervention delivered as six individual sessions provided by Danish health-care professionals. We hypothesize that the intervention will reduce the severity of insomnia and increase sleep quality compared to a control group receiving short sleep hygiene education across diagnostic entities. **Methods**: The study is a randomized controlled trial enrolling 88 outpatients with bipolar disorder, major depression, or attention deficit disorder suffering from sleep problems. Patients are allocated to either an intervention group receiving six sessions of transdiagnostic sleep treatment or a control group receiving sleep hygiene education at a single session. The six treatment sessions have the following content: assessment and introduction of a sleep diary, stimulus control therapy, sleep restriction, light exposure/therapy, education on sleep and circadian rhythm, cognitive behavioral therapy, relaxation techniques and relapse prevention. Assessments are carried out at baseline, at week 2 and after 6 weeks; actigraphy is performed continuously throughout the 6-week study period. Primary outcomes are changes in the subjective appraisal of sleep quality and the severity of the insomnia problem. Secondary outcomes are changes in sleep efficiency, sleep latency and wakinge after sleep onset, quality of life, personal recovery, work ability and consumption of sleep medication. **Results**: The study was initiated in June 2022, and we have included over a third of patients in the study. The inclusion period will continue till mid-2024. **Conclusions**: The results could contribute to the development and implementation of nonpharmacological treatment options for patients with mental illness and sleep problems.

**Funding**: This study is supported by the Public Health Foundation, Public Health in the Centre, Jascha Foundation and Director Werner Richter and Wife’s Legacy.

### 2.45. Pilot Field Study to Investigate the Relationships between Social Jetlag, Food Intake and Glucose Patterns in Night Workers

LuxwoldaMichelle^1^GordijnMarijke^1^HutRoelof^2^1Chrono@Work, Groningen, The Netherlands2University of Groningen, Groningen, The Netherlands

**Abstract: Background**: Long term health risks for shift workers including cardiovascular disease (CVD), increased obesity and diabetes are likely in part related to eating at night. Nocturnal insulin resistance causes higher glucose levels in response to food intake. Regularly high glucose levels result in the formation of higher levels of advanced glycation end products (AGEs). Shift workers show a faster accumulation of AGEs with ageing compared to day workers, which increases their risk of developing CVD. Disturbed sleep in shift workers also forms a risk for developing metabolic disturbances leading to a higher risk of CVD. Social jetlag, a measure of chronobiological disturbance, is large in shift workers. This pilot field study has two objectives. The first is to describe the effect of time of day between food intake and glucose patterns in a field study of night workers. The second objective is to see if social jetlag relates to 24 h glucose patterns in night workers. **Methods**: During a 14-day field study, continuous blood glucose levels were measured by the FreeStyle^®^ Libre™Pro glucose monitoring system in 12 healthy shift workers. The four conditions consisted of one night and one day shift, which included time restricted eating (TRE) combined with a high-protein (HP) meal during the shift. Another night and day shift were without regulations on food intake. Social jetlag was measured by the MCTQ shift. **Results**: Over the course of 2 h after an HP meal timing, the TRE + HP condition showed a significant reduction in glucose levels compared to glucose levels during regular food intake, independently of time of day. The average tAUC from glucose patterns over 2 h was smaller in night shifts with TRE+HP (9.5 ± 0.5 mmol/L) than within night shifts without regulations (11.3 ± 0.5 mmol/L). The average tAUC from glucose patterns over 2 h was also smaller in day shifts with TRE+HP (9.4 ± 0.2 mmol/L) than within day shifts without food regulations (11.1 ± 0.3 mmol/L). Two-way ANOVA indicated a significant overall effect of the TRE + HP meal on the tAUC of glucose patterns over 2 h (F_(1,9)_ = 23.539, *p* = <0.001), with an effect size of 0.438, but no effect of time of day. This means that applying a TRE + HP meal compared to no regulations on food lead to a significant change in glucose patterns over the 2 h after both midnight and noon (after timing of the HP meal consumption). No significant relationship was shown between social jetlag and mean glucose patterns over 24 h. **Conclusions**: We have shown in shift workers that time restricted eating combined with a high-protein meal causes significantly lower postprandial glucose levels compared to regular food intake, independently of the time of day. Glucose patterns in night workers have a more direct dependence on food intake and are probably less related to individual characteristics in social jetlag.

**Keywords:** shift work; diabetes; cardiovascular disease; social jetlag; time restricted eating; protein

**Funding**: FOOD2020 project as part of the INTERREG V-A Programme Germany–The Netherlands.

### 2.46. Real-World Variation in Pupil Size during Activities of Daily Living across the Lifespan

LazarRafael^1^SpitschanManuel^2^1University of Basel, Basel, Switzerlands2Technical University of Munich, Munich, Germany

**Abstract:** Human visual perception begins with light entering the eyes and subsequently stimulating the receptors in the retina. The pupil has the crucial task of regulating the amount of incident light by dilating or constricting (between approx. 8 and 2 mm in diameter, with the average pupil diameter decreasing with age). Pupil size not only influences the quality of the retinal image but also modulates non-visual effects of light (e.g., melatonin suppression by light). In controlled laboratory experiments, it has been established that steady-state pupil diameter is primarily influenced by the activity of the intrinsically photosensitive retinal ganglion cells (ipRGCs) expressing the short-wavelength-sensitive photopigment melanopsin (λmax = 480 nm) under photopic conditions. However, there is a lack of investigations on pupil size regulation in dynamic, real-life lighting conditions across the lifespan. **Methods**: Informed by previous laboratory studies, we set out to test the following confirmatory hypotheses under everyday lighting conditions: (a) melanopic equivalent daylight illuminance (melanopic EDI) predicts steady-state pupil size, (b) melanopic EDI predicts steady-state pupil size better than photopic illuminance, and (c) pupil size decreases as a function of age. We integrated a wearable infrared video-based eye tracker (Pupil Labs GmbH) with a small-scale, calibrated research-grade spectroradiometer (Ocean Insight). Both devices were attached to a bespoke, 3D-printed, and adjustable head mount and connected to a miniature, battery-driven control computer (Raspberry Pi), enabling simultaneous sampling of pupil size and spectral irradiance in the near-corneal plane at 10-second intervals. We measured the natural variation in pupil size during a 60 min protocol during which healthy participants from a broad age range (*n* = 80, age: 18–87 years, 51% female) engaged in various activities of daily living indoors and outdoors under naturally varying light conditions. **Results**: Our data demonstrate that under photopic real-world conditions, pupil size is strongly determined by light intensity and declines with higher age, yielding steeper age effect slopes in dimmer light conditions. Bayesian statistical analysis shows decisive evidence for the superiority of melanopic EDI compared to photopic illuminance in predicting pupil size. In line with the sluggish properties of melanopsin signalling, our exploratory analysis revealed that averaging the preceding 60 seconds of melanopic EDI values further improved pupil size prediction over using simultaneous melanopic EDI samples. Our real-world dataset yields no evidence for sex, iris colour or reported caffeine consumption significantly affecting steady-state pupil size. **Conclusions**: Our study sheds light on the factors influencing human pupillary physiology in real-life conditions, confirming findings from prior laboratory experiments. Taken together, the data provide strong evidence for considering age in personalized lighting solutions and extending the use of melanopsin-weighted light measures to evaluate real-world light conditions.

### 2.47. Physiologically Relevant Multi-Modal Characterization of Natural Scenes across Time, Space and Spectrum

TabandehNiloufar^1^HahnAlexander^1^SpitschanManuel^2^1Max Planck Institute for Biological Cybernetics, Tübingen, Germany2Technical University of Munich, Munich, Germany

**Abstract: Background**: Light exposure significantly impacts various aspects of human physiology, including cognition and the circadian clock. Light is detected by the retinal photoreceptors, cones and rods, which enable us to see the world, and the ipRGCs, which signal the intensity of ambient illumination. The world around us is complex in its visual attributes, differing in spectrum, intensity, color, spatial articulation and temporal properties. To comprehensively characterize the world around us vis-à-vis its visual and non-visual physiologically relevant properties, we started an image-capture campaign to map out the spectral, spatial and temporal properties of indoor and outdoor natural scenes across a wide range of geographical and seasonal contexts. **Methods**: We developed a multi-modal, minimal-parallax image capture setup for capturing natural scenes comprehensively. We are collecting radiance images using an α-opic imaging radiometer (40° × 48° FOV, 2712 × 3388 px^2^), spectral irradiance measurements using a high-resolution spectroradiometer (380–780 nm, 1 nm resolution), illuminance and colorimetric (xy) measurements (~8 Hz), depth information using a depth camera (15 fps), and uncalibrated wide-field RGB videos (60 fps). All measurement devices controlled are integrated into a compact box (42 × 32 × 27 cm^3^), which can be mounted on a tripod and controlled by a laptop. Power is supplied through external batteries, making the system suitable for indoor and outdoor use. In addition to the primary light-based data, we comprehensively describe the scenes using a novel metadata schema containing location, weather and other information. We collect natural scene data using two distinct protocols. First, we collect data from the same scene over a day in a time-lapse protocol. Second, in a trajectory protocol, we are simulating a hypothetical trajectory of an individual throughout the day, thereby capturing plausible natural scenes that people might be exposed to throughout the day. **Results**: We have finished piloting and technical checks and are now collecting time-lapse data across various natural scenes. We have subjected our image-capture setup to a rigorous cross-validation check, indicating high between-device agreement for related quantities. Our setup has been proven portable for field measurements, facilitating the development of a geographically diverse dataset, with planned measurements taking place in Germany, Czech Republic, France and Canada in the first wave. **Conclusions**: Our data collection campaign will yield a highly significant, geographically spread, worldwide database for developing novel approaches to understanding illumination and scenes in the real world. All data captured with our system are converted to physiologically relevant cone-, rod- and melanopsin-based quantities and metrics to relate our data to lighting recommendations and guidelines supporting human health. The data will be carefully documented and published openly, creating the first open-access reference resource for natural scenes’ spectral, spatial and temporal properties, indoors and outdoors.

### 2.48. Living without Light–Dark Cycle: The Consequences on the Sleep–Wake Cycle—Deep Time Mission

GabelVirginie^1^DelaunayPierre-Louis^1^HingrandCorentin^1^CotChristian^2^BesnardStéphane^3^MauvieuxBenoit^1^1University of Normandie, Inserm U1075 COMETE, Caen, France2Human Adaptation Institute, Paris, France3Vertex Laboratory, UR 7480, University of Normandie, Caen, France

**Abstract: Introduction**: In the 1960s, multiple solitary isolation experiments achieved by Michel Siffre and his teams highlighted the endogenous rhythm of the circadian clock, estimated to be around 24.5 h in the absence of any time giver. In our study, the objectives were to determine whether a group of people living out of time will exhibit the same endogenous rhythm as previously observed and whether their activity ratio will be modulated. **Methods**: Through the Human Adaptation Institute, 14 individuals (7 ♀ and 7 ♂) isolated themselves from any time giver in a cave (Lombrives, France) for a period of 40 days between March and April 2021. Their sleep–wake cycle was monitored by wrist actimetry (MotionWatch R, CamNTech) for 14 days outside the cave (“Pre”), during the 40 days in the cave (“Per”) and 14 days after exiting the cave (“Post”). A sleep diary was completed each cycle and core body temperature (CBT) was measured every five cycles for several hours. **Results**: Within the 40 days, we recorded 24 to 31 sleep–wake cycles per subject (mean ± SD: 29.25 ± 2.56) with a mean duration of 31.7 h ± 8.0 per cycle. The ratio “time awake” to “time asleep” stayed stable across all conditions (“Pre”: 64.63% ± 5.9, “Per”: 63.64% ± 8.78 and “Post”: 62.36% ± 8.27, *p* = 0.201). Results of CBT reveal a desynchrony between their sleep–wake cycle and their circadian rhythm on the first half of the experiment, followed by a synchrony until the end of the experiment. Their temperature’s periods were also longer than previously observed (mean ± SD: 30.25 h ± 6.14). **Conclusions**: We showed that people spontaneously extended their sleep–wake cycle over the course of the 40 days, and not necessarily following their circadian rhythm. Interestingly, the ratio rest/activity stayed the same in and outside the cave, even though their internal rhythms tend to be longer than what were previously shown by other studies, leaving a field open to other hypotheses concerning human adaptations.

### 2.49. Chronobiotic Use of Melatonin Improves DaT Binding in iRBD

KunzDieter^1^,^2^ZeeuwJan de^1^StotzSophia^1^,^2^PlotkinMichail^3^BesFrederik^1^,^2^1Sleep Research and Clinical Chronobiology, Institute of Physiology, Charité–Universitätsmedizin, Berlin, Germany2Clinic for Sleep and Chronomedicine, St. Hedwig-Hospital, Berlin, Germany3Institute of Nuclear Medicine, Vivantes Clinics Berlin, Berlin, Germany

**Abstract: Introduction**: Isolated REM-sleep behavior disorder (iRBD) is recognized as a prodromal state of clinical α-synucleinopathies such as Lewy-body dementia and Parkinson’s disease. A pathophysiologic hallmark of α-synucleinopathies is nigrostriatal dopaminergic impairment, with dopamine transporter (DaT)-SPECT imaging considered the best available prognostic and monitoring marker. DaT binding is reported to decrease with healthy aging by 4–10% per decade, being accelerated to 4–12% per year in patients with iRBD. We have introduced melatonin as a treatment option for iRBD. The aim of the study was to evaluate effects of melatonin on DaT-SPECT imaging in patients with iRBD. **Methods**: In a prospective, longitudinal, observational, single-center study until December 2022, we performed at least two DaT-SPECTs in 78 patients with iRBD being treated with melatonin as a chronobiotic (i.e., administration always at the same clock time; 10–11 p.m.—corrected for chronotype); 23 patients were excluded mainly due to change of psychotropic drugs known to influence DaT. **Results**: After a mean follow-up of 3.3 years, only 12 of 55 patients (7 female; mean age 70 ± 7 yrs) showed specific binding ratios (SBRs) in most affected region (MAR, predominantly right posterior putamen), comparable to usually reported declines with iRBD. In contrast, seven had declined SBR at a rate comparable to healthy aging, while 36 had actually improved SBR. Improvement after one year (SBR of MAR; F_(1,25)_ = 20.874; *p* > 0.001) and two years was significant (F_(1,21)_ = 10.083; *p* = 0.005). After four years, more than half of the patients showed a higher SBR than at baseline (20 vs. 16 patients), though this was not significant. In sum, 31/55 of our patients at baseline met established criteria for an advanced state. Instead of the expected 10–19 patients converting to clinical α-synucleinopathy (*n* = 31, FU-mean 3.1 yrs), only three patients in our cohort had converted by the end of the observation period. **Conclusions**: To the best of our knowledge, the present data give first evidence for a consistent increase in DaT-binding ratios in nigrostriatum over time in a cohort of patients with iRBD. In addition, the low conversion rate reported here and a previously reported persisting effect of melatonin on RBD symptoms suggest that melatonin, when used as a chronobiotic, may have a disease-modifying effect in prodromal α-synucleinopathies.

### 2.50. Seasonality of Human Sleep: PSG in Urban Neuropsychiatric Patients

WeihrichKaty Sarah^1^BesFrederik^1^,^2^ZeeuwJan de^1^,^2^HaberechtMartin^1^KunzDieter^1^,^2^1Clinic for Sleep and Chronomedicine, St. Hedwig-Hospital, Berlin, Germany2Sleep Research and Clinical Chronobiology, Institute of Physiology, Charité–Universitätsmedizin Berlin, Germany

**Abstract: Background**: Light is considered the strongest zeitgeber for the circadian pacemaker. Photoperiodic responsiveness in humans has been shown in experimental studies, including seasonal changes in hormones, physiology, and behavior, such as sleep length. On the other hand, simulating near-natural urban light conditions, effects of light were much less pronounced or not demonstrable. Thus, the effect of environmental light exposure on human sleep, especially in humans living in urban environments, is thought to be suppressed by access to artificial light sources and exposure to light pollution. Putative seasonal changes of the circadian processes underlying sleep in humans remain disputed. Recently, we reported significant variations in human sleep architecture over one year. The present study aimed to replicate these results and explore possible underlying mechanisms. **Methods**: PSG data were obtained from 372 neuro/psychiatric patients (49 ± 17 years, 54% female, without psychotropic medication) visiting our sleep-laboratory November 2017 till February 2020. Scheduling of the patients’ sleep was adjusted to their usual routine, including timing, but alarm clocks were not allowed. For analysis, a linear mixed-effect model was applied. For visualization, a 90-day moving average was used. Mean outside temperature of the preceding 14 days and the preceding day’s sunlight duration and photoperiod were correlated with raw sleep parameters and plotted against the moving averages. **Results**: Significant results for effect of month: TST (total sleep time, *F*_11,360_ = 2.54, *p* = 0.004), REM sleep (*F*_11,360_ = 2.94, *p* = 0.001), SWS (slow-wave sleep, *F*_11,360_ = 3.10, *p* = 0.001). Post hoc analyses showed strong significant differences: TST ~50 min (*t*_71_ = 3.46, *p* < 0.001, *d* = 0.81) and REM sleep ~25 min (*t*_71_ = 3.63, *p* < 0.001, *d* = 0.85) higher in December than June; SWS ~32 min (*t*_63_ = 4.00, *p* < 0.001, *d* = 1.04) lower in September than February. The 90-day moving average showed peak/nadir differences (Δ): ΔTST_2018/2019_ = 52/57 min; ΔREM_2018/2019_ = 23/22 min; ΔSWS_2018/2019_ = 23/29 min. While TST and REM sleep displayed similar patterns between years, the time courses showed differences. Declines in TST ran parallel ~2 months apart over 5-month periods and reached nadirs around late August in 2018 and early June in 2019. REM sleep displayed a similar pattern. Time course of SWS remained strikingly similar between years, continuously decreasing as soon as the photoperiod became longer than ~12 h (mid-March) and increasing again when the photoperiod became shorter than ~12 h (start-October). Significant correlations between sleep parameters and outside factors were negative (TST/temperature: *rs*_370_ = −0.24, *p* < 0.001; REM/temperature: *rs*_370_ = −0.22, *p* < 0.001; SWS/temperature: *rs*_370_ = −0.16, *p* = 0.001; TST/photoperiod: *rs*_370_ = −0.20, *p* < 0.001; REM/photoperiod: *rs*_370_ = −0.22, *p* < 0.001; TST/sunshine: *rs*_370_ = −0.13, *p* = 0.005). **Conclusions**: The previously reported pattern of prolonged TST/REM sleep in winter and shorter SWS in autumn were replicated by the present data. Findings still need to be confirmed in a healthy population. Recommendations could be made for sleep-disturbed patients to consider seasonal adjustments to sleep habits. Nevertheless, the magnitude of variation in sleep architecture throughout the years, even in an urban population with low natural light exposure and high light pollution, is impressive. Furthermore, the observed variation between years might be explained by weather factors. The 2-month shift between the declines in TST/REM sleep might be related to freezing temperatures. In 2018, Berlin reached daily average temperatures below freezing until mid-March, while in 2019 this period ended in early February. Steady declines in TST/REM sleep started 2 and 4 weeks after that, respectively. The similar decline in SWS in both years might underlie different mechanisms, independently of weather factors.

**Keywords**: seasonality; human; sleep; PSG; REM; SWS

### 2.51. Sensitivity and Variations in Self-Reported Sleepiness and Skin Temperature Field Metrics Following Chronic Sleep Restriction

VerhoefVaida^1^SmoldersKarin^1^PeetersGeert^2^OvereemSebastiaan^1^KortYvonne de^1^1Eindhoven University of Technology, Eindhoven, The Netherlands2Kempenhaeghe Sleep Center, Heeze, The Netherlands

**Abstract: Background**: Daytime sleepiness is a common yet often unaddressed symptom of individuals who experience chronic sleep disruptions or disorders. This difficulty in recognition has been related, among others, with methodological issues. Indeed, although many metrics of sleepiness exist, questions remain on their sensitivity and specificity. Whether in clinical or non-clinical research, literature shows inconsistencies between subjective measures (self-reports) and regarding correlations with objective measures (physiological and behavioural). The recognition of sleepiness is furthermore complicated by the overlap with fatigue: another prevalent yet differently addressed complaint. As such, we aimed to investigate the sensitivity of various common metrics of sleepiness to chronic sleep restriction, while also examining the relation between those metrics and measures of fatigue. **Methods**: To address these research aims, we performed a randomized cross-over study among 20 healthy participants (17 collected to date, 19–32 years old, 7 females and 10 males, PSQI ≤ 5, intermediate chronotype). We instructed participants to follow a sleep restriction paradigm with a baseline week followed by two sleep conditions (restricted 4 h sleep/night vs. normal 7–9 h sleep/night). The two sleep conditions were counterbalanced and lasted three consecutive nights each, with a four-night washout period in between. Sleep diaries and actimetry data were collected throughout the study. Apart from other physiological and behavioural metrics acquired in laboratory conditions on day three of each experimental session (not considered here), field measures were performed. On days following each sleep condition, an experience sampling method was used to acquire repetitive momentary self-reports of sleepiness (Karolinska sleepiness scale, Stanford sleepiness scale) and fatigue (through a visual analogue scale) with 10 semi-random prompts per day. Daily measures of both sleepiness (Epworth Sleepiness Scale) and fatigue (daily adapted Patient-Reported Outcomes Measurement Information System, PROMIS) were acquired at the end of each experiment day. Moreover, participants’ skin temperature (distal and proximal) and light exposure were monitored continuously during both sleep conditions with wearable sensors. **Results**: In order to investigate the cumulative effects of sleep restriction and the importance of time of day in self-reports and skin temperature metrics, we performed multilevel analyses. For each outcome measure, data were analysed through iteratively developed multilevel models, evaluated by model fit and significance of factors and covariates. Each model included fixed factors (sleep condition, day), random factors (participant number), relevant covariates (e.g., time of day), and potential random slopes to assess individual variations in the predictive strength of the factors and/or covariates. In addition, multilevel analyses were conducted to correlate sleepiness metrics with metrics of fatigue. Preliminary results of these analyses will be presented and discussed at the conference. **Conclusions**: Insights from the present study will enable us to reflect on the sensitivity and the reliability of field measures of sleepiness. Moreover, this acquired knowledge will guide future monitoring methods amongst patients diagnosed with sleep disorder(s) who experience excessive daytime sleepiness.

**Keywords**: sleepiness; fatigue; skin temperature; sleep restriction; homeostatic sleep pressure; circadian rhythm

**Funding**: This research is part of the European Training Network LIGHTCAP (project 860613) supported by the Marie Skłodowska-Curie actions framework H2020-MSCA-ITN-2019.

### 2.52. Modulatory Effects of Melanopic Irradiance on Executive Brain Activity in Late Teenagers and Young Adults

SharifpourRoya^1^CampbellIslay^1^BeckersElise^1^BaldaFermin^1^PaparellaIlenia^1^BergerAlexandre^2^KoshmanovaEkaterina^1^MortazaviNasrin^1^SherifSiya^1^LamalleLaurent^1^PhilipsChristophe^1^VandewalleGilles^1^1Sleep and Chronobiology Laboratory, GIGA-CRC-IVI, University of Liège, Liège, Belgium2Université Catholique de Louvain, Louvain, Belgium

**Abstract: Background**: The recently discovered blue-light-sensitive intrinsically photosensitive retinal ganglion cells, i.e., ipRGCs, are established as the main drivers of non-image-forming (NIF) effects of light. Blue light is considered to stimulate cognitive functions through widespread direct and indirect projections of ipRGCs to the brain. However, a precise circuitry of the NIF effects of light on cognition is still lacking. This study aimed to isolate the brain correlates of the impact of light irradiance level, as indexed through melanopic EDI lux, on an auditory working memory task and to assess whether this impact varied from late adolescence to early adulthood. **Methods**: Thirty-five healthy participants (15–30 years: 22.5 ± 4.0 years, 62.8% female) were included in a 7-Tesla functional magnetic resonance imaging (fMRI) study. The participants followed a loose sleep–wake schedule (±1 h; verified with actigraphy) for 7 days prior to the functional MRI session. While in the scanner, participants completed an auditory N-back task, with two levels of difficulty (0-back: N0 and 2-back: N2), under five different light conditions including darkness (~0 mel EDI), low monochromatic light (~0.2 mel EDI lux; 589 nm), and a polychromatic blue-enriched light at three irradiance levels (37, 92 and 190 mel EDI lux; 6500 K). Conventional fMRI analysis sought an impact of irradiance level on the N2–N0 contrast meant to isolate executive brain responses. **Results**: Preliminary results suggest that the light melanopic irradiance increases the activity of some cortical and subcortical brain regions involved in the ongoing executive processes including the right hippocampus, left superior frontal gyrus, left thalamus and left amygdala (*p*-value < 0.001). Further preliminary results suggest that this modulatory effect was negatively correlated with age: younger participants showed a higher impact of increased melanopic irradiance. **Conclusions**: These results support the fact that executive functions can be modulated by light irradiance and further suggest that teenagers may be more susceptible to the NIF impact of light. Future analyses on a larger sample are needed to confirm these results.

**Funding**: FNRS, ULiège, EU MSCA, FEDER, Fondation Léon Fredericq.

### 2.53. A Preliminary Investigation of Morning, Wearable, Short-Wavelength Light Therapy in Adolescents with Tourette’s Disorder: Subjective Outcomes

RickettsEmily^1^SwisherValerie^1^RissmanAriel^1^TookerMaya^1^ColesMeredith^2^McMakinDana^3^PiacentiniJohn^1^ColwellChristopher^1^BurgessHelen^4^1Department of Psychiatry and Biobehavioral Sciences, University of California, Los Angeles, CA, USA2Department of Psychology, State University of New York at Binghamton, Binghamton, NY, USA3Department of Psychology, Florida International University, Miami, FL, USA4Department of Psychiatry, University of Michigan, Ann Arbor, MI, USA

**Abstract: Background**: Circadian rhythm disruption is common in psychiatric and neurological disorders, with research also indicating delayed circadian timing in Tourette’s disorder (TD). Indeed, there is a later chronotype in TD relative to controls associated with higher tic severity. Morning light therapy (LT) is associated with circadian phase advances and improvements in core symptoms of psychiatric and neurological disorders, including TD. Case reports showed that morning LT was associated with modest to sizeable tic reduction. LT has historically involved emission of white light as high as 10,000 lux to the retina via a light box, but adherence to this modality may be reduced by adverse side effects from light brightness and burden due to limited mobility and time constraints. An alternative is wearable, short-wavelength LT, offering more flexibility and producing similar circadian phase shifts to white light but at lower light intensities. A pilot study in 14 adults with TD showed that two weeks of morning, wearable, short-wavelength LT was associated with a significant advances in circadian phase and reductions in tic severity, daytime sleepiness, and anxiety. However, research has yet to test morning LT in adolescents with TD. Therefore, this study investigated the degree to which this intervention was associated with improvements in chronotype, sleep-related outcomes, tic severity, and negative affect. **Methods**: Participants were 20 adolescents aged 13–17 years (M = 14.75, SD = 1.59) diagnosed with TD. An evaluator screened participants for eligibility using diagnostic and tic severity interviews. One week later, they completed a baseline assessment, immediately followed by two weeks of daily morning, short-wavelength wearable LT at their average baseline rise time (for 60 min per day). At baseline and postintervention, an evaluator administered a tic severity interview (Yale Global Tic Severity Scale) and children-rated chronotype (Children’s Morningness Eveningness Scale, insomnia and daytime sleepiness (Children’s Report of Sleep Patterns), anxiety and depression (Revised Children’s Anxiety and Depression Scale 25), and parents rated child sleep disturbance (Children’s Sleep Habits Questionnaire). **Results**: Paired sample t-tests were performed to evaluate change in clinical measures from baseline to posttreatment. There were significant reductions in child-reported insomnia (t = 2.37, *p* = 0.029) and daytime sleepiness (t = 2.36, *p* = 0.030), but no significant changes in parent-reported sleep disturbance (t = 0.63, *p* = 0.526) or child-reported chronotype (t = −0.54, *p* = 0.597). There were significant reductions in total tic severity (t = 2.78, *p* = 0.012) and motor tic severity (t = 3.10, *p* = 0.006), but not vocal tic severity (t = 1.76, *p* = 0.095), anxiety (t = 1.64, *p* = 0.102), or depression (t = 1.45, *p* = 0.146). **Conclusions**: Findings suggest benefit of morning LT for insomnia, sleepiness, and tic symptoms. Longer LT duration may be required to see an advance in chronotype. Addition of actigraphy and circadian phase assessment (i.e., dim light melatonin onset) measures are needed to determine objective sleep and circadian benefit. Future research should investigate the efficacy of this intervention in adolescents with TD in a randomized, sham-controlled trial.

### 2.54. Impact of Light on Health: Time Matters

ReidKathrynNorthwestern University, Evanston, IL, USA

**Abstract:** The light–dark cycle is a major synchronizer of circadian rhythms and has acute effects on neurobehavioral and metabolic function. The impact of light is dependent on when it occurs relative to the internal biological clock, and as such the timing of when we get light or dark across 24 h matters. Light exposure patterns are a modifiable factor that can have significant impact on health and well-being. Results from intervention and population-based studies that examine the impact of daytime light and of nighttime light exposure on health will be discussed. Light exposure both during the daytime and nighttime can influence glucose metabolism, for example, acute exposure to blue-enriched light exposure during the morning or evening while eating increased insulin secretion compared to a dim light control. A similar result was observed in a study in which exposure to 100 lux of room light on a single night during sleep was associated with an increase in the early response insulin secretion in response to an oral glucose tolerance test (OGTT) the next morning, compared to dim light. The changes in autonomic function during sleep in response to light were associated with the change in insulin in response to the OGTT the next morning. Interestingly while the light exposure during sleep altered autonomic function during sleep and next-day glucose metabolism, it did not suppress melatonin levels. These laboratory-based studies provide mechanistic support for findings from population-based studies that show a relationship between light exposure and cardiometabolic health. For example, we have shown that older adults who did not have at least 5 h of darkness at night (measured by wrist worn light sensor) had a greater risk of obesity, diabetes or hypertension. Using a similar approach in a large cohort of nulliparous pregnant women, we have shown that those who spent less time in dim light in the three hours before bedtime were more likely to develop gestational diabetes. Light can also impact mood, and in the same study of pregnant women we show that those who spent more time in dim light when awake were more likely to endorse depressive symptoms and have poor sleep quality. Taken together, these studies suggest that interventions that promote adequate daytime light and reduce nighttime light exposure should be developed to improve physical and mental health in vulnerable populations.

**Keywords:** metabolism; sleep; light; autonomic function; mood

**Funding**: Center for Circadian and Sleep Medicine Northwestern University, National Institute of Health: R01HL089695, R01HL090873, R01HL021010, UL1TR001422, P30AG059988, R01HL105549, U10HD063036, U10HD063072, U10HD063047, U10HD063037, U10HD063041, U10HD063020, U10HD063046, U10HD063048, U10HD073053.

### 2.55. Solar Exposure and Seasonality in Incident Depression, Bipolar Disorder and Purchase of Antidepressants

VolfCarlo^1^,^2^AagaardPeter Elm^3^EriksenEskild Soldal^1^,^2^DuboisJulie Margrethe^1^,^4^Wium-AndersenIda Kim^5^Wium-AndersenMarie Kim^5^OslerMerete^5^MartinyKlaus^1^,^4^1Mental Health Centre Copenhagen, University Hospitals Copenhagen, Copenhagen, Denmark2Department of Clinical Medicine, University of Copenhagen, Copenhagen, Denmark3Frederiksberg Hospital, Building 18, Frederiksberg, Denmark4Mental Health Center Copenhagen, New Interventions in Depression Group (NID), Department of Clinical Medicine, Copenhagen, Denmark5Center for Clinical Research and Prevention, Bispebjerg and Frederiksberg Hospitals, Frederiksberg, Denmark

**Abstract: Background**: Few Scandinavian studies have studied how seasonal variations affect incidents of depression, bipolar disorder and prescription of antidepressant medication in the general population. In Scandinavia, the daylight and daily hours of sunshine vary greatly from season to season: from 18 h of daylight during summer to 8 h of daylight during the winter. The prevalence of depression also varies, and for decades, we have known that a substantial part of the population experiences seasonal changes in their well-being. A subset even develops repeated major depressive episodes and seasonal affective disorders (SAD) in the winter season. The investigation of the impact of seasonality has previously mainly been by use of questionnaire surveys, whereas the use of general sociodemographic and health registers has not attracted much attention. The aim of this study is to use register-based information to examine seasonal variation in first-time prescription of antidepressants, as well as first-time hospital contacts of patients suffering from bipolar disorders or depression, to see whether any seasonal variation vary with solar exposure. **Methods**: We extracted monthly data from the Danish National Prescription Registry, the Danish National Patient Register and Danish Metrological Institute. Data from the Danish National Prescription Registry were based on information on all prescribed drugs dispensed at pharmacies, together with dates of prescription redemption from 2012–2021. Data from the Danish National Patient Register were based on access diagnoses from all psychiatric hospital contacts between 2012–2018 for patients aged 18–80 years. Data on sunlight incidence for all five Danish Regions, including data on temperature, covered the period 2012–2021. **Results**: During the study period, 477,925 individuals had purchased antidepressant medication and 65,815 and 11,760 had been diagnosed with depression and bipolar disorder, respectively. We found a seasonal effect on prescription rates of antidepressants being lower in summer and higher in winter. This coincided with sunlight incidence patterns at the weather stations. **Conclusions**: This is the first report on the seasonality in sunlight incidence and prescription rates of antidepressants. It raises several questions regarding how seasonal variations affect the well-being of the population in general. Variations in sunlight, which could underlie the observed increased prescription of antidepressants in the winter season, may be further affected by physical living conditions (floor level, geographical orientation, etc.) leading to less sunlight exposure, together with social behavior, such as daylight habits.

### 2.56. Effectiveness of Individualized Chronotherapy in Individuals with Subclinical Sleep Problems—Pilot Study

EvansováKatarína^1^,^2^ČervenáKateřina^1^SochůrkováAnna^1^,^3^NikoličMarek^4^GordijnMarijke^5^KopřivováJana^1^,^2^1Sleep and Chronobiology Research Centre, National Institute of Mental Health, Klecany, Czech Republic23rd Faculty of Medicine, Charles University, Prague, Czech Republic3Department of Psychology, Faculty of Arts, Charles University, Prague, Czech Republic4Psychedelic Research Centre, National Institute of Mental Health, Klecany, Czech Republic5Chrono@Work; University of Groningen, Groningen, The Netherlands

**Abstract: Background**: Disrupted and poor-quality sleep negatively affects overall health and well-being. However, a large population of individuals with subclinical sleep problems does not meet the diagnostic criteria for sleep disorders and remains therapeutically neglected. This study examines the impact of individualized chronotherapy on sleep quality in people with undiagnosed but restrictive sleep problems. It aims to validate the effectiveness of a specific combination of interventions for subclinical sleep difficulties and to test their medium-term effects duration. The intervention combines bright-light therapy (BLT), the use of blue-light-blocking glasses (BLB), sleep hygiene instruction (SHI), and individual adjustment of daily routine and sleep timing according to chronotype and preference. **Methods**: The research has three phases. In phase 1, screening questionnaires (PSQI, BDI, BAI, MEQ, MCTQ) are administered to identify exclusion criteria, such as major clinical disorders, determine the extent of sleep quality disruption and to identify circadian parameters to tailor individual chronotherapy. In phase 2, participants undergo baseline measurements (2 weeks) followed by a 3-week chronotherapeutic intervention. Both the experimental and control groups receive core interventions consisting of SHI and individual adjustment of daily routine and sleep timing according to chronotype and preference. The experimental group then receives morning BLT (timed according to MEQ score) along with evening BLB, while the control group receives only placebo glasses and no light therapy. Phase 3 includes a questionnaire at 4 weeks post-intervention. MotionWatch8 devices and sleep diaries are used to measure activity, a Lumie Vitamin L lamp is used for light therapy, and orange BLB glasses in the experimental group and clear glasses are used in the control group. **Results**: To date, a sample of 10 subjects has been obtained, 5 in each group (placebo/experiment). Due to small N and high inter-subject variability, t-statistics have not yet been calculated. The BDI, BAI and PSQI scales were collected at baseline, after the intervention and after 4 weeks. Baseline was removed from the scores and the results examined. Preliminary descriptive results show a stable improvement on all scales in the experimental group (an average of 4 points on the PSQI scale, 7 points on the BDI scale and 4 points on the BAI scale) compared to the control group. In terms of actigraphy data, we see an average 11% improvement in activity rhythm stability in the experimental group compared with a 3% improvement in the control group. **Conclusions**: Preliminary descriptive results show a steady improvement on all scales in the experimental condition compared to the control condition. Although these results should be interpreted with caution, we expect a moderate effect size for the intervention in the main study, with a target sample of 60 participants.

**Keywords:** chronotherapy; phototherapy; actigraphy; circadian rhythmicity; mood

**Funding**: Charles University Grant Agency grant 355322 (Effectiveness of individualized chronotherapy in individuals with subclinical sleep problems) and Technology Agency of the Czech Republic grant FW02020025 (Stable and mobile devices to support circadian synchronization, treatment and prevention of mental disorders through full-spectrum light phototherapy).

### 2.57. Variations in Fractal Patterns of Motor Activity across Sleep–Wake Cycles among Individuals with Varying Recency of Depression

MinaevaOlga^1^RieseHarriëtte^1^BooijSanne H.^1^,^2^LamersFemke^3^GiltayErik J.^4^ScheerFrank A. J. L.^5^,^6^HuKun^5^,^7^1University of Groningen, University Medical Center Groningen, Department of Psychiatry, Interdisciplinary Center Psychopathology and Emotion Regulation, Groningen, The Netherlands2Lentis, Center for Integrative Psychiatry, Groningen, The Netherlands3Amsterdam University Medical Centre, Department of Psychiatry, Amsterdam Public Health Research Institute, Vrije Universiteit, Amsterdam, The Netherlands4Leiden University Medical Center, Department of Psychiatry, Leiden University, Leiden, The Netherlands5Division of Sleep Medicine, Harvard Medical School, Boston, MA, USA6Medical Chronobiology Program, Division of Sleep and Circadian Disorders, Brigham and Women’s Hospital, Brigham, UK7Medical Biodynamics Program, Division of Sleep and Circadian Disorders, Brigham and Women’s Hospital, Brigham, UK

**Abstract: Background**: Outputs from healthy physiological systems, including motor activity, display fractal temporal fluctuations with a complex pattern balanced between randomness and excessive regularity, which reflects system integrity and adaptability. Alterations in fractal motor activity fluctuations are associated with various psychopathological disorders, such as (bipolar) depression and dementia. The current study examines whether and how fractal patterns vary across the sleep–wake cycle, differ in individuals with different recency of depression diagnosis (compared to non-depressed individuals), and change over time after a depressive episode. We expected individuals with more recent depression diagnoses to have the most different fractal patterns from the non-depressed individuals. **Methods**: Actigraphic recordings were collected from the participants in the Netherlands Study of Depression and Anxiety (NESDA), in which a 14-day actigraphy assessment was performed on 327 participants, including 43 with a current depression diagnosis (within 1 month), 47 with recent depression (within 6 but not within 1 month), 151 with remitted depression (a lifetime diagnosis but not within 6 months), and 86 controls (without a history of depression). To evaluate fractal patterns across the sleep–wake cycle, detrended fluctuation analysis (DFA) was performed in each non-overlapping 4 h window to derive a scaling exponent, α, that quantifies temporal correlations in motor activity fluctuations across different time scales. Linear mixed models were used to determine the effects of the sleep–wake cycle, group, and their interaction on the scaling exponent. Sleep–wake schedules were determined from the actigraphy data. **Results**: In all individuals, α showed a significant variation across the sleep–wake cycle with larger values during wakeful periods (α = 1.035 ± 0.003, indicating stronger temporal correlations) and smaller values during sleep periods (α = 0.784 ± 0.004, *p* < 0.001, indicating more random activity fluctuations). The variation in α was significantly different between groups (*p* = 0.02). Specifically, during sleep, the recent depression group had significantly larger α (0.83 + −0.009) than the control group (0.78 + −0.013, *p* = 0.010), the current depression group (α = 0.77 + −0.018, *p* = 0.013), and the remitted depression group (α = 0.77 + −0.012, *p* = 0.002). During wakefulness, there were no significant group differences in α (all *p* values > 0.05). **Conclusions**: Fractal activity patterns varied across the sleep–wake cycle, and the variation was reduced (i.e., fractal activity patterns during sleep resembled more the patterns during wakefulness) in individuals with recent depression, but not in those with current depression or in remission. These findings suggest a delayed effect of a depressive episode on activity patterns during sleep.

**Keywords:** sleep–wake rhythm; sleep; motor activity; fractal regulation; depression

**Funding**: This study was supported by the European Research Council (ERC) under the European Union’s Horizon 2020 research and innovative programme (ERC-CoG- 2015; 681466 to M. Wichers). O.M. has been supported by the Foundation “De Drie Lichten” in the Netherlands and by the Van der Gaag Fund, Royal Netherlands Academy of Arts and Sciences. F.A.J.L.S. has been supported in part by RF1AG059867. K.H. has been partially supported by NIH grants (RF1AG059867, RF1AG064312) and the BrightFocus Foundation Award (A2020886S).

### 2.58. Children’s Daily Light Exposure and Sleep across Seasons in Northern Sweden

HeydenMaja^1^KönbergKristin^2^Ala-PrinkkiläAleksi^2^WulffKatharina^2^1Julius-Maximilians University of Würzburg, Würzburg, Germany2Umeå University, Umeå, Sweden

**Abstract: Background**: Daylight length varies more over the annual cycle the further away people live from the Equator. Every point on Earth experiences on average 12 h of light per day, but the actual number of hours of daylight on any given day of the year varies from place to place. Our daily sleep–wake rhythm closely follows the 24 h light–dark cycle, but how daily rest–activity patterns vary in relation to the abundance of daylight in people living at higher latitudes is not well documented. This ongoing study is aimed at developing tools to quantify daylight and rest–activity changes for use in a large cohort. **Methods**: Three-year-old children, enrolled in the Northpop cohort of Region Västerbotten in northern Sweden on the Gulf of Bothnia, have been taking part in longitudinal, actigraphic data collection since 2020. Time spent outdoors from diary entries as well as rest–activity and light exposure data are being collected in 30-second epochs over eight days using wrist-worn MotionWatch 8 devices. Actigraphically derived sleep parameters are being analysed using MotionWare and Excel-based linked macros have been developed to analyse light and activity data. **Results**: Preliminary data from a subset of 169 children are evenly distributed across seasons and reveal greater inter- than intra-individual differences in sleep parameters. Overall, time-based sleep parameters show less variability than movement-based parameters. Average patterns of light levels over the 24 h day, indicating the proportion of time spent in different light levels, demonstrate the impact of season on daytime versus evening light. The proportion of evening light was found to vary more in summer than in any other season. **Conclusions**: The organisation of daily activity patterns and sleep parameters appears to follow a seasonal synchronisation.

**Keywords**: photoperiod; seasonality; circadian rhythm; paediatrics; behaviour; sunlight

**Funding**: This study is partly funded by the Wallenberg Foundation and Insamlingsstiftelsen.

### 2.59. Phase Shifting of Circadian Glucose Rhythms in Response to Large Meals

IsherwoodCheryl^1^JohnstonJonathan^2^SkeneDebra^1^VeenDaan Van der^1^JacksonNicola^1^BurdenAbbie^1^HassaninHana^1^1Chronobiology, University of Surrey, Surrey, UK2Univertsity of Surrey, Surrey, UK

**Abstract: Background**: There is substantial evidence for food anticipation in rodents that is intrinsically driven by a food-entrainable oscillator. To date, however, evidence of human food anticipatory physiology is lacking. In a controlled laboratory protocol, we tested the hypothesis that human circadian metabolic physiology anticipates two large meals, but not small hourly meals. **Methods**: Following a controlled ten-day at-home entrainment protocol, 24 healthy young men underwent an 8-day laboratory study. Days 1–6 of the laboratory protocol (entrained conditions) comprised a strict sleep–wake and light–dark schedule (lights out 23:00 h, lights on 7:00 h ± 10 min) with controlled meal content and timing. One group of participants was given two large meals (LMs) at 7.5 and 14.5 h after lights on, and the other group received 14 small meals (SMs) hourly, commencing 1.5 h after lights on. Starting on day 7, both groups entered a 37 h constant routine (CR). Throughout the entire laboratory protocol, interstitial glucose monitors measured glucose every 15 min. In the CR, commencing 3.5 h after waking, plasma samples were collected every 30 min for 30 h (*n* = 60 samples per participant). Saliva was collected every hour for 30 h, starting 4 h from waking. In these samples, plasma glucose, triglycerides, total cholesterol and HDL cholesterol, and saliva melatonin were determined. Non-numerical, visual analogue scales assessed appetite hourly on days 2, 4 and 6 and throughout the CR. Interstitial glucose and plasma data were corrected to the time of melatonin onset (DLMO). **Results**: Between groups, no significant difference in age, BMI or DLMO was observed. Interstitial glucose concentrations increased in the early morning in both groups on days 1–6. During these entrained conditions, glucose concentrations decreased from 2 h after waking until the first meal in the LM group, but in the SM group, they continued to increase across the day. Average 24 h interstitial glucose concentrations did not differ between groups. In the CR, antiphasic interstitial glucose rhythms were observed (acrophase −6.13 ± 0.64 vs. 6.28 ± 1.01 h, SM vs. LM groups respectively; *p* < 0.001), with low glucose at the time of previous meals in the LM group. Plasma glucose rhythms were delayed 7 h in the LM group compared to the SM group (SM −1.12 h, LM 6.26 h according to DLMO). By contrast, no phase delay was observed for triglycerides, total cholesterol or HDL cholesterol. Triglycerides peaked at approximately 6 h according to DLMO, whereas total cholesterol and HDL cholesterol peaked at approximately 4-h according to DLMO in each group. **Conclusions**: Administration of two large meals significantly phase-shifted interstitial and plasma glucose rhythms in constant routine conditions. By contrast, there was no effect of the large meals on plasma triglycerides or HDL cholesterol.

**Keywords:** chrononutrition; food anticipation; circadian clock; continuous glucose monitoring; peripheral clocks; circadian rhythms; meal timing; melatonin

**Funding**: UK BBSRC (BB/S01814X/1).

### 2.60. Modern Lifestyle and Desynchronization—What Factors Do Really Matter?

MykytyukOksanaBukovinian State Medical University, Chernivtsi, Ukraine

**Abstract: Introduction**: Modern achievements of science and technologies within the last 150 years have changed our lives dramatically: artificial lights overpower the darkness and our cities glow at night, disrupting the natural day–night pattern and shifting the delicate balance of our environment. Increased demand for 24/7 activities has contributed to modification of work schedule and leisure events of the population dramatically in almost all continents. The negative effects of the loss of natural resources and disrupted rhythmicity might seem invisible, but later multiple studies have documented a correlation between night-shift work and the increased incidence of breast and prostate cancer, arterial hypertension, obesity, type 2 diabetes mellitus, cardiometabolic illnesses, depression and anxiety, all of which impose major public health and economic burden on societies. A lot of studies are dedicated to occupational risks, but few pay attention to habits and everyday life routine as potential factors of lifestyle determination. The goal of the present investigation was to establish: (1) age at which the induction of lifestyle modification starts and (2) what factors are playing a key role in day–night reversal in different lifespan periods. **Methods**: Questioning was undertaken among representatives of different age groups: from 14 to elderly people. Target groups included teenagers 14–16 (68 people), 16–18 (46 graduates of school), and students 18–25 (90 people); healthy 25- to 40-year-olds (55); 41- to 60-year-olds (58) and elder ones (47). We used a lifestyle and health questionnaire, a modified wellness and lifestyle questionnaire with a focus on daily activities, and a chronotype determination questionnaire. **Results**: Before 24 February, 2022, teens reported significance of studies till late night hours while studying at school and preparing for entering higher-education establishments. At around 16–17, younger ones had to follow family rules and cases of late activity were occasional, related mostly to gaming and leisure activities. Students mentioned significance of studying, social activities, night life (especially at weekend), and around 20% were shift workers. Subjects aged 25–40 and 41–60 marked the importance of occupational activities and care of infants, but a majority mentioned that these factors were transient and sufficient sleep was a useful stable habit. Elder people reported significance of sleep disturbances, frequent joint pain and care of family. Since 24 February 2022, the Ukrainian population has been facing new challenges: air raids, increased anxiety, chronic stress, and loss of occupational + volunteering activities. Three months of regular blackouts made people adjust to everyday routine activity to certain hours of access to electricity. **Conclusions**: Frequency of insomnia and depression increased dramatically, and it seems that health consequences as result of chronic desynchronization and desadaptation will require correction for decades.

### 2.61. Advancing Sleep Timing to Improve Depression

HoubyAnne Sofie Kvist^1^MadsenHelle Østergaard^1^KraghMette^2^BaandrupLone^1^WulffKatharina^3^MartinyKlaus^1^,^4^1New Interventions in Depression (NID-Group), Mental Health Centre Copenhagen, University of Copenhagen, Copenhagen, Denmark2Department of Affective Disorders, Aarhus University Hospital Psychiatry, Aarhus, Denmark3Departments of Molecular Biology and Radiation Sciences, Faculties of Medicine, Science and Technology, Umeå University, Umeå, Sweden4Mental Health Center Copenhagen, New Interventions in Depression Group (NID), Department of Clinical Medicine, Copenhagen, Denmark

**Abstract: Background**: Patients developing a depressive episode often also develop a delayed sleep timing. This is partly thought to be due to diminished zeitgeber inputs to the circadian system from light and other cues such as dieting, social contact, and exercise. This diminished input is partly driven by behavioral changes caused by the depressive symptoms. Another reason for the delayed sleep timing is diurnal variation in depression symptoms, with morning low and evening improvement, luring patients to postpone their sleep timing. There is a powerful relationship between sleep timing and depression, most evident in the acute effect of wake therapy, but also in regimes using sleep phase advance. Wake therapy and sleep phase advance are both effective antidepressant methods, but time-consuming, and require a substantial effort on the part of the patients and the staff in psychiatric services. There is a need for easy-to-implement chronobiological treatments to help patents with depression recover faster and more completely form their depressive episodes. **Methods**: In all, 100 patients with major depression treated in a psychiatric outpatient unit will be randomly allocated to either standard care (TAU group) or standard care plus the newly developed treatment method circadian reinforcement therapy (CRT group). Only patients that have drifted more than 1 h from their habitual sleep schedule will be included (enriched sample). Patients in the CRT group will be psychoeducated on chronobiological principles to help them increase their zeitgeber inputs from daylight, exercise, social contact, and dieting. In addition, they will receive advice on sleep timing/sleep hygiene and CBT-I principles to reduce anxiety-related sleep-onset insomnia. Patients will be followed for 8 weeks and will be wearing a Camntech MotionWatch 8 to assess sleep timing, 24 h activity, night-sleep duration, night awakenings, sleep efficiency, daytime napping, and light exposure. The primary outcome is change in Hamilton Depression Rating scale (HAM-D17) scores from baseline to endpoint. **Results**: The study will begin recruiting in September 2023. **Perspectives**: If the CRT method can induce a correction of delayed sleep timing and an associated improvement in depression, this method can be implemented as part of standard treatment at in- and outpatient psychiatric services. The method only requires basic knowledge of the circadian system and psychoeducation tools. The method is easy to use for patients and staff.

**Keywords:** depression; sleep phase advance; chronotherapy; actigraphy; circadian reinforcement therapy

**Funding**: Independent Research Fund Denmark.

### 2.62. Melanopic Irradiance of Metameric Display Light in the Evening Affects Objective Sleepiness as Indexed by a Change in the Alpha Attenuation Test

StefaniOliver^1^,^2^SchöllhornIsabel^1^,^2^CajochenChristian^1^,^2^1Centre for Chronobiology, Psychiatric Hospital of the University of Basel, Basel, Switzerland2Research Platform Molecular and Cognitive Neurosciences, University of Basel, Basel, Switzerland

**Abstract: Introduction**: Visual displays emit optical radiation at short wavelengths, close to the peak sensitivity of melanopsin. A measure to quantify the strength of non-image-forming (NIF) responses to light is the “melanopic equivalent daylight illuminance” (mEDI). We therefore investigated the effects of changes in display illumination on the alpha attenuation test (AAT), a validated objective marker of human sleepiness levels. The AAT index is expressed as the ratio of the power density in the EEG alpha range (8–13 Hz) between eyes closed and open. A higher AAT index indicates less objective sleepiness or more alertness. **Methods**: Spectrally different white lights that are perceived as the same white tone are called metamers. We developed a metameric display backlight using five LED types (440, 480, 500, 550 and 620 nm). By shifting the peak wavelengths of the primary colors, equal stimulation of the three cone types but different stimulation of melanopsin (high (HM) and low (LM) (factor “condition”)) was achieved. The study design was a pseudo-randomized mix of a within- and between-subject design. Seventy-two healthy male participants were examined twice (within factor: HM and LM) under controlled laboratory conditions and divided into four luminance groups (between-factor).

- Group 1: Luminance 27 cd/m^2^, (illuminance ≈ 8 lx), HM mEDI 15 lx, LM mEDI 4 lx
- Group 2: Luminance 62 cd/m^2^, (illuminance ≈ 20 lx), HM mEDI 33 lx, LM mEDI 9 lx
- Group 3: Luminance 135 cd/m^2^, (illuminance ≈ 40 lx), HM mEDI 70 lx, LM mEDI 21 lx
- Group 4: Luminance 284 cd/m^2^, (illuminance ≈ 80 lx), HM mEDI 146 lx, LM mEDI 48 lx

The AAT indexed was assessed via EEG recordings during the Karolinska Drowsiness Test (KDT). Each KDT consisted of a 5 min open-eye period followed by a 3 min closed-eye period during which participants were asked not to blink. The AAT index was calculated for each KDT. While twelve EEG leads were recorded, here, we report results from one frontal lead (Fz). Depending on the signal quality, Fz was replaced by F4 or F3. **Results**: Data from 72 volunteers entered statistical analysis. Only during the highest luminance (284 cd/m^2^) was the factor condition (HM vs. LM) significant (F_(1,83)_ = 6.48, *p* = 0.01), with a higher AAT (i.e., more alertness) during HM. The factor “time” was not significant for any group. There was no interaction for the factor “time*condition.” **Conclusions**: Depending on the mEDI, a commonly experienced display luminance of 284 cd/m^2^ affects objective sleepiness as indexed by a higher AAT index during HM. Thus, reducing the mEDI of electronic displays at a typical illuminance of ≈80 lx is a successful approach to limiting the alerting effects of light before bedtime without altering the perceived white tone.

**Acknowledgments**: This project was funded by the SNSF Project 320030_182266.

**Disclosures:** Nothing to disclose.

### 2.63. Circadian Intervention Study in Elderly with Low Sleep Quality: A Randomized Controlled Trial

OtteJohanna^1^,^2^,^3^†ReckelsSophie Charlotte^1^,^2^,^3^†CajochenChristian^1^,^2^,^3^GarbazzaCorrado^1^,^2^,^3^SpitschanManuel^4^EppleChristian^1^MeyerMartin^5^SlawikHelen^5^DeuringGunnar^1^,^2^,^3^EckertAnne^5^KawasakiAki^6^JaeggiSusanne^7^MünchMirjam^1^,^2^,^3^1Centre for Chronobiology, Psychiatric Hospital of the University of Basel, Basel, Switzerland2Research Platform Molecular and Cognitive Neurosciences, University of Basel, Basel, Switzerland3Integrative Human Circadian Daylight Platform (iHCDP), Basel, Switzerland4Max Planck Institute for Biological Cybernetics & Technical University of Munich, Technical University of Munich, Munich, Germany5Departement Klinische Forschung, Psychiatric Hospital of the University of Basel, Basel, Switzerland6Hôpital Ophtalmique Jules Gonin, Fondation Asile des Aveugles, University of Lausanne, Lausanne, Switzerland7Center for the Neurobiology of Learning and Memory NOT SURE, University of California, Irvine, CA, USA†These authors contributed equally to this work.

**Abstract: Background**: High-quality sleep is an essential pillar for general health and well-being. Many older adults report low sleep quality, often without known physical or psychological causes and co-morbidities. To date, the best-known non-pharmacological treatment strategy is cognitive behavioural therapy. However, there is evidence suggesting that different chronotherapeutic interventions can effectively improve sleep quality. Such interventions intend to increase external zeitgeber strength, for example, implementation of sleep hygiene rules, scheduled physical activity, light exposure, or time-restricted eating. The aim of this study is to investigate the long-term effectiveness of a multidomain and personalised circadian intervention combining sleep hygiene, light exposure, physical activity, and time-restricted eating and assessing potential synergistic effects. The primary outcome is subjective sleep quality assessed by the Pittsburgh Sleep Quality Index (PSQI). **Methods**: The study is designed as a randomised controlled trial (registered at: https://clinicaltrials.gov) with a target sample size of 86 older women and men (age ≥ 65 years) reporting low subjective sleep quality (PSQI > 5). Exclusion criteria are: total blindness or visual acuity <0.5, progressive noncommunicable diseases, acute infections (e.g., COVID-19), neurodegenerative diseases, psychiatric diseases, diagnosed sleep disorders (e.g., sleep apnea or narcolepsy), or not German-speaking. After a screening visit at the Centre for Chronobiology (including a medical examination and a blood sample), participants perform baseline measurements at home for seven days. The next step is an instruction visit at the participants’ home, during which scheduled interventions (based on baseline assessments) are discussed with participants of the intervention group and are adjusted to their needs in a co-design approach. Participants in the control group receive general sleep hygiene recommendations instead. Following the instruction visit, all participants are asked to implement the study interventions independently at home for 12 months. Every three months, baseline assessments are repeated (for 14 days) and participants are visited at their homes during the day. Among other measures, subjective sleep quality (PSQI) and objective sleep (derived from actigraphy) are assessed. In addition, physical activity, sleep–wake patterns, light exposure, pupillometry, and meal timing are monitored. During repeated visits at participants’ homes, adherence to the protocol is verified and cognitive functions are assessed by testing executive functions, working memory and reaction time. For participants in the intervention group, interventions are readjusted if necessary. After 12 months, data collection ends and participants have a final interview at the Centre for Chronobiology. Statistical analysis with a mixed within–between subject approach with generalized linear regression models will be used to determine hypothesised differences in the time course within each group and differences between the intervention and the control group. **Expected Results**: The study started in August 2022, and so far, 35 participants have been enrolled. We expect to detect significantly greater subjective sleep-quality improvements in the intervention group compared to the control group.

**Keywords:** multi-domain; personalised; circadian; sleep; light exposure; physical activity; time-restricted eating; daylight

**Funding**: This project is part of the international integrative Circadian Human Daylight Platform (iHCDP; www.ihcdp.org) and is financially supported by the VELUX Stiftung (Switzerland).

### 2.64. Non-Linear Relationship for Reaction Time and Melanopic EDI—Is There an Optimum for Office Lighting?

MünchMirjam^1^,^2^BenedettiMarta^3^MaierovaLenka^4^CajochenChristian^1^,^2^SchmidtChristina^5^ScartezziniJean-Louis^3^1Centre for Chronobiology, Psychiatric Hospital of the University of Basel, Basel, Switzerland2Research Platform Molecular and Cognitive Neurosciences, University of Basel, Basel, Switzerland3Solar Energy and Building Physics Laboratory (LESO-PB), Ecole Polytechnique Fédérale de Lausanne (EPFL), 1015 Lausanne, Switzerland4University Centre for Energy Efficient Buildings, Czech Technical University in Prague, Trinecka 1024, Bustehrad, Czech Republic5Sleep and Chronobiology Laboratory, GIGA-CRC-IVI, University of Liège, Liège, Belgium

**Abstract: Background**: Lighting during the day affects task performance, alertness, mood, and well-being. Most of the evidence for good lighting comes from laboratory studies with comparisons to rather dim light conditions. We recently conducted a study using a mixed daylight/electric lighting paradigm, including a daylight control condition [10]. The aim was to determine whether there is a dose-dependent relationship between cognitive performance and daytime light exposure. **Methods**: For this exploratory analysis, we used the data from our previous study [1]. In the original study, a total of 34 young healthy participants spent 5 consecutive days (for 8 h) in an office room with automated blinds and electric lighting controls and larger windows. In a balanced cross-over design, they also spent 5 consecutive days in a control office room without a controller. Measurements in the experimental rooms started 1 h after habitual wake-up time. Every 2.5 h, participants completed a battery of auditory cognitive tests including the Psychomotor Vigilance Test (PVT). A total of 40 PVT sessions were completed. The 10% fastest reaction times (RTs) per session were determined and averaged per participant per day. For half of the participants (*n* = 17), light exposure was continuously monitored with a stationary spectroradiometer in the vertical direction at the participants’ eye level. Melanopic equivalent daylight illuminance (melanopic EDI) was calculated according to international standards [11] and was averaged per participant per day. A non-linear curve fit was applied to the transformed RTs (i.e., 1/RTs) and melanopic EDI (lux). **Results**: The shape of the raw scatterplot suggested an inverted U-shaped relationship between RTs and melanopic EDI. The non-linear curve fit revealed a weak, but statistically significant regression (R^2^ = 0.13; *p* < 0.001). From the fitted curve, the fastest RTs lasted 196 ms (peak), corresponding to 992 melanopic lux. The 5% ranges around this maximum showed 10 ms slower RTs in both directions, corresponding to a range between 321 and 1652 melanopic EDI. **Conclusions**: We found evidence for a significant non-linear relationship between the fastest 10% RTs of the PVT and melanopic EDI. This finding suggests that there may be a lower and an upper threshold for optimal illumination and RT. This certainly needs to be further explored with larger samples and different cognitive tasks. Interestingly, the “optimal” range of melanopic EDI for RTs in this sample was above the recently recommended minimum of 250 melanopic EDI during daytime [12]. It should also be noted that the subjective glare was not perceived as uncomfortable (as assessed by the daylight glare probability index).

**Keywords:** daylight; circadian; melanopic; cognition; office lighting; DGP; PVT; non-visual; dose–response

**Funding**: Swiss Innovation Agency Innosuisse and NEST SolAce at EMPA (Swiss Federal Laboratories for Materials Science and Technology) and the Velux Foundation, Switzerland.

No conflicts of interest.

**References**

10. Benedetti, M.; Maierová, L.; Cajochen, C.; Scartezzini, J.L.; Münch, M. Optimized office lighting advances melatonin phase and peripheral heat loss prior bedtime. **2022**, *12*, 4267. https://doi.org/10.1038/s41598-022-07522-8.
11. *CIE S026:2018*; System for Metrology of Optical Radiation for ipRGC-Influenced Responses to Light. CIE: Vienna, Austria, 2018. https://doi.org/10.25039/S026.2018.
12. Brown, T.M.; Brainard, G.C.; Cajochen, C.; Czeisler, C.A.; Hanifin, J.P.; Lockley, S.W.; Lucas, R.J.; Münch, M.; O’Hagan, J.B.; Peirson, S.N.; et al. Recommendations for daytime, evening, and nighttime indoor light exposure to best support physiology, sleep, and wakefulness in healthy adults. **2022**, *20*, e3001571. https://doi.org/10.1371/journal.pbio.3001571.

### 2.65. Assessing Genetic Variation for Effects of Lithium on Circadian Clock Period, Sleep Behavior, and Mortality in Fruit Flies

FryouNoahLiMingjiaPossidenteBernardSkidmore College, Saratoga Springs, NY, USA

**Abstract:** Lithium is known for its mood-stabilizing effects and is a treatment of choice for bipolar disorder, a cyclic disorder with alternating bouts of mania and depression. Although the mechanism of lithium’s therapeutic effect is unknown, it typically slows down circadian clocks, decreases REM sleep, and increases REM sleep latency, suggesting that circadian and/or sleep mechanisms may be involved ^3^. Patients vary in their response to lithium as it can be toxic in high doses. We used a random sample of 13 inbred, 3 wildtype, and 2 hybrid strains from the *Drosophila* Genetic Resource Panel to examine genetic variation for the response of circadian clock period and sleep behavior to 20 mM LiCl, and its toxicity. Adults were assayed for circadian locomotor activity and sleep phase in constant dark using *Drosophila* activity monitors. Circadian clock period and sleep were estimated using a chi-squared periodogram. Among 13 inbred strains examined, there was no significant effect in lithium or genetic variation for effects of lithium on circadian period, and only one inbred strain significantly had lengthened circadian clock period in response to lithium. There was a significant sex difference in the response to lithium for circadian period, with an increase in females and a slight decrease in males, and significant strain variation for sex differences in circadian response to lithium. There were significant strain differences in mortality in response to lithium. All three wild-type strains showed significantly increased circadian period in response to lithium and varied in sensitivity to its toxicity. Significant treatment, sex, and gene difference for the effect of lithium on sleep phase was observed, additional, and significant treatment, gene, and sex differences for the effect of lithium on mean sleep were observed. A significant treatment, sex, and gene difference for the effect of lithium on the average length of sleep bouts was also observed. Results to date suggest that the set of approximately 200 DGRP strains will be useful for investigating genetic variation for lithium toxicity and sex differences for effects of lithium on circadian clock period. The circadian period in wild-type strains is more responsive to the period-lengthening effects of lithium, suggesting that genetic heterozygosity may play a role in the effects of lithium on the circadian clock period.

### 2.66. Daylight Variability and Its Role in Shaping Visual and Non-Visual Responses

YuCehao^1^PastilhaRuben^2^PontSylvia^1^HurlbertAnya^2^1Faculty of Industrial Design Engineering, Delft University of Technology, Delft, The Netherlands2Institute of Neuroscience, Newcastle University, The Netherlands

**Abstract: Background**: Natural daylight arises from a complex interplay of sunlight and skylight, resulting in spectral irradiance fluctuations across various time scales, spatial locations, and directions. Although the spectral irradiance of sunlight itself remains fairly constant, and the sun’s systematically changing elevation causes systematic changes in natural illumination, the interactions of sunlight with the atmosphere and the often large variations in atmospheric conditions may make daylight variations unpredictable. Light fields, which describe the spectral irradiance of light arriving from every direction at every point in the scene, allow for capturing multi-dimensional illumination variations. Understanding these variations and their impact on visual perception and non-visual responses can contribute to optimising lighting design and enhancing human well-being. **Methods**: Light-field data from two sources (the Delft spectral light-field database and new measurements in Newcastle) encompassing spherical light-field measurements with high temporal resolution over full days were collected and analysed. Unlike traditional downwelling irradiance measurements and 2D hyperspectral imaging, these data enabled decomposition of the global illumination into time-varying directional and diffuse components. **Results**: The analysis revealed a distinct three-part pattern during sunny days. Before sunrise and after sunset, both diffuse and directional illumination components were “blueish.” Diffuse illumination then fluctuated within a higher CCT range (5000–7500 K), while directional components showed gradual shifts from lower (around 4000 K) to higher CCTs (approximately 6000 K) and then back again. The most rapid chromaticity changes occurred in the early morning and late afternoon, with a comparatively stable interim period. Illuminance changes were governed by solar elevation and typically most rapid at the beginning and end of the day, yet in cloudy conditions, chromaticity and illuminance changes became irregular and were heavily influenced by weather-related factors. On sunny days, illumination directions followed the sun’s positions when diffuseness was low. On cloudy days, illumination directions varied due to scattering by clouds. Altitude of the light direction and diffuseness were correlated in both weather conditions. In general, change speeds were faster for directional vs. diffuse components, particularly for illuminance. **Conclusions**: Comparing these results with data from psychophysical studies of illumination change discrimination (e.g., Pastilha et al. 2020), we conclude that the systematic changes in natural daylight generally transpire too gradually for direct visual detection, apart from rapid changes prompted by swift cloud movements that unveil or obscure the sun. We hypothesise that visual mechanisms are optimised to reduce sensitivity to natural illumination chromaticity changes, thereby aiding stability of object colour perception. Non-visual mechanisms appear attuned to the natural chromaticity changes at dawn and dusk, enabling synchronisation of the circadian clock with environmental conditions. Non-visual mechanisms are also suited, via their retinal distribution and temporal response characteristics, to register only the slower changes in the diffuse component of global illumination, thereby aiding stability of non-visual behaviour and physiology. We suggest that it is important to consider the differential effects of directional vs. diffuse components when incorporating temporal dynamics into light design.

**Keywords:** daylight; temporal variations; light field; visual perception; non-visual response

**Funding**: DyViTo (EU Grant number 765121); Newcastle University.

### 2.67. Melanopic Irradiance-Dependent Effects on Pupil Size and Cognitive Performance Using Metameric Display Light

SchöllhornIsabel^1^,^2^StefaniOliver^1^,^2^EppleChristian^1^CajochenChristian^1^,^2^1Centre for Chronobiology, Psychiatric Hospital of the University of Basel, Basel, Switzerland2Research Platform Molecular and Cognitive Neurosciences, University of Basel, Basel, Switzerland

**Abstract: Introduction**: Evening exposure to display light has been shown to increase alertness and cognitive performance. There is also evidence that selective modulation of melanopic irradiance affects alertness levels and that melanopsin-stimulating signals contribute more to the pupillary pathway than the L- and M-cone signals. Here, we aimed to observe melanopsin-dependent effects on psychomotor vigilance (PVT) and response inhibition (Go/NoGo) while measuring pupil size. **Methods**: Seventy-two healthy, male participants completed a 2-week study protocol. The volunteers were assigned to one of four groups that differed in luminance levels (27–285 cd/m^2^). Within the four groups, each volunteer was exposed to a low melanopic (LM) and a high melanopic (HM) condition. The metameric LM and HM differed in melanopic equivalent daylight illuminance (mEDI): Group 1: mEDI 4 lx vs. mEDI 15 lx, Group 2: mEDI 9 lx vs. mEDI 33 lx, Group 3: mEDI 21 lx vs. mEDI 70 lx, Group 4: mEDI 48 lx vs. mEDI 146 lx. The two 17 h study protocols comprised 3.5 h of light exposure starting 4 h before habitual bedtime. Test sessions were conducted before, during and in the morning after light exposure and included auditory tasks to test sustained attention and reaction time (PVT) and response inhibition (Go/NoGo task). During the test sessions, pupil size was continuously recorded using an eye-tracking device. **Results**: Median pupil size was significantly smaller during HM than LM in all four light-intensity groups. We also found a significant correlation between log10 mEDI and pupil size, such that higher mEDI values were associated with smaller pupil sizes. Only in one light-intensity group per task (PVT: Intensity 3, Go/NoGo: Intensity 1) were response times during HM significantly faster than LM (*p* < 0.05). There were no significant effects of light condition on the fastest or the slowest responses and the rate of omission errors within any of the four intensity groups. In an exploratory analysis, we combined the data from all four light-intensity groups. Median correct response time (Go/NoGo) showed a significant interaction between light condition and time of day, such that it was significantly faster during HM than during LM in the last test session, performed 1 h before bedtime. **Conclusions**: Our data identify melanopic irradiance as a valid parameter for the prediction of pupil size, even at rather low light levels (<90 lx). In the present study, the effects of melanopic irradiance on cognitive performance were small and not consistent.

**Funding**: This project was funded by the SNSF Project 320030_182266.

### 2.68. Non-24 h-Sleep–Wake Disorder in Sighted Individuals

RijnKarin van^1^EvansováKatarína^2^,^3^GordijnMarijke^2^1Sleep Wake Center SEIN Zwolle, Zwolle, The Netherlands2Chrono@Work, Groningen, The Netherlands33rd Faculty of Medicine, Charles University, Prague, Czech Republic

**Abstract:** A non-24 h sleep–wake disorder (N24SWD) occurs in ±67% of blind patients without light perception (Flynn-Evans et al., 2014). This sleep disorder can be recognized by a typical non-entrained pattern in sleep–wake rhythms. A N24SWD in sighted people is assumed to be rare (Garbazza, 2018) and to be preceded by a delayed sleep–wake phase disorder (DSWPD) (Hayakawa et al., 2005; Malkani et al., 2018). The aim of our study is to estimate and compare the variance in the free-running periods of the activity–rest cycle based on actigraphy as well two 24 h melatonin curves collected under ambulatory conditions 2–3 weeks apart in sighted N24SWD patients in our tertiary sleep clinic. Co-morbidity of the group of patients suspected to have a diagnosis of N24SWD will be described. **Methods**: From 2018–2022, 74 adults (≥18 years) sighted patients (15 female and 59 male) visited our sleep clinic and a N24SWD was suspected. Data of 1–3 weeks of actigraphy and repeated assessment of the 24 h rhythm of melatonin, with an interval of 2–3 weeks as well as additional medical and psychiatric history was obtained. Dim-light melatonin onset (DLMO based on 3 pg/mL) of the two consecutive assessments were compared. The endogenous circadian period (tau) under “entrained conditions” was calculated based on the drift in DLMO over the interval of 2–3 weeks as well as on midsleep in the actigraphy data. **Results**: Actigraphy and two melatonin curves were available in 39 subjects (10 f/29 m), average age 28 years (18–57). Preliminary analysis shows that the endogenous period of the rest–activity rhythm under entrained conditions ranges from 21.5 till 26.4 h with an average of 24.2 ± 0.4 h. The melatonin period ranges from 24.0 to 25.3 h with an average of 24.4 ± 0.7 h. A positive correlation was found between tau estimated based on melatonin and rest–activity rhythm (Rs = 0.4, *p* < 0.05). All patients (100%) with confirmed N24SWD (24 patients, 6 f/18 m) had a history of DSWPD. The vast majority of patients (92%) were diagnosed with a neurodevelopmental disorder (autism spectrum disorder (ASD) and/or attention-deficit/hyperactivity disorder (ADHD). Prior substance abuse (cannabis and/or hard drugs) was reported by six patients (27%). Half of the patients (*n* = 12) reported a vitamin D deficiency. All patients fulfilled the criteria for another psychiatric diagnosis varying from prior drug/game addiction to depression, anxiety, bipolar and obsessive compulsive disorder. **Conclusions**: There is a large variation in tau based on actigraphy and tau based on DLMO under entrained conditions in this specific group of sighted sleep disorder patients suspected of N24SWD. The psychiatric phenotype consists mainly of young males with a comorbid neurodevelopmental disorder and a vitamin D deficit. Other risk factors might be a prior diagnosis of DSWPD and/or other psychiatric co-morbidity and the use of (soft) drugs. Our hypothesis is that endogenous characteristics of the biological clock in combination with behaviour underlie the free-running pattern in both the sleep–wake cycle and the rhythm of melatonin. We advise to be alert to N24SWD, especially in young males with a neurodevelopmental disorder.

### 2.69. Prefrontal Cortex Neurons Encode Ambient Light Intensity Differentially across Regions and Layers

SabbahShaiZangenElyashivDepartment of Medical Neurobiology, Faculty of Medicine, The Hebrew University of Jerusalem, Jerusalem, Israel

**Abstract: Background**: The medial prefrontal cortex (mPFC) regulates emotion and cognition, while mPFC abnormalities have been linked to psychiatric and addiction disorders. Many of these mPFC-modulated functions are influenced by light exposure. However, the ability of the mPFC to encode light intensity remains unclear. **Results**: By combining extracellular recordings in awake mice with intensive neuronal mapping, we show that half of the neurons identified in the mPFC respond to light, either transiently or persistently, exhibiting a subregion-specific distribution across hemispheres and cortical layers. Persistent light-responsive neurons comprised four unique functional types of intensity-encoding neurons—two types increased while another two types decreased their firing rate in response to light-intensity steps. These functional types corresponded to mixtures of pyramidal neurons and interneurons that share response dynamics, and their firing rate closely tracked continuously increasing or decreasing light-intensity stimuli. Moreover, recording light-evoked firing in the downstream perihabenular nucleus (PHb) revealed four intensity-encoding functional types resembling their mPFC counterparts but exhibiting light-evoked firing of shorter latency and higher amplitude, persistence, and sensitivity, as well as a unique distribution of neurons across the four types. The intensity-encoding capacity of mPFC neurons depended, at least partially, on input from intrinsically photosensitive retinal ganglion cells (ipRGCs), as revealed by retinal wholemount recordings, and chemogenetic silencing and terminal ablation of ipRGCs. **Conclusions**: Together, these results demonstrate that the mPFC harbors a large diversity of excitatory and inhibitory neurons that continuously modulate their activity as a function of light intensity. This mPFC light sensitivity, which depends on ipRGC input and is likely transmitted through the dorsothalamic PHb, may represent the substrate of the effect of light on the various mPFC-modulated processes and behaviors.

### 2.70. The Impact of Blue-Light Absence in Indoor Lighting on Salivary Cortisol Levels

StebelováKatarína^1^GrácováLucia^1^KováčováKatarina^1^DzirbikovaZuzana^1^HanuliakPeter^2^HartmanPeter^2^VargovaAndrea^2^BacigálTomáš^3^HraskaJozef^2^1Department of Animal Physiology and Ethology, Faculty of Natural Sciences, Comenius University, Bratislava, Slovakia2Department of Building Structures, Faculty of Civil Engineering, Slovak University of Technology, Bratislava, Slovakia3Department of Mathematics and Descriptive Geometry, Faculty of Civil Engineering, Slovak University of Technology, Bratislava, Slovakia

**Abstract: Background**: The importance of short-wavelength lighting, especially in the morning, has been tested in laboratories and constant routine protocols, but the daylight impact on biological rhythms in real-life conditions is still not well understood. The aim of our study was to determine the effects of the reduction of in blue and green light spectra during working hours on hormone cortisol levels. **Methods**: A total of 23 young and healthy volunteers (15 women, 8 men) took part in the study. The study was performed during the winter months and took place in two identical offices, where the participants spent from 8:30–16:30 each day during control conditions (CCs) and experimental conditions (ECs) (5 days for each condition). Natural lighting conditions were not modified during CCs. In ECs, the light spectrum was changed with the foil filter up to 500 nm installed on the office windows (Orange 50 UV, KeetecFol, EU). During ECs, participants used blue light filters on all screens, even at home. On the 4th day of the CCs and ECs, volunteers were exposed to artificial light two hours before sleep in their home conditions. Five saliva samples were taken every 30 min during light exposure before sleep. Participants gave three saliva samples every morning (immediately after waking up and then one and two hours later) and another saliva sample before sleep. Cortisol levels in saliva were measured by ELISA (Arbor Assays, USA). The light conditions during the whole experiment were monitored with personal dataloggers–light watchers placed on the headband near the volunteer’s eye. **Results**: The amount of salivary cortisol was significantly lower in men than in women. We found interindividual variability in the levels of cortisol. In the morning saliva, there was a significant impact of sample collection timing. Cortisol was highest one hour after waking up in both CCs and ECs. A significant impact of short-wavelength light reduction during the day was found on the cortisol levels of men, but not women. Morning AUC of men’s cortisol was decreased by 21% (±3.13%) compared to CCs. No significant impact of light history on cortisol concentrations in evening samples was found. In women, there was an average increase of 25% (±18.87%) in cortisol compared to CCs. Men had an average decrease of 32% (±6.60%) in evening cortisol compared to CCs. Light exposure in home-based conditions did increase cortisol levels, but not significantly. In women, there was an average increase of cortisol by 145% and in men by 92% in ECs compared to light exposure in CCs. The amount of evening light did not correlate with evening cortisol concentrations. **Conclusions**: The absence of short-wavelength light during the day did affect women’s and men’s cortisol differently. The cortisol was significantly lowered in the morning saliva of men. Women did not respond to changed light conditions during the day.

**Funding**: The study was supported by grant APVV-18-0174.

### 2.71. Effect of Short-Wavelength Light Reduction during Work Hours on 6-Sulfatoxymelatonin in Morning Human Urine

DzirbikovaZuzana^1^KováčováKatarina^1^BacigálTomáš^2^HartmanPeter^3^HanuliakPeter^3^VargovaAndrea^3^HraskaJozef^3^StebelováKatarína^1^1Department of Animal Physiology and Ethology, Faculty of Natural Sciences, Comenius University in Bratislava, Bratislava, Slovakia2Department of Mathematics and Descriptive Geometry, Faculty of Civil Engineering, Slovak University of Technology, Bratislava, Slovakia3Department of Building Structures, Faculty of Civil Engineering, Slovak University of Technology, Bratislava, Slovakia

**Abstract: Background**: Change in lighting conditions in the workplace due to coloured window glazing or shading can substantially change the lighting conditions in offices. In the entrainment of the circadian system are involved intrinsically photosensitive retinal ganglion cells sensitive especially to short-wavelength light. The reduction in blue light in the workspace could have an impact on melatonin, the hormone that is produced only during the dark phase of the day. The level of 6-sulfatoxymelatonin reflects the amount of melatonin produced during the night. The aim of our study was to find out if the reduction in short-wavelength light during working hours has an impact on melatonin’s whole-night production. **Methods**: In this study, young healthy volunteers (23 volunteers—15 women, 8 men) with an average age of 26 ± 1.5 years were included. The study took place in two identical offices during the winter months (December 2020, November 2021–January 2022). In each office, participants spent 5 consecutive workdays (Monday–Friday) at work: 8:30–16:30. The participants stayed firstly in the control office, which had natural lighting conditions with full light spectrum. After the control week, participants stayed in the experimental office, where the light spectrum was changed by using orange foil that filtered the light spectrum up to 500 nm (Orange 50 UV, KeetecFol, EU). Individual lighting conditions were measured with a LightWatcher (OT sensors, Austria) placed on headbands. Three urine samples during the morning were collected from each participant (first immediately after waking up). The hormone melatonin main metabolite 6-sulfatoxymelatonin was measured by ELISA (DRG, Germany) and its levels were normalized to creatinine. **Results**: The levels of 6-sulfatoxymelatonin/creatinine in the urine (uMEL) were not significantly different in the control week in comparison with the experimental week. We correlated the mean uMEL or weighted mean uMEL with the quantity of light (sum) during the previous morning (5:00–8:30) or the previous day’s working hours (8:30–16:30). The mean uMEL did significantly correlate with morning or daylight sun. In the weighted mean uMEL, we found a significant correlation only with morning sunlight. **Conclusions**: The short-wavelength light reduction during the day did not affect the melatonin metabolite in urine. However, the significance of the light impact on uMEL was related to the used statistical approach. The understanding of the connection between personal light history and its impact on whole-night melatonin production needs to be investigated.

**Funding**: The study was supported by grant APVV-18-0174.

### 2.72. Characterizing Relationships between Sleep–Wake Rhythms and Cognition in Aging and Neurogenerative Disease

WinerJoseph R.Department of Neurology, Stanford University, Stanford, CA, USA

**Abstract:** Disrupted sleep and fragmentation of sleep–wake rhythms are common in the context of healthy aging as well as in neurodegenerative disease, resulting from a combination of physiological, societal, and behavioral factors. As wearable devices become increasingly popular among older adults, there is a need to understand how the measurement of sleep–wake behavior might be able to identify neurodegenerative disease and differentiate disease etiology and severity. This talk, focusing on healthy aging, Alzheimer’s disease, and Parkinson’s disease, will review actigraphy-estimated 24 h rhythm and sleep impairment in these populations, both in large epidemiological samples and small, deeply phenotyped cohorts. Epidemiological studies offer the possibility of understanding how patterns of activity may predict neurodegenerative disease before the onset of symptoms, whereas small studies featuring neuroimaging and other biomarkers offer a window into the neural mechanisms that underly sleep–wake disturbances. When viewed together, these frameworks offer an opportunity to utilize sleep–wake impairment as a tool for early detection and a potential treatment target.

### 2.73. Preliminary Results: Effects of Evening Smartphone Use on Sleep and Declarative Memory Consolidation in Adolescents and Young Adults

HöhnChristopher^1^HödlmoserKerstin^2^1Laboratory for Sleep, Cognition and Consciousness Research, Department of Psychology, University of Salzburg, Salzburg, Austria2Centre for Cognitive Neuroscience Salzburg (CCNS), University of Salzburg, Salzburg, Austria

**Abstract: Background**: The potential negative impact of evening short-wavelength light exposure through LED screens has received considerable media attention over the last few years. Several studies reported a reduction of melatonin secretion, often accompanied by decreased sleepiness, negative effects on sleep quality, and impaired cognitive functioning the following day. However, only the melatonin effect seems to be consistently replicable, whereas most of the additional negative consequences were observed only in some studies under specific and often artificial circumstances. Our aim was to assess the impact of evening smartphone usage on sleep in an ecologically valid study design, differentiating between adolescents and young adults. Additionally, we tried to explore potential consequences for sleep-dependent declarative memory consolidation that might accompany the sleep effects. **Methods**: We recorded full-night polysomnography data from 33 healthy male adolescents (15.64 ± 1.11 years) and 33 young adults (21.73 ± 1.92 years). All participants spent one adaptation night and three experimental nights in the sleep laboratory. During each experimental night, participants read for a total of 75 min either on a smartphone (1) without or (2) with a blue-light filter, or (3) a printed book until 45–50 min before bedtime. Salivary melatonin levels were recorded throughout the light exposure until bedtime and body temperature was measured throughout the whole night. Additionally, the subjects engaged in a declarative learning task before the light exposure with an immediate recall session and a delayed recall the next morning. **Results**: Reading on a smartphone without a blue-light filter resulted in the highest melatonin suppression effect immediately after the light exposure in both age groups. At bedtime, however, melatonin levels had significantly recovered in adolescents, but not in adults. During the night, the distal-proximal temperature gradient (DPG) was also affected differently in the two age groups. In the smartphone condition without a filter, adults had a significantly diminished DPG during the middle of the night, whereas adolescents exhibited an early peak followed by a later reduction of the DPG. Both age groups experienced a prolonged sleep latency and higher sleep fragmentation during the first quarter of the night in both smartphone conditions. However, only the adults showed a significant difference in sleep efficiency and a reduction in early deep sleep after reading on a smartphone. We did not find any differences in sleep-dependent memory consolidation (indexed by behavioral or physiological measures, e.g., sleep spindles, slow oscillations and their coupling) between the smartphone and book conditions in either age group, indexed by behavioral or physiological measures. **Conclusions**: Evening smartphone use effectively suppresses melatonin secretion in both adolescents and young adults. However, when the light exposure is stopped around 45 min before bedtime, the effects on sleep physiology are minimal and sleep-dependent memory consolidation appears to be unaffected. Adolescents recover more quickly from the melatonin suppression, but further research is needed to determine whether the light effects might be more severe if the smartphone is used immediately before going to sleep.

### 2.74. Digital Circadian Health toward Personalized Cancer Chronotherapy

LéviFrancis^1^,^2^Finkenstädt RandBärbel^3^BalpRodrigo^4^AdamRené^5^BouchahdaMohamed^1^1Research Unit “Chronotherapy, Cancers and Transplantation,” and Medical Oncology Service, Paul Brousse Hospital, and Faculty of Medicine, Paris Saclay University, Villejuif, France2Medical School, University of Warwick, Coventry, UK3Department of Statistics, University of Warwick, Coventry, UK4Future of Healthcare Program Research and Innovation Direction, Capgemini Engineering, Meudon, France5Research Unit “Chronotherapy, Cancers and Transplantation”, and Hepato-Biliary Center, Paul Brousse Hospital, and Faculty of Medicine, Paris Saclay University, Villejuif, France

**Abstract: Background**: An increased risk of cancer has been linked to prolonged night work by the International Agency for Research on Cancer, with such association being consistently rated as close to full evidence (Lancet Oncol 2010 and 2019). Recent systematic reviews and/or meta-analyses have highlighted statistically significant and clinically relevant improvements in tolerability and/or efficacy of chemotherapy, immunotherapy and radiotherapy through chronomodulated delivery or selected time of day of administration (Printezi et al. TLO 2022; Landre et al. ASCO 2023; Bermudez-Guzman et al. Front Oncol 2021). Yet, large between-subject differences characterize both the risk of cancer upon night work exposure and the therapeutic improvements from cancer chronotherapy, as well as circadian timing system (CTS) dynamics. **Methods**: CTS traceability could be achieved through a digital platform providing real-time information from non-invasive circadian biomarkers, thus allowing for traceable real time chronopreventive or chronotherapeutic intervention. We have designed and constructed such an experimental mobile telemonitoring platform aiming at real-time measurements of circadian and sleep parameters in remote people during their daily life. We are now discovering its potential for the integration of circadian concepts in occupational medicine and oncology. **Results**: In a cross-sectional epidemiologic study (140 hospital day- or night-shift workers), rest–activity, body temperature and sleep cycles were telemonitored for a week. Between-subject differences in circadian and sleep responses to night work were identified. The CTS imprinting of past night work exposure supported telemonitored circadian biomarkers as surrogates of cancer risk in individual shift workers (Zhang et al. eBiomedicine 2022). Prior circadian telemonitoring studies in healthy or cancer patients have led us to investigate the potential of rhythm telemonitoring for securing frail cancer patients at home. The ongoing multicenter MultiDom trial (*NCT04263948*) aims at reducing the risk of emergency admissions in patients receiving a conventional mFOLFIRINOX chemotherapy protocol for advanced pancreatic cancer. Physical activity (accelerometry), and body temperature are measured q1 min using a continuously worn telecommunicating chest surface sensor, daily body weight is self-measured with a telecommunicating balance, and daily symptoms are self-rated using an electronic tablet for one week before and six weeks on chemotherapy for 67 patients. Data are continuously tele-transmitted to an ad hoc platform version set within the French National Health Data Hub. Physical activity, sleep, fever, circadian rhythms, body weight, and symptoms severity dynamics of each patient are visualized daily by the oncology teams on screen displays. Item-specific alerts are automatically generated and sent to the prespecified health professionals, with traceable follow-up of resulting interventions in the patient’s record. Excellent performance and compliance together with illustrative cases of between- and within-variabilities in individual patients’ circadian biomarkers support the concept of personalized treatment dose, schedule and timing adjustments using datasets from the first 30 registered patients. **Conclusions**: Such a digital circadian medicine platform could represent a useful tool for improving well-being and patient conditions through advancing precision and personalized circadian medicine.

### 2.75. Beyond Day and Night: Effects of Light Exposure on Sleepiness and Sleep

LokRenske^1^ZeitzerJamie^1^HutRoelof^2^1Department of Psychiatry and Behavioral Sciences, Stanford University, Stanford, CA 94305, USA2University of Groningen, Groningen, The Netherlands

**Abstract:** Non-image-forming effects of light have been readily assessed on a variety of physiological processes, including melatonin suppression, the phase angle of entrainment, and alertness. One of the processes that is less well characterized is the effects of light on sleep. Light exposure during wakefulness has been shown to affect subsequent sleep, such that light directly before bedtime reduces sleep quality and sleepiness under real-life conditions. Recent literature, however, suggests that light exposure at different times of day can also benefit sleep quality. In a series of experiments, we aimed to determine the complex relationship between light exposure and sleep quality. We investigated how self-reported sleep quality relates to measured sleep quality (polysomnography), if increased light exposure during wakefulness can improve sleep quality, and if such light effects depend on the circadian clock phase or sleep pressure buildup. This talk will provide insights into the complex relationship between light exposure and sleep quality beyond the traditional dichotomy of day and night.

### 2.76. Technologies to Reliably Determine Melatonin and DLMO

WeberJackyEstradaAlejandro FernandezNovoLytiX GmbH, Bättwil, Switzerland

**Abstract:** Soon after the first isolation and description of melatonin by Aaron Lerner in 1957, it was attempted to quantify this molecule in different body fluids of humans and animals as well as in many other biological and environmental samples. The very first method described to determine concentrations of melatonin was a microscopic bioassay by analysing the darkening and/or lightening of frog skin after contact with melatonin containing samples. Mori and Lerner were able to detect as low as 10 pg of melatonin using this method. In the sixties and early seventies, a series of quantitative bioassays and paper chromatographic (separation) methods were successfully established. Between 1964 and 1999, several gas chromatography (GC) methods without and without mass spectrometry (GC-NCI-MS) were established. In the seventies and eighties, some high-performance liquid chromatography (HPLC) methods were also developed, but these are lacking enough sensitivity (in best case 10–50 pg/mL) for reliable determination human and animal plasma or saliva day–night profiles. in the early 2000s, the first tandem mass spectrometry methods combined with liquid chromatography (LC-MS/MS) were described for the determination of melatonin in human saliva with quantitation limits around 3 pg/mL (e.g., Eriksson et al., 2003). In the following years, the LC-MS/MS methods were further elaborated and optimized for the reliable measurement of human plasma and saliva samples, reaching a sensitivity of the ultimate immunoassay methods at limits of quantitation of around 1 pg/mL for saliva and 3 pg/mL for plasma (e.g., van Faassen et al., 2017). Since the first immunoassay based on an antibody-radiolabeled antigen reaction to quantify plasma insulin was reported by Berson and Yalow (1959), it was obvious to also measure melatonin with this novel technology. However, it needed about 15 years until the first radioimmunoassays (RIAs) were developed, first based on tritiated (^3^H) (e.g., Arendt et al., 1975; Levine and Riceberg, 1975), then based on iodinated (^125^I) melatonin tracers (e.g., Rollag and Niswender, 1976; Geffard et al., 1982; Vakkuri et al., 1984). Another 15 to 20 years later, non-radioactive ELISAs (enzyme linked immunosorbent assays) appeared, first for direct saliva measurements (some assays only work after saliva pretreatment), then for plasma samples, but only after sample extraction. However, until very recently, ELISA methods never reached the sensitivity or precision in the lower measuring ranges compared to the best available RIAs (comprehensively reviewed in Kennaway, 2019). Nowadays, it is recommended to use either the best performing immunoassays or the most modern LC-MS/MS methods to reliably assess dim-light melatonin onset (DLMO) which became the most relevant circadian phase marker. Pros and cons of both assay technologies will be discussed. Furthermore, several methods for the determination of DLMO will be shown and critically challenged. Last but not at least, the pre-analytical processes, sample stability as well as issues of using better plasma than saliva for the DLMO assessment will be highlighted.

### 2.77. Optimizing Tunable Lighting for Human Health

HanifinJohn^1^WarfieldBenjamin^1^KempJohn^1^JasserSamar^1^NelsonNicolas^1^YooTaehwan^1^KarlicekRobert^2^DauchyRobert^3^BlaskDavid^3^BrainardGeorge^1^1Thomas Jefferson University, Philadelphia, PA, USA2Rensselaer Polytechnic Institute, Troy, NY, USA3Tulane University, New Orleans, LA, USA

**Abstract: Background**: The US Department of Energy anticipates that by 2030, LEDs will reach 80% of all lighting sales, saving US $26 billion per year in electricity costs. The adoption of color-tunable LED systems may reduce some of the overall solid-state lighting (SSL) efficiency under the guise of providing improved health and wellness to occupants. There remains, however, a lack of data regarding the possible positive impacts of daytime tunable SSL lighting on health and wellness, as realized in our recent research animal studies. To address this gap, research must be done to quantify characteristics of human metabolic, endocrine and sleep physiology affected by tunable SSL versus typical fluorescent lighting. **Methods**: The goal of this project is to test the efficacy of tunable SSL inclusive of bright short-wavelength enriched SSL versus dimmer fixed spectrum cool white fluorescent (CWF) lighting on a range of metabolic (glucose, insulin, leptin), endocrine (cortisol, melatonin) and sleep (onset, duration, efficiency) physiology in healthy human adults. We will run two studies testing the hypothesis that, compared to dimmer, commonly used CWF lighting, tunable lighting inclusive of bright short-wavelength enriched SSL and dimmer, short-wavelength depleted SSL will improve participants’ overall health as measured by metabolic, endocrine and sleep physiology. One study (N = 12, crossover) will be performed over 7 days in a completely controlled laboratory setting. The other study will include conditions that are more naturalistic. The 9-day naturalistic study (N = 28) will have a hybrid of controlled exposure to SSL during an 8 h daytime work period, followed by subjects leaving the laboratory for the rest of the day to receive light exposure in their chosen public and domestic settings. Both studies are intended to add evidence relevant to the purported benefits of tunable SSL in indoor lighting environments. **Results**: The studies started in June 2023. We hypothesize that, compared to static, dimmer, daily lighting of CWF lamps tunable bright short-wavelength enriched SSL will increase the amplitude and duration of melatonin production, advance onset of melatonin secretion, optimize amounts of glucose, insulin, leptin and cortisol, shorten sleep latency and improve sleep efficiency. **Conclusions**: The goal of this project is to characterize the potential link between lighting and human health for users of SSL tunable lighting systems using quantified endpoints of metabolism, endocrine function, and sleep physiology.

**Keywords:** light; sleep; melatonin; cortisol; glucose; insulin; leptin; solid-state lighting

**Funding**: Primary funding: DOE Agreement #DE-EE0009689.

### 2.78. Light Sensitivity in the Nucleus Accumbens and Dorsomedial Striatum

ZangenElyashivSchulzNadavCohen-HatabDanielObeidMustafaDobrotvorskiaIrinaSabbahShaiDepartment of Medical Neurobiology, Faculty of Medicine, The Hebrew University of Jerusalem, Jerusalem, Israel

**Abstract: Background**: Abnormal lighting triggers depression, while light therapy, in conjunction with pharmacotherapy, relieves it. A recently identified pathway that appears to mediate an effect of light on mood includes intrinsically photosensitive retinal ganglion cells (ipRGCs) that innervate the dorsothalamic perihabenular nucleus (PHb), which projects to the medial prefrontal cortex (mPFC), nucleus accumbens (NAc), and dorsomedial striatum (dmStr). While this may possibly confer these brain regions light sensitivity, the direct light-evoked neuronal responses in the NAc and dmStr have not been investigated. **Results**: We used extracellular recordings of single neurons in awake mice, exposing their eyes to diffused light at various intensities. We identified over 1000 neurons in the NAc and dmStr, with over half of these showing statistically significant responsiveness to light. Six distinct light-responsive neuronal types were identified. These types, which showed specific spatial distributions across the NAc and dmStr, differed in their response polarity and kinetics. Some neurons increased their firing at light onset, offset, or both, while others suppressed their firing at light onset. Interestingly, certain neurons displayed transient responses, while others exhibited sustained responses similar to those of ipRGCs. A fraction of those neurons monotonically varied their firing rate in respect to light intensity, suggesting their ability to encode the intensity of ambient light. **Conclusions**: Our work reveals that the NAc and dmStr, which are not typically associated with light sensitivity, contain a diverse population of light-responsive neurons, with some of these modulating their firing rate in respect to light intensity. These findings raise the intriguing possibility that light may regulate various functions supported by the NAc and dmStr, which in addition to mood include learning, habit formation, decision-making, and psychiatric disorders such as addiction, OCD, and schizophrenia.

**Keywords:** nucleus accumbens; dorsal striatum; perihabenular nucleus; intrinsically photosensitive retinal ganglion cells (ipRGCs); depression; addiction

### 2.79. Targeting the Intestinal Circadian Clock for Disease Prevention

NiuYunhui^1^,^2^HeddesMarjolein^1^,^2^AltahaBaraa^1^,^2^BirknerMichael^2^KleigreweKarin^3^MengChen^3^HallerDirk^1^,^2^KiesslingSilke^1^,^2^,^4^†1ZIEL—Institute for Food and Health, Technical University of Munich, 85354 Freising, Germany2School of Life Sciences, Technical University of Munich, Gregor-Mendel-Str. 2, 85354 Freising, Germany3Bavarian Center for Biomolecular Mass Spectrometry, Technical University of Munich, Gregor-Mendel-Str. 4, 85354 Freising, Germany4Faculty of Health and Biomedical Science, University of Surrey, Stag Hill Campus, Guildford GU2 7XH, UK†Presenting Author: Silke Kiessling; s.kiessling@surrey.ac.uk.

**Abstract: Objective**: Impaired clock-gene expression has been observed in biopsy samples from patients with inflammatory bowel disease (IBD). Disruption of circadian rhythms, which occurs in shift workers, has been linked to an increased risk of gastrointestinal diseases, including IBD. The intestinal clock balances gastrointestinal homeostasis by regulating the microbiome. Here, we characterize intestinal immune functions in mice lacking the intestinal clock and an IBD-relevant mouse model under different feeding conditions to describe the functional impact of the intestinal clock in the development of gastrointestinal inflammation. Design tissues and fecal samples from intestinal clock-deficient mice (Bmal1IEC−/−) and mouse models for colitis (IL-10−/−, Bmal1IEC−/− xIL-10−/−, dextran sulfate sodium (DSS) administration) under ad libitum and restricted feeding (RF) conditions were used to determine the causal role of the intestinal clock for colitis. **Results**: In IL-10−/− mice, inflammation correlated with disrupted colon clock genes expression. Genetic loss of intestinal clock functions promoted DSS and IBD inflammatory phenotypes and dramatically reduced survival, and colonization with disease-associated microbiota in germ-free Bmal1IEC−/− hosts increased their inflammatory responses, demonstrating the causal role of colonic clock disruption and the severity of IBD. RF in IL-10−/− mice restored the colon clock and related immune functions, improved the inflammatory responses and rescued the histopathological phenotype. In contrast, RF failed to improve IBD symptoms in Bmal1IEC−/− xIL-10−/− demonstrating the significance of the colonic clock in gating the effect of RF. **Conclusions**: We provide evidence that inflammation-associated intestinal clock dysfunction triggers host immune imbalance and promotes the development and progression of IBD-like colitis. Enhancing intestinal clock function by RF modulates the pathogenesis of IBD and thus could become a novel strategy to ameliorate the symptoms in IBD patients.

